# Supplementary material for: Prevalence of child maltreatment in India and its association with gender, urbanisation and policy: a rapid review and meta-analysis protocol
Source: BMJ Open. 2021 Aug 8;11(8):e044983. doi: 10.1136/bmjopen-2020-044983 (PMC8354262; doi:10.1136/bmjopen-2020-044983)
Supplement: Supplementary data [file bmjopen-2020-044983supp001.pdf]

Appendix 1 - Experts in the field who contributed to the research study design

Member, Designation & Location	Responsibilities
**Nilakshi Vaidya, Psychology Counsellor, National Institute of Mental Health and Neurosciences (NIMHANS) (India) –** 2nd reviewer	Will be the second reviewer for the systematic review and will review 20% of the article titles, abstract and full texts for inclusion criteria. Will be the second author on any journal paper published.
**Megan Fernandes, Core Surgical Trainee with experience working in pediatric medicine and surgery (UK)-**3rd reviewer	Will be the third reviewer for the systematic review and will review 20% of the article titles, abstract and full texts for inclusion criteria (published pre-2012). Will be the third author on any journal paper published.
**Phillip De Souza, Core Internal Medicine Trainee, experience working in internal medicine (UK) -**4th reviewer	Will be the fourth reviewer for the systematic review and will review 20% of the article titles, abstract and full texts for inclusion criteria (published post-2012). Will be the fourth author on any journal paper published.
**Vikas Choudhry, Director, Vice President of Public Health, SRC (Uttar Pradesh)**	Regular contact through the systematic review process. Will input on search strategies and will specifically resolve any conflicts between myself and the second reviewer.<br>Will be the final author (senior author) on any journal publication.
**Bharath Holla, Clinical Psychiatrist and Associate Professor, NIMHANS (India)**	PPI group for input on search words and search strategies
**Vivek Benegal, Clinical Psychiatrist and cVEDA Principal Investigator, NIMHANS (India)**	Clinical psychiatrist and expert in child trauma and informing trauma-based policies in India. Regular contact through the review process and specific input on the search strategies and key words and MESH terms used. Penultimate senior author on any publication.

Fernandes G, *et al. BMJ Open* 2021; 11:e044983. doi: 10.1136/bmjopen-2020-044983
