# Supplementary material for: Prevalence of child maltreatment in India and its association with gender, urbanisation and policy: a rapid review and meta-analysis protocol
Source: BMJ Open. 2021 Aug 8;11(8):e044983. doi: 10.1136/bmjopen-2020-044983 (PMC8354262; doi:10.1136/bmjopen-2020-044983)
Supplement: Supplementary data [file bmjopen-2020-044983supp002.pdf]

Appendix 2a. PubMed search strategy

a) Child sexual abuse:

(((((((child sexual abuse) OR child sexual coercion) OR child sexual offender)) OR (((child sexual assault) OR child sexual porn*) OR child sexual molest*)) OR (((((child sexual crime) OR child sexual aggression) OR child sexual offence) OR child sexual violence) OR child rape)) OR ((child rape) OR child incest)) AND India

b) Child emotional abuse:

((((((((((child worthless) OR child unloved) OR child inadequate) OR child failure) OR child corrupt*) OR child unheard) OR child cruelty) OR child emo* AND abuse) OR child maltreat*) AND India)) AND ((((((((((((child denial*) OR child denial*) OR child denial shelter*) OR child carer failure) OR child parental failure) OR child emo*) OR child emo* AND danger) OR child neglect) OR child abandonment*) OR child bereavment*) OR child grief) AND India[all])

c) Child physical abuse:

(((((((((((child hitting) OR child shaking) OR child throwing) OR child poisoning) OR child burning) OR child scalding) OR child drowning) OR child suffoc*) OR child beating) OR child physical violence) OR child past*) OR child rap) AND India

Child neglect:

d) (((((child neglect) OR child psycho* AND assault) OR child denial) OR child emo* AND assualt) OR child malnu*) AND India

Appendix 2b EMBASE search strategy

a) Child sexual abuse

1. Prevalence/
2. Limit 1 to human
3. (“India” or “india”).mp.[mp=title, abstract, heading word, drug trade name, original title, device manufacturer, drug manufacturer, device trade name, keyword, floating subheading word]
4. (child sexual abuse or “child sexual coercion” or “child sexual offender” or “child sexual assault” or “child sexual porn*” or “child sexual molest*” or “child sexual crime” or “child sexual aggression” or “child sexual offence”) or “child sexual violence” or “child rape” or “child rape” or “child incest”).mp. [mp=title, abstract, heading word, drug trade name, original title, device manufacturer, drug manufacturer, device trade name, keyword, floating subheading word]
5. 1 and 2 and 3 and 4
6. Limit 5 to yr= “2005 to 2020”

b) child emotional abuse

1. Prevalence/
2. Limit 1 to human
3. (“India” or “india”).mp.[mp=title, abstract, heading word, drug trade name, original title, device manufacturer, drug manufacturer, device trade name, keyword, floating subheading word]
4. (child worthless or “child unloved“ or “child inadequate“ or “child failure“ or “child corrupt“ or “child unheard“ or “child cruelty“ or “child emo*“ or “child emo abuse“ or “child maltreat“ or “child denial“ or “child denial shelter“ or “child carer failure“ or “child parental failure“ or “child emo*“ or “child emo* and danger“ or “child neglect“ or “child abandonment*“ or “child bereavment*“ or “child grief“)mp.[mp=title, abstract, heading word, drug trade name, original title, device manufacturer, drug manufacturer, device trade name, keyword, floating subheading word]
5. 1 and 2 and 3 and 4
6. Limit 5 to yr= “2005 to 2020”

c) Child physical abuse

1. Prevalence/
2. Limit 1 to human
3. (“India” or “india”).mp.[mp=title, abstract, heading word, drug trade name, original title, device manufacturer, drug manufacturer, device trade name, keyword, floating subheading word]
4. (child hitting or ”child shaking” or ”child throwing” or ”child poisoning” or ”child burning” or ”child scalding” or ”child drowning” or ”child suffoc*” or ”child beating” or ”child physical violence” or ”child past*” or ”child rap”)mp.[mp=title, abstract, heading word, drug trade name, original title, device manufacturer, drug manufacturer, device trade name, keyword, floating subheading word]
5. 1 and 2 and 3 and 4
6. Limit 5 to yr= “2005 to 2020”

d) Child neglect
   1. Prevalence/
   2. Limit 1 to human
   3. ("India" or "india").mp.[mp=title, abstract, heading word, drug trade name, original title, device manufacturer, drug manufacturer, device trade name, keyword, floating subheading word]
   4. (child neglect or "child psycho*" or "assault" or "child denial" or "child emo*" or "assault*" or "child malnu")mp.[mp=title, abstract, heading word, drug trade name, original title, device manufacturer, drug manufacturer, device trade name, keyword, floating subheading word]
   5. 1 and 2 and 3 and 4
   6. Limit 5 to yr= "2005 to 2020"

Appendix 2c COCHRANE search strategy

1. India or india
2. (child sexual abuse or child sexual coercion or child sexual offender or child sexual assault or child sexual porn or child sexual molest* or child sexual crime or child sexual aggression or child sexual offence or child sexual violence or child rape or child rape or child incest): ti,ab,kw
3. (child worthless or child unloved or child inadequate or child failure or child corrupt or child unheard or child cruelty or child emo* or child emo abuse or child maltreat or child denial or child denial shelter or child carer failure or child parental failure or child emo* or child emo* and danger or child neglect or child abandonment* or child bereavment* or child grief): ti,ab,kw
4. (child hitting or child shaking or child throwing or child poisoning or child burning or child scalding or child drowning or child suffoc* or child beating or child physical violence or child past* or child rap): ti,ab,kw
5. (child neglect or child psycho* or assault or child denial or child emo* or assault* or child malnu*): ti,ab,kw
6. {#2 or #3 or #4 or #5}
7. #1 and #6

Appendix 2d PsychInfo search strategy

1. Prevalence or epidemiology or frequency.tw.
2. child sexual abuse or child sexual coercion or child sexual offender or child sexual assault or child sexual porn or child sexual molest* or child sexual crime or child sexual aggression or child sexual offence or child sexual violence or child rape or child rape or child incest.tw,sh.
3. child worthless or child unloved or child inadequate or child failure or child corrupt or child unheard or child cruelty or child emo* or child emo abuse or child maltreat or child denial or child denial shelter or child carer failure or child parental failure or child emo* or child emo* and danger or child neglect or child abandonment* or child bereavment* or child grief.tw,sh.
4. child hitting or child shaking or child throwing or child poisoning or child burning or child scalding or child drowning or child suffoc* or child beating or child physical violence or child past* or child rap.tw,sh.
5. child neglect or child psycho* or assault or child denial or child emo* or assault* or child malnu*.tw,sh.
6. India
7. 1 or 2 or 3 or 4 or 5
8. 7 and 6
9. Limit 8 to (English language and yr=”2005-2020”)
