# Supplementary material for: Prevalence of child maltreatment in India and its association with gender, urbanisation and policy: a rapid review and meta-analysis protocol
Source: BMJ Open. 2021 Aug 8;11(8):e044983. doi: 10.1136/bmjopen-2020-044983 (PMC8354262; doi:10.1136/bmjopen-2020-044983)
Supplement: Author's manuscript [file bmjopen-2020-044983.draft_revisions.pdf]

BMJ Open is committed to open peer review. As part of this commitment we make the peer review history of every article we publish publicly available.

When an article is published we post the peer reviewers' comments and the authors' responses online. We also post the versions of the paper that were used during peer review. These are the versions that the peer review comments apply to.

The versions of the paper that follow are the versions that were submitted during the peer review process. They are not the versions of record or the final published versions. They should not be cited or distributed as the published version of this manuscript.

BMJ Open is an open access journal and the full, final, typeset and author-corrected version of record of the manuscript is available on our site with no access controls, subscription charges or pay-per-view fees (http://bmjopen.bmj.com).

If you have any questions on BMJ Open's open peer review process please email info.bmjopen@bmj.com

BMJ Open

The prevalence of child maltreatment in India and its association with gender, urbanisation and policy: a systematic review and meta-analysis protocol

Journal:	*BMJ Open*
Manuscript ID	bmjopen-2020-044983
Article Type:	Protocol
Date Submitted by the Author:	28-Sep-2020
Complete List of Authors:	Fernandes, Gwen; University of Bristol, Population Health Sciences<br>Fernandes, Megan; Norfolk and Norwich University Hospital NHS Trust, Department of Surgery<br>Vaidya , Nilakshi ; National Institute of Mental Health and Neuro Sciences<br>De Souza, Philip; Norfolk and Norwich University Hospital NHS Trust, Department of Medicine<br>Plotnikova, Evgeniya; The University of Edinburgh Usher Institute of Population Health Sciences and Informatics<br>Geddes, Rosemary; The University of Edinburgh Usher Institute of Population Health Sciences and Informatics<br>Holla, Bharath; National Institute of Mental Health and Neurosciences<br>Sharma, Eesha ; National Institute of Mental Health and Neuro Sciences, Child And Adolescent Psychiatry<br>Benegal, Vivek; National Institute of Mental Health and Neurosciences<br>Choudhry , Vikas; Sambodhi Research and Communications Pvt Ltd., Public Health
Keywords:	EPIDEMIOLOGY, Protocols & guidelines < HEALTH SERVICES ADMINISTRATION & MANAGEMENT, MENTAL HEALTH, PUBLIC HEALTH

SCHOLARONE™
Manuscripts

I, the Submitting Author has the right to grant and does grant on behalf of all authors of the Work (as defined in the below author licence), an exclusive licence and/or a non-exclusive licence for contributions from authors who are: i) UK Crown employees; ii) where BMJ has agreed a CC-BY licence shall apply, and/or iii) in accordance with the terms applicable for US Federal Government officers or employees acting as part of their official duties; on a worldwide, perpetual, irrevocable, royalty-free basis to BMJ Publishing Group Ltd ("BMJ") its licensees and where the relevant Journal is co-owned by BMJ to the co-owners of the Journal, to publish the Work in this journal and any other BMJ products and to exploit all rights, as set out in our licence.

The Submitting Author accepts and understands that any supply made under these terms is made by BMJ to the Submitting Author unless you are acting as an employee on behalf of your employer or a postgraduate student of an affiliated institution which is paying any applicable article publishing charge ("APC") for Open Access articles. Where the Submitting Author wishes to make the Work available on an Open Access basis (and intends to pay the relevant APC), the terms of reuse of such Open Access shall be governed by a Creative Commons licence – details of these licences and which Creative Commons licence will apply to this Work are set out in our licence referred to above.

Other than as permitted in any relevant BMJ Author's Self Archiving Policies, I confirm this Work has not been accepted for publication elsewhere, is not being considered for publication elsewhere and does not duplicate material already published. I confirm all authors consent to publication of this Work and authorise the granting of this licence.

For peer review only

Systematic Review Protocol Paper

The prevalence of child maltreatment in India and its association with gender, urbanisation and policy: a systematic review and meta-analysis protocol

Gwen Sascha Fernandes[*1], Megan Fernandes*[*2], Nilakshi Vaidya[3], Phillip De Souza[4], Evgeniya Plotnikova[5], Rosemary Geddes[5], Bharath Holla[3], Eesha Sharma[3], Vivek Benegal[3], Vikas Choudhry[6]*
*denotes joint first authorship

1. Population Health Sciences, Bristol Medical School, University of Bristol, UK
2. Department of General Surgery, Norfolk and Norwich University Hospital, Norwich, UK
3. The National Institute of Mental Health and Neurosciences, Bangalore, India
4. Department of Medicine, Norfolk and Norwich University Hospital, Norwich, UK
5. Usher Institute, School of Medicine, University of Edinburgh, UK
6. Sambodhi Research and Communications Pvt Ltd

Corresponding Author Details:
Dr Gwen Fernandes; gwen.fernandes@bristol.ac.uk
Population Health Sciences
Bristol Medical School,
Barley House
University of Bristol
BS8 2BN

Keywords: Adverse Childhood Experiences, India, Child Abuse, Child Neglect, Adversity

Word Count: 3890

For peer review only

Abstract

Introduction: India is home to 20% of the world's children and yet, little is known on the magnitude and trends of child maltreatment nationwide. The aims of this systematic review are to provide a prevalence of child maltreatment in India with considerations for any effects of gender; urbanisation (e.g. urban versus rural); and, legislation (Protection of Children from Sexual Offences Act 2012).

Methods and Analysis: A systematic review will be undertaken of all quantitative peer-reviewed studies on child maltreatment in India between 2005 and 2020. Four electronic databases will be systematically searched: PubMed, EMBASE, Cochrane and PsychIndex. The primary outcomes will include all aspects of child maltreatment: physical abuse, sexual abuse, emotional abuse, emotional neglect and physical neglect. Study participants will be between 0-18 years and will have reported maltreatment experiences using validated, reliable tools such as the Adverse Childhood Experiences Questionnaire as well as child self-reports and clinician reports. Methodological appraisal of the studies will be assessed by the Newcastle-Ottawa Quality assessment scale. Also, if sufficient data are available, a meta-analysis will be conducted. Effect sizes will be determined from random-effects models stratified by gender, urbanisation and pre-and post the 2012 POCSO Act cut off.

Ethics and Dissemination: As this is a systematic review, minimal ethical risks are expected. A Level 1 self-audit checklist has been approved by an ethics panel in the Usher Institute at the University of Edinburgh. Findings from this review will be disseminated widely through peer-reviewed publication and in various media, for example, conferences, congresses or symposia.

Registration: A study protocol was developed before starting this systematic review and was registered with the international prospective register of systematic reviews (PROSPERO) (registration number CRD42019150403)

Article Summary

- There is a lack of research on adverse childhood experiences from lower-and middle-income countries like India. This protocol highlights the process for identifying current prevalence rates for child maltreatment across the Indian subcontinent.
- Previous research has focussed on child sexual abuse and not on co-existing early childhood adversities such as physical abuse and emotional neglect.
- The study will evaluate the effect of gender, urbanisation (e.g. rural or slum) and legislation on child maltreatment rates in India between 2005 and 2020.
- Evaluation of impact of the only legislation implemented to counter child sexual abuse: Protection of Children from Sexual Offences Act 2012.

For peer review only

Introduction

In the past two decades, significant progress has been made in reducing child deaths. UNICEF estimates that, worldwide, there were 12.6 million children who died prematurely before the age of five in 1990 and this has declined to 5.3 million in 2018 [1]. Of the children who do survive, 200 million do not reach their full potential due to a combination of family socioeconomic status, geographic location, ethnicity, disability or religious orientation [1]. The early years of childhood provide the basis of intelligence and learning, personality and self-motivation, social behaviour and collaboration, which extend into adulthood [2].

The World Health Organization (WHO) defines child maltreatment as 'the abuse and neglect that occurs to children under 18 years of age and includes all types of physical and/or emotional ill-treatment, sexual abuse, neglect which results in harm'. The WHO reports that worldwide, child maltreatment is widespread with 1 in 4 adults reporting being physically abused as children [3,4]. Research suggests that children who have experienced such adversity do not receive the right nutrition, care or opportunities to learn and grow as children in more affluent geographical areas or economies [5,6]. Research on early childhood adversity have been limited in lower-and middle- income countries (LMICs) like India, with a pronounced lack of cross-sectional and longitudinal studies [7]. These adversities such as abuse or neglect, along with associated habits such as underage smoking or drinking, formed in childhood, have been linked to a range of poor physical and mental health outcomes throughout the life course and ultimately greater premature mortality [8]. Childhood adversities and health consequences can be exacerbated by socioeconomic status, urbanisation (rural areas or slum areas compared with urban zones) or even caste [9]. In her book, Bitter Chocolate [10], Pinki Virani discusses the context of these adversities in India – a highly patriarchal culture in which reprimanding, punishing or spanking a child (physically abusive behaviour) is a cultural norm [11]. The Indian economy is further characterised by external adversities - from a rapidly growing economy; stark urban-rural divides; religious strife; and, geopolitical upheavals [12]. Data from UNICEF and the World Bank [13] shows a global perspective of child rights and Indian children are marked as experiencing a difficult situation as categorised by their rights, access to education and healthcare and exposure to child labour and child marriages (Figure 1). It is no surprise that physiological and neurobiological studies have found that adversities can lead to changes of neural networks, impaired nervous, endocrine and immune systems development, resulting in physical, mental and emotional dysfunction and chronic physiological damage [14-16].

An Indian context

In India, home to 19% of the world's children, it is estimated every second child is exposed to sexual abuse and violence [17]. The Indian National Crimes Records Bureau (NCRB) report a child is sexually abused every 15 minutes and 53% of children report abuse by a parent, relative or schoolteacher [18]. The prevalence of child sexual abuse (CSA) in high-income countries is 20% for women and 8% for men [19] but in India, the estimates vary between 4-66% for women and 4-57% for men [20,21]. These figures are considered underestimates because of limited surveillance of direct adverse childhood experiences (ACEs) such as physical abuse or emotional abuse as well as CSA due to stigma particularly affecting girls; underreporting of cases by healthcare and police authorities; stigma and taboo associated with being a victim or

For peer review only

experiencing abuse, and varying prevalence rates by geography (e.g. urban versus rural versus slum settings) as well as varying communities (school-based, hospital based, trafficking victims etc) [22-25].

Previous research has demonstrated that ACEs rarely occur in isolation and in fact, have a tendency to cluster [7]. Community-based studies have reported the prevalence and overlap of childhood sexual, physical and emotional abuse, but in India this has been limited. Choudhry's 2018 systematic review focussed on one aspect of child maltreatment: CSA, and was seminal work in detailing the high prevalence rates of CSA in Indian children [20]. However, the authors acknowledged that CSA does not occur in silos and often co-occurs with other forms of child maltreatment in the same child [26].

Adverse Childhood Experiences in India

Adverse childhood experiences (ACEs) include direct, indirect and extended harms to children (Figure 2, [20, 27, 28]. Direct ACEs meet the definition for child maltreatment (red arm, Figure 2), each having significant ramifications extending from childhood to adulthood and beyond – affecting the family and society as a whole [29]. In India, data on ACEs is limited although a few recent studies have been able to identify the prevalence of ACEs in specific communities [20]. Damodaran and Paul [30] compiled data on ACEs in youth based in Kerala (South India) and reported that in a sample of 600 young people, 91% had reported at least one ACE and over half the population reported three or more ACEs. However, in terms of severity, males reported exposure to more severe ACEs than females such as physical and sexual abuse. Another study found that 57% of 559 youth (also from Kerala) had three or more ACEs where emotional abuse was the most commonly occurring ACE.

In India, lower incomes, poor living conditions and living with domestic violence are indirect ACEs that can lead to direct ACEs such as neglect and abuse and often 'cluster' [31]. Figure 3 combines the socioecological model of child maltreatment [32] and suggests potential pathways (e.g. from a macrosystem/societal or exosystem/community level) that may contribute to child maltreatment [33,34]. Particularly, around the individual level factors – there is a gap in the rates of other types of child maltreatment associated with CSA (e.g. emotional abuse or neglect). Whilst the topic of ACEs in India has received limited attention, child sexual abuse (CSA) has received considerable attention from academics, clinicians, policymakers and legislators in recent years [20, 35]. With the high prevalence of all types of child abuse in India, an attempt to amalgamate and compile the findings and present these figures in a systematic, cohesive and coherent manner is pressing [1].

Effect of Gender

Previous research has suggested the nuanced effect of gender on the occurrence of child maltreatment in India [20]. In India, gender identification is illegal and yet, almost half of girls 'wish they were born boys' [36]. Miller and colleagues [37] found that gender inequity norms were significantly associated with sexual abuse in an interventional study aimed at promoting gender equality among adolescent males. Although anecdotal evidence and case reports suggest that prevalence of sexual abuse is higher in female children than in male children, based on hospital services or

For peer review only - http://bmjopen.bmj.com/site/about/guidelines.xhtml

care services utilisation [38], some studies shows equal rates of sexual abuse across gender [36, 39]. Deb [40] suggests that boys may be experiencing and reporting more sexual abuse whilst girls are less likely to be aware of abuse (knowledge and attitudes) or less likely to report abuse for fear of shame or stigma. However, when it comes to neglect with regards to food and education security, girls are known to be disadvantaged [41]. India is the only country where girls have greater rates of under-five mortality compared with boys. Indian girls are breastfed for shorter periods and consume less milk (ibid). The reasons for this discrepancy are varied: there is a preference for sons over daughters in India and as an extension, more opportunities with regards to food, healthcare and education are afforded to male children [42]. However, given the complexities of gender effects in different types of abuse, generalisations cannot be drawn due to the limited number of studies, methodological limitations (e.g. small sample sizes, non-generalisability of findings to the wider Indian diaspora), urbanisation and socioeconomic privilege (e.g. remote tribal areas being worse affected than wealthier urban ones [43], and reporting bias (e.g. particularly if parents reported via food diaries what was consumed).

Effect of Urbanisation

Only two studies [44,45] included community populations from rural areas as well as urban areas. Whilst other cross-sectional studies have been conducted in urban areas of India [46], the child maltreatment prevalence figures (32% of boys and 42% of girls) are higher but not comparable due to differences in sample size; socioeconomic status; access to healthcare and support systems in urban areas compared to rural areas, as well as differing definitions of child maltreatment.

Banerjee [47] discussed that child maltreatment was more prevalent in metropolitan cities than in rural areas of India and their study found a high frequency of physical abuse among the slum and pavement dwellers although the severity of maltreatment was low. The study highlighted child labour practices (with 78.4% of children between the ages of 4-5 years working part time jobs) as a result of living in a deprived area of the city. Over half the children who were classed as homeless (i.e. living in drainage pipes, under tarpaulins, flyovers and those living in the open) reported CSA [48]. While child maltreatment figures such as CSA may seem high in urban or slum areas, the disadvantage with rural areas may be that abuse may go under the radar. They may be under-reported or disguised within local traditions such as a dowry culture or child marriages, all behaviours rooted in poverty, and volatile socioeconomic circumstances [49].

Effect of POCSO Act

The Protection of Children from Sexual Offences Act (POCSO Act) was established in 2012 by the Indian government to protect children against offences like sexual abuse. The Act makes it mandatory to report acts of sexual offence and document reporting timelines, prosecution timelines and clearly record all evidence (within a 30-day period). Whilst POSCO has been a relatively recent Act, it's effect on numbers of reported offences has been debated [20]. Whilst the gold standard remains as a multi-discipinary, multi-faceted and holistic team providing psychological and legal support to the child and their families, this level of comprehensive care *under one roof* is yet to exist in India [50,51]. The impact of this systematic study on policymaking will come from its inclusion of the only legislative act in India that focuses on child sexual abuse. This has not been evaluated by the literature to date but was highlighted for future work by Choudhry and colleagues [20].

For peer review only - http://bmjopen.bmj.com/site/about/guidelines.xhtml

Rationale

There are known gaps in the evidence base with regards to the overlap of different forms of child maltreatment and specifically, the gendered effects of child maltreatment. These include the identification and motivation of perpetrators and what happens after maltreatment has happened – a child's awareness of their rights and attitudes towards abuse and neglect and reporting of child maltreatment. The circumstances that encourage reporting and details on protective factors and risk factors of child maltreatment have received limited attention in the literature [20].
This review will build on these thematic and methodological gaps in the literature, synthesise the evidence by gender, urbanisations and POCSO effects and provide practice, policy and clinical solutions that may help reduce the occurrence and impact of child maltreatment in India.

Research Aims and Objectives

The primary research aim is to describe the prevalence of child maltreatment ACEs in India. The secondary aim is to determine the effect of the POCSO Act on child sexual abuse and on other child maltreatment ACEs.

The specific objectives were: to determine the prevalence of child maltreatment ACEs (physical abuse, sexual abuse, emotional abuse and physical and emotional neglect); to present rates by gender, urbanisation (rural, slum or urban) and study sample types (e.g. community, school-going, high-risk such as trafficking victims, etc); to determine any impact of the POCSO Act (by the date of implementation of the Act i.e. 2012) on rates; and, to identify implications for future policy, practice and research amendments.

For peer review only - http://bmjopen.bmj.com/site/about/guidelines.xhtml

Methods and Analysis

Study design
This systematic review and meta-analysis will be carried out in compliance with the Preferred Reporting Items for Systematic Reviews and Meta-Analyses (PRISMA) guidelines [52].

Ethics
A Level 1 self-audit checklist was approved by an ethics panels at the Usher Institute at the University of Edinburgh. Minimal ethical risks are expected. However, Vergnes *et al.,* [53] propose that for SRs particularly on sensitive topics authors should consider a minimal ethical assessment review and report these in their research studies. As a result, in this review, ethical approval details from each included study will be collected.

Participants
Studies with participants aged between 0-18 years will be included in the review; and will have experienced any form of child maltreatment as determined using ISPCAN Child Abuse Screening Tool (ICSAT)-C, the Childhood Trauma Questionnaire, Adverse Childhood Experiences International Questionnaires (ACEIQ) or self-reports or clinician reports. ACEIQ or WHO definitions will also be used to determine child maltreatment [54] in addition to those by Day *et al.,*[55]. The primary outcome measure is any type of child maltreatment: sexual, physical or emotional abuse; and physical and emotional neglect.

Exclusion criteria
Studies that are qualitative in nature or mixed-methods (due to the time factor of this review), non-Indian study contexts, retrospective studies of adult CSA survivors, studies with insufficient published data, media reports, newspaper articles and non-peer reviewed articles.

Outcomes
Defining Childhood Maltreatment
The WHO defines child maltreatment as *'the abuse and neglect that occurs to children under 18 years of age and includes all types of physical and/or emotional ill-treatment, sexual abuse, neglect which results in harm'.* The National Society for the Prevention of Cruelty to Children (NSPCC) defines child maltreatment as harm experienced by a child by an adult or child *that is physical, sexual or emotional but can also involve a lack of love, care and attention* [56]. The NSPCC also set out clear descriptions of what constitutes child maltreatment and specifically each form of abuse and neglect that are summarised here:

Child Physical Abuse (CPA)
Physical abuse happens when a child is hurt or harmed deliberately causing injuries such as bruises, burns, cuts and broken bones. It may also include children being administered medication they do not need or not being given essential medicines for illness. The terms used specifically are kicking, shaking, slapping, throwing, poisoning or drowning. In India, these terms and additional ones such as 'spanking', 'rap' or 'pasting' also have equivalent meanings.

For peer review only

Child Sexual Abuse (CSA)

Sexual abuse happens when a child is forced or tricked into sexual activities. It happens when an abused makes physical contact with a child and includes touching, kissing and oral sex or non-contact abuse which includes exposing or flashing, exposing a child to sex acts or making, viewing or distributing child abuse images or videos.

Child Emotional Abuse (CEA)

Emotional abuse includes acts that humiliate a child by constantly criticising them, insulting or shouting at them, blaming and making them a scapegoat or manipulating a child. These behaviours also include not allowing them to have friends or never saying anything kind or positive or congratulatory to a child. It perpetuates the feelings that a child is not loved, is worthless or inadequate.

Child Physical and Emotional Neglect (CPEN)

Neglect is the on-going failure to meet a child's basic requirements and is often also classed as a form of child abuse. Physical neglect is when a child's basic needs such as food, clothing or shelter are not met or they are not properly supervised or kept safe. Emotional neglect is when a child is not provided with the nurture and stimulation they need to grow and thrive.

Whilst child maltreatment is a global problem, it is certainly difficult to assess and manage in LMICs like India. Specific types of maltreatment such as abandonment, child labour, street-begging and corporal punishment are highly prevalent [57]. Therefore, while generic methods may exist to assess child maltreatment, the value of self-reports from children or from paediatricians, child psychologists or social workers and the police should not be dismissed.

Search Strategy

The relevant search terms to be included in the search strategy will be identified during a preliminary search reported in the study protocol using PubMed's medical subject heading's (MeSH) browser. The search contained both free text terms and controlled vocabulary like MeSH terms as well as truncation and Boolean operators. In order to improve the quality of the search strategy and avoid bias or errors, the strategy includes choosing all appropriate terms, both descriptors and synonyms including those that may be specific to the Indian diaspora [58]. The review used the MeSH terms and keywords documented (Table 1). These were further validated with input from an expert advisory panel in the field of ACEs in India (Appendix 1). Four electronic databases will be systematically searched: PubMed, EMBASE, Cochrane and PsychIndex. PubMed and EMBASE are the two largest healthcare bibliographic databases containing the vast majority of published health-related literature [58,59]. Searching Cochrane and PsychIndex will provide nuanced searches of other systematic reviews of child maltreatment or ACEs from the Asian continent and specifically from India. Systematic reviews will be included in the search to identify potentially relevant studies that may have been excluded by the electronic search strategy. Appendix 2a-2d contains the search strategies used in the four databases respectively.

For peer review only - http://bmjopen.bmj.com/site/about/guidelines.xhtml

**Sexual Abuse**	**Sexual Abuse (cont)**	**Physical Abuse**	**Emotional Abuse**	**Emotional Neglect**	**Additional Child Maltreatment ACEs**
Child* sexual abuse	Child abuse survivor	Child Hitting	Child worthless	Denial clothing	Child neglect
Child sexual assault	Child sexual offender	Child Shaking	Child unloved	Denial shelter	Child psychological assault
Child sexual coercion	Child pornography	Child Throwing	Child inadequate	Denial food	Child denial
Child rape	Child molestation	Child Poisoning	Child failure	Carer failure	Child pasting
Child Prostitution	Child sexual crime	Child Burning	Child unloved	Parental failure	Child emotional assault
Child Crime victims	Child physical	Child Scalding	Child corruption	Emotional harm	
Child Incest	Child assault	Child Drowning	Child unheard	Emotional danger	
Child Perpetrator / Child victim 3	Child beating	Child Suffocating	Cruelty	Child* neglect	
Child Physical violence	Child emo* abuse			Abandonment	
Child Mental Violence	Child maltreat*			Bereavement	
Child Sexual Violence	Child sexual coercion			Grief	
Child sexual offence	Child sexual aggression				

Table 1. Search term strategies using keywords and MeSH terms ("child abuse, sexual"[MeSH Terms] OR ("child"[All Fields] AND "abuse"[All Fields] AND "sexual"[All Fields]) OR "sexual child abuse"[All Fields] OR "physical child abuse" [All Fields] OR "emotional child abuse"[All Fields] OR ("child"[All Fields] AND "sexual"[All Fields] AND "abuse"[All Fields]) OR "child sexual abuse"[All Fields]) AND ("india"[MeSH Terms] OR "india"[All Fields]) - expanded from [20, 55]) and updated to include child maltreatment ACEs and **alternative search terms to include adolescent sexual abuse, adolescent physical assault, etc.*

Language

Only quantitative studies published in the English Language will be included.

Data Management

The selection of studies will be carried out systematically by two people independently, and in accordance with PRISMA guidelines, to improve accuracy and to minimise bias [52]. The first reviewer (first author) is an epidemiologist with expertise in early childhood adversities and the impact on health outcomes; the secondary reviewer is a clinical psychologist with expertise on ACEs in India whilst the third and fourth reviewers, are clinical doctors with expertise in general medicine and paediatric care, validated the selection of studies pre-and post-2012 threshold.

All studies identified from the individual search engines will be imported into a reference manager, EndNote, which will be used for de-duplication. The references will then be extracted into Rayyan for management between the first author and secondary reviewers [60]. The details of all articles processed (including duplicates and details of excluded texts at each screening stage) as well as reasons for inclusion and exclusion from the study will be recorded and logged for the first author and secondary reviewers and presented in a PRISMA flowchart.

For peer review only - http://bmjopen.bmj.com/site/about/guidelines.xhtml

Data Extraction

The data that will be extracted from the included studies are presented in the table (Table 2). An example of a completed data extraction form is presented in this table.

Table 2. Example of a data extraction form [61]

Title of Study<br><br>Authors<br><br>Year of Study	Prevalence of child abuse in Kerala, India: An ICAST-CH based survey<br><br>Manoj Therayil Kumar, Nilamadhab Kar, Sebind Kumar<br><br>2019s
Study Design	Cross-sectional survey in Thrissur, Kerala
Recruitment Strategy	There are 39 schools in Thrissur with 15,150 students in attendance. Random number generation in excel was used to select half the schools on the list and all students in grades 8-10 who attended school on that day were eligible to take part.
Sample setting (school/college, clinical, community), Sample size and response rate	School setting.<br>6957 participants took part in the survey. No details of response rate were provided however the authors state that no questionnaire was returned completely blank.
Child maltreatment definition used (timeframe defined)	All abuse-related questions in the ICAST-CH were scored on a severity scale and categorised into both one year prevalence and lifetime prevalence.
Classification tool<br><br>Validity/Reliability of tool	The International Society for the Prevention of Chlld Abuse and Neglect (IPSCAN) Child Abuse Screening Tool – Child Home Version (ICAST-CH) was used.<br><br>Developed in unison with the WHO and UNICEF and has good criterion validity and was translated into Malayalam and was piloted for use before being administered to the students. Internal consistency measures using Cronbach's alpha for subscales – exposure to violence, physical abuse, emotional abuse, neglect and sexual abuse.
Urban/Rural/Slum-dwelling participants breakdown	Not mentioned
Mean Age (SD)	13.9 years (1.12) for males and 13.7 years (0.99) for females
Gender distribution	2071 males (30.1%)<br>4810 females (69.9%)
Example: CSA prevalence by gender	30.9% in males for one year prevalence<br>10.8% in females for one year prevalence<br><br>34.2% in males for lifetime prevalence<br>14% in females for lifetime prevalence

For peer review only

Statistical Methods used	Frequency and counts used to determine prevalence and presented with 95% confidence intervals<br>Cronbach alpha used to determine internal consistency of the Malayalam version of the scale<br>Significance was set at the 0.05 level as a maximum but 0.01 and <0.001 was reported.
Confounders measured and/or adjusted for	Measured confounders – gender, age, religion, family type, accommodation, parental accommodation and socioeconomic status.
Other confounders discussed but not measured	Literacy rates, cultural strata of participants
Participants experience of:	
Emotional abuse (rate, %)	69.4% in F, 78.2% in M (one year); 70.8% in F, 79.5% in M (lifetime)
Physical Abuse (rate, %)	69.8% in F, 73.1% in M (one year); 73% in F, 76.1% in M (lifetime)
Sexual Abuse (rate, %)	10.8% in F, 30.9% in M (one year); 14% in F, 34.2% in M (lifetime)
Emotional/Physical Neglect (rate, %)	65.1% in F, 66% in M (one year); 65.9% in F, 69.3% in M (lifetime)
Conclusion/Comments	The abuse reported were more occasional experiences rather than frequent reporting nonetheless as 89.9% of any abuse in the past one year and 91% in the lifetime. First study in Indian diaspora to use the ICAST-CH tool to estimate household abuse. Did not include external environments such as schools.
Conflicts of Interest	None declared
Ethical Approval	Ethical approval sought from local authority and educational ministry to conduct the research.
Funders	Municipal Corporation of Thrissur city

Quality Assessment

Risk of bias in individual studies

All abstracts and full text articles will be reviewed independently by the first author and secondary reviewers as a fixed percentage of the work (40%). In order to increase the robustness of the study design, a second reviewer will independently review, and quality assess 20% of all abstracts and full text articles and any discrepancies will be discussed with a third and fourth reviewer or discussed with the wider expert advisor group. The quality of the data (largely cross-sectional and case-control) will be assessed using the Newcastle-Ottawa Quality Assessment Scale (NOS) as it considers key areas: selection (representativeness of the sample, sample size, non-responders and exposure details); comparability (is confounding considered) and, outcome (blinding, recording and statistical test used) [62, 63]. Whilst there are a myriad range of assessment tools available, the NOS has been endorsed by the

For peer review only - http://bmjopen.bmj.com/site/about/guidelines.xhtml

Cochrane Collaboration to assess quality of research studies [59]. An example of a NOS assessment of a sample study is presented in Appendix 3.

Data synthesis

The study will include a narrative synthesis of the findings and attempt a meta-analysis dependant on heterogeneity. A meta-analytic approach will be used including any random-effect pooled prevalence of child maltreatment stratified by gender, urbanisation and pre-and post-POCSO Act. Heterogeneity will be evaluated using forest plots and measuring the impact of the I^2 statistic (I^2 >50% indicates significant heterogeneity) and tested with the χ^2 statistic ($p<0.05$) [64]. We will also report tau^2 values to indicate any between-study variance. We will use a fixed effects model for meta-analysis, except where we identify statistical heterogeneity in which case, we would use a random effects Poisson model [65] to determine an 'average prevalence' across the population of all included studies. We will use the 'metaprop' command to perform meta-analyses of proportions of child maltreatment in STATA, which builds on the 'metan' command [66] but is typically used to pool effects such as proportions from cross-sectional studies instead of odds ratios from case-control studies.

Meta-bia(s)

Research papers written originally in Hindi or any other Indian dialect, that would otherwise be included in the data capture, will not be used for the purpose of this study.

peer review only

For peer review only - http://bmjopen.bmj.com/site/about/guidelines.xhtml

Discussion

The findings of this study will aim to add to the limited knowledge of child maltreatment in India and specifically, the impact of gender, urbanisation and the POCSO Act on the prevalence and determinants of child maltreatment. There are known gaps in the evidence base with regards to the overlap of different forms of child maltreatment and specifically, the gendered effects of child maltreatment. These include the identification and motivation of perpetrators and what happens after maltreatment has happened – a child's awareness of their rights and attitudes towards abuse, neglect and reporting of child maltreatment. The circumstances that encourage reporting, details on protective factors and risk factors of child maltreatment have received limited attention in the literature [20].

Furthermore, the impact of child maltreatment and where they occur (e.g. urban areas versus slum areas) is important as these may offer an insight into potential interventional pathways that differ by geography e.g. enforcing child welfare policies in rural areas [20]. Furthermore, the impact of this study on policymaking will come from its inclusion of the POSCO Act and its effect on numbers of reported offences in research studies.

Implications on Policy and Research

When the evidence on child maltreatment in India is collated and presented, any policy or practice impacts can be tailor-made based on the country-specific context and findings. In India, the challenge will come from addressing cultural norms and traditional values (e.g. gender inequity and reducing child marriages), creating safer environments for children within homes and schools (e.g. authorities penalising assault) and scaling up response and support services for children in different settings such as tribal regions, slum areas, rural communities (e.g. trauma-informed care). This approach has worked in higher-income countries but in LMIC's like India, there are challenges. These include low levels of trauma-informed care for injured children in hospital settings with 91% of healthcare workers not receiving any trauma-informed training [67]. Other challenges included overburdened judicial and criminal systems; poor child welfare services; gender inequality and illiteracy [20]. This study will capture data on health inequities and social determinants that predispose to maltreatment in different geographical areas of India including confounders such as poverty and neglect.

Strengths and Limitations of this study

There are some strengths to our approaches. This is the first systematic review undertaken focussing on all aspects of child maltreatment in India where previous review articles and studies have focussed on a specific facet of maltreatment, e.g. child sexual abuse. Secondly, the inclusion of the POCSO Act makes this review nuanced to comment on any effect of legislation where it has never been formally evaluated [68]. This study will include an analysis of child maltreatment studies and corresponding prevalence rates, both pre-and post-2012. With the inclusion of the POCSO 2012 threshold, it is expected that there will be an increased number of cases reporting all forms of child maltreatment. Thirdly, the authors will attempt to meta-analyse the data depending on heterogeneity scores generated. Choudhry's work highlighted the significant heterogeneity of studies conducted to date and this might limit the quality of meta-analytics possible given the discrepancies in samples size.

For peer review only - http://bmjopen.bmj.com/site/about/guidelines.xhtml

Possible reasons for heterogeneity include varying age ranges of participants, selection bias with sample provenance (e.g. recruitment from observation homes vs. school children), varying urbanisation metrics (e.g. samples including children from all types of socioeconomic backgrounds) and variation in outcome assessment definition (e.g. self-report vs. validated questionnaire).

There are limitations to this review. Firstly, the timeline was limited and as a result, only papers published in English were included although a few studies in Hindi or local dialects may exist. Secondly, excluding grey literature from OPEN Grey, narrative reports from non-governmental organisations or World Bank reports would limit some of the 'unmeasured' exposures and confounders that may increase child maltreatment [39,69](Seth 2015; Subramaniyan *et al.,* 2017). The exclusion of national statistics (e.g. NCRB records) might limit interpretation of any effect of the POCSO Act on conviction rates for child maltreatment. Qualitative studies were also excluded from this review which may not effect prevalence rates of child maltreatment but would allow exploration of non-responses from children (interpretative) or indeed, highlight determinants that have not been identified in quantitative or aggregated reviews [70-72] (Noblit and Hare 1988; Hannes and Lockwood 2012; Seers 2015).

Conclusion

This review will build on these thematic and methodological gaps in the literature, synthesise the evidence by gender, urbanisations and POCSO effects and provide practice, policy and clinical solutions that may help reduce the occurrence and impact of child maltreatment in India.

For peer review only

References

1. WORLD HEALTH ORGANISATION, 2019. Children: reducing mortality. Available from: https://www.who.int/news-room/fact-sheets/detail/children-reducing-mortality [Accessed 2nd February 2020]
2. REYNOLDS, A.J., ROLNICK, A.J., TEMPLE, J.A., 2015. Health and Education in Early Childhood: Predictors, Interventions and Policies. Cambridge: Cambridge University Press.
3. WORLD HEALTH ORGANISATION, 2006. Preventing Child Maltreatment: a guide to taking action and generating evidence. Available from: https://apps.who.int/iris/bitstream/handle/10665/43499/9241594365_eng.pdf;jsessionid=555709D037DBAD5DE822D439283A5F1B?sequence=1 [Accessed 1st of February 2020]
4. WORLD HEALTH ORGANISATION 2017. Country Cooperation Strategy at a glance: India. Available from: https://apps.who.int/iris/bitstream/handle/10665/136895/ccsbrief_ind_en.pdf;jsessionid=90BA4E2E08E57D23C4363B8FBDFC85AC?sequence=1 [Accessed 5th March 2020]
5. BLUM, R. W., LI, M. & NARANJO-RIVERA, G. 2019. Measuring Adverse Child Experiences Among Young Adolescents Globally: Relationships With Depressive Symptoms and Violence Perpetration. *Journal of Adolescent Health,* 65**,** 86-93.
6. BELLIS, M. A., HUGHES, K., FORD, K., RAMOS RODRIGUEZ, G., SETHI, D. & PASSMORE, J. 2019. Life course health consequences and associated annual costs of adverse childhood experiences across Europe and North America: a systematic review and meta-analysis. *The Lancet Public Health,* 4**,** e517-e528.
7. DAMODARAN, D. K. & PAUL, V. K. 2017. Patterning/Clustering of Adverse Childhood Experiences (ACEs): The Indian Scenario. *Psychological Studies,* 62**,** 75-84.
8. HUGHES, K., BELLIS, M. A., HARDCASTLE, K. A., SETHI, D., BUTCHART, A., MIKTON, C., JONES, L. & DUNNE, M. P. 2017. The effect of multiple adverse childhood experiences on health: a systematic review and meta-analysis. *The Lancet Public Health,* 2**,** e356-e366.
9. KOWAL, P. & AFSHAR, S. 2015. Health and the Indian caste system. *The Lancet,* 385**,** 415-416.
10. VIRANI, P. 2000. Bitter Chocolate: Child Sexual Abuse in India. New Delhi: Penguin Books.
11. NIJHARA, K., BHATIA, S. & UNNIKRISHNAN, B. 2018. Corporal Punishment in Children and its Implications on Mental Health. *The Indian Journal of Pediatrics,* 85**,** 405-405.
12. Kochanek S.A., and Hardgrave, R.L., 2008. India: Government and Politics in a Developing Nation. Boston: Thomson Higher Education.
13. UNICEF World Report 2015. Available from: https://www.unicef.org/publications/files/UNICEF_Annual_Report_2015_En.pdf [Accessed 17th November 2019]
14. ANDA, R. F., FELITTI, V. J., BREMNER, J. D., WALKER, J. D., WHITFIELD, C., PERRY, B. D., DUBE, S. R. & GILES, W. H. 2006. The enduring effects of abuse and related adverse experiences in childhood. *European Archives of Psychiatry and Clinical Neuroscience,* 256**,** 174-186.
15. PECHTEL, P. & PIZZAGALLI, D. A. 2011. Effects of early life stress on cognitive and affective function: an integrated review of human literature. *Psychopharmacology (Berl),* 214**,** 55-70.
16. DANESE, A. & MCEWEN, B. S. 2012. Adverse childhood experiences, allostasis, allostatic load, and age-related disease. *Physiol Behav,* 106**,** 29-39.

For peer review only - http://bmjopen.bmj.com/site/about/guidelines.xhtml

17. BEHERE, P. B., SATHYANARAYANA RAO, T. S. & MULMULE, A. N. 2013. Sexual abuse in women with special reference to children: Barriers, boundaries and beyond. *Indian journal of psychiatry,* 55**,** 316-319.
18. NATIONAL CRIME RECORDS BUREAU. 2016. Crime in India – Statistics. Available from: http://ncrb.gov.in/StatPublications/CII/CII2016/Press%20release-Crime%20in%20India%202016.pdf [Accessed 14th Feb 2019]
19. STOLTENBORGH, M., VAN IJZENDOORN, M. H., EUSER, E. M. & BAKERMANS-KRANENBURG, M. J. 2011. A global perspective on child sexual abuse: meta-analysis of prevalence around the world. *Child Maltreat,* 16**,** 79-101.
20. CHOUDHRY, V., DAYAL, R., PILLAI, D., KALOKHE, A. S., BEIER, K. & PATEL, V. 2018. Child sexual abuse in India: A systematic review. *PLoS One,* 13**,** e0205086.
21. WORLD HEALTH ORGANISATION, 2016. CHILD MALTREATMENT. Available from: https://www.who.int/news-room/fact-sheets/detail/child-maltreatment [Accessed 16th October 2019]
22. RADHIKA, K., MANJULA, M. & JAISOORYA, T. S. 2018. Ethical gaps in conducting research among adult survivors of child sexual abuse: a review. *Indian J Med Ethics,* 3**,** 186-192.
23. SETH, R., BOSE, V., QAIYUM, Y., CHANDRASHEKHAR, R., KANSAL, S., TANEJA, I. & SETH, T. 2018. Social Determinants of Child Marriage in Rural India. *Ochsner J,* 18**,** 390-394.
24. KUMAR, M. T., KAR, N. & KUMAR, S. 2019. Prevalence of child abuse in Kerala, India: An ICAST-CH based survey. *Child Abuse Negl,* 89**,** 87-98.
25. HAILES, H. P., YU, R., DANESE, A. & FAZEL, S. 2019. Long-term outcomes of childhood sexual abuse: an umbrella review. *The Lancet Psychiatry,* 6**,** 830-839.
26. CARSON, D.K., FOSTER, J.M., CHOWDHURY, A., 2014. A. Sexual Abuse of Children and Youth in India: An Anthropological Perspective. The Oriental Anthropologist, 14(2):343.
27. FELITTI, V. J., ANDA, R. F., NORDENBERG, D., WILLIAMSON, D. F., SPITZ, A. M., EDWARDS, V., KOSS, M. P. & MARKS, J. S. 1998. Relationship of childhood abuse and household dysfunction to many of the leading causes of death in adults. The Adverse Childhood Experiences (ACE) Study. *Am J Prev Med,* 14**,** 245-58.
28. HOUTEPEN, L.C., HERON, J., SUDERMAN, M.J, TILLING, K., HOWE, L.D., 2018. Adverse childhood experiences in the children of the Avon Longitudinal Study of Parents and Children (ALSPAC). *Wellcome Open Res*.;3:106.
29. GILBERT, R., WIDOM, C. S., BROWNE, K., FERGUSSON, D., WEBB, E. & JANSON, S. 2009. Burden and consequences of child maltreatment in high-income countries. *Lancet,* 373**,** 68-81.
30. DAMODARAN, D.K., VARGHESE, P., 2018. The Unveiled Indian Picture of Adverse Childhood Experiences: Socio-Demographic Correlates Among Youth in India. International Journal of Social SCIENCES Review: 6(7),1248-1257.
31. KIBURI, S. K., MOLEBATSI, K., OBONDO, A. & KURIA, M. W. 2018. Adverse childhood experiences among patients with substance use disorders at a referral psychiatric hospital in Kenya. *BMC Psychiatry,* 18**,** 197.
32. SIDEBOTHAM, P.D., 2001. An ecological approach to child abuse: a creative use of scientific models in research and practice. *Child Abuse Review*, 10: 97-112.
33. DAHLBERG, L.L., KRUG, E.G., Violence-a global public health problem. In: Krug E, Dahlberg LL, Mercy JA, Zwi AB, Lozano R, eds. World Report on Violence and Health. Geneva, Switzerland: World Health Organization; 2002:1–56.
34. SIDEBOTHAM P, HERON, J and ALSPAC Study Team., 2006. Child maltreatment in the "children of the nineties": a cohort study of risk factors. Child Abuse and Neglect; 30 (5): 497-522.
35. THOMAS REUTERS, 2018. India: the worlds most dangerous countries for women in 2018. Available from: http://poll2018.trust.org/country/?id=india [Accessed 2nd December 2019]

For peer review only - http://bmjopen.bmj.com/site/about/guidelines.xhtml

36. KACKER L, VARDAN S, KUMAR P, 2007. Study on Child Abuse:Ministry of Women and Child Development, Government of India.
37. MILLER, E., DAS, M., TANCREDI, D. J., MCCAULEY, H. L., VIRATA, M. C. D., NETTIKSIMMONS, J., O'CONNOR, B., GHOSH, S. & VERMA, R. 2013. Evaluation of a Gender-Based Violence Prevention Program for Student Athletes in Mumbai, India. *Journal of Interpersonal Violence,* 29**,** 758-778.
38. MACIONNIS, J, PLUMMER, K, 2012. Sociology: a global introduction. United Kingdom: Harlow.
39. SUBRAMANIYAN, V. K. S., REDDY, P., CHANDRA, G., RAO, C. & RAO, T. S. S. 2017. Silence of male child sexual abuse in India: Qualitative analysis of barriers for seeking psychiatric help in a multidisciplinary unit in a general hospital. *Indian journal of psychiatry,* 59**,** 202-207.
40. DEB, S, 2018. An empirical investigation into child abuse and neglect in India: burden, impact and protective measures. Pondicherry: Springer.
41. FLEDDERJOHANN, J., AGRAWAL, S., VELLAKKAL, S., BASU, S., CAMPBELL, O., DOYLE, P., EBRAHIM, S. & STUCKLER, D, 2014. Do girls have a nutritional disadvantage compared with boys? Statistical models of breastfeeding and food consumption inequalities among Indian siblings. *PLoS One,* 9**,** e107172.
42. AURINO, E. 2017. Do boys eat better than girls in India? Longitudinal evidence on dietary diversity and food consumption disparities among children and adolescents. *Economics & Human Biology,* 25**,** 99-111.
43. KSHATRIYA, G. K. & ACHARYA, S. K. 2016. Gender Disparities in the Prevalence of Undernutrition and the Higher Risk among the Young Women of Indian Tribes. *PloS one,* 11**,** e0158308-e0158308.
44. PILLAI, A., ANDREWS, T. & PATEL, V. 2008. Violence, psychological distress and the risk of suicidal behaviour in young people in India. *International Journal of Epidemiology,* 38**,** 459-469.
45. BALAJI, M., ANDREWS, T., ANDREW, G. & PATEL, V. 2011. The acceptability, feasibility, and effectiveness of a population-based intervention to promote youth health: an exploratory study in Goa, India. *J Adolesc Health,* 48**,** 453-60.
46. JAYA, J. & HINDIN, M. J. 2007. Nonconsensual sexual experiences of adolescents in urban India. *J Adolesc Health,* 40**,** 573.e7-14.
47. BANERJEE, S.R. 2001. Physical Abuse of Street and Slum Children of Kolkata. Indian Pediatrics, 38, 1163-1170.
48. Save the Children, 2015. Available from: https://www.pwc.in/assets/pdfs/publications/urban-child/urban-child-india-report.pdf [Accessed 2nd October 2019]
49. STROCHLIC, N. 2018. India's forgotten child brides. Available from: https://www.nationalgeographic.com/photography/proof/2018/04/child-brides-marriage-shravasti-india-culture/ [Accessed 5th March 2020]
50. SATHYANARAYANA RAO, T. S., NAGPAL, M. & ANDRADE, C. 2013. Sexual coercion: Time to rise to the challenge. *Indian journal of psychiatry,* 55**,** 211-213.
51. HARBISHETTAR, V. & MATH, S. B. 2014. Violence against women in India: comprehensive care for survivors. *Indian J Med Res,* 140**,** 157-9.
52. MOHER, D., LIBERATI, A., TETZLAFF, J. & ALTMAN, D. G. 2009. Preferred reporting items for systematic reviews and meta-analyses: the PRISMA statement. *PLoS Med,* 6**,** e1000097.
53. VERGNES, J. N., MARCHAL-SIXOU, C., NABET, C., MARET, D. & HAMEL, O. 2010.Ethics in systematic reviews. *J Med Ethics,* 36**,** 771-4.
54. WORLD HEALTH ORGANISATION 2018. Adverse Childhood Experiences International Questionnaire. In Adverse Childhood Experiences International Questionnaire (ACE-IQ). Available from: http://www.who.int/violence_injury_prevention/violence/activities/adverse_childhood_experiences [Accessed 1st of February 2019]

55. DAY, C, HIBBERT, P, CADMAN, S, 2008. A literature review into children abused and/or neglected prior to custody. Available from: https://dera.ioe.ac.uk/7997/1/Abused%20prior%20to%20custody.pdf [Accessed 5th March 2020]
56. NSPCC 2017. Definitions and signs of child abuse. Available from: - https://learning.nspcc.org.uk/media/1188/definitions-signs-child-abuse.pdf [Accessed 20th December]
57. SINGHI, P., SAINI, A. G. & MALHI, P. 2013. Child maltreatment in India. *Paediatrics and International Child Health,* 33**,** 292-300.
58. SALVADOR-OLIVÁN, J. A., MARCO-CUENCA, G. & ARQUERO-AVILÉS, R. 2019. Errors in search strategies used in systematic reviews and their effects on information retrieval. *Journal of the Medical Library Association : JMLA,* 107**,** 210-221.
59. HIGGINS JPT, GREEN S, editors. Cochrane Handbook for Systematic Reviews of Interventions, Version 5.1.0. London: The Cochrane Collaboration; 2011. Section 13.5.2.3. Tools for assessing methodological quality or risk of bias in non-randomized studies.
60. OUZZANI, M., HAMMADY, H., FEDOROWICZ, Z, ELMAGARMID, A., 2016. Rayyan-a web and mobile app for systematic reviews. Systematic Reviews: 5:210.
61. KUMAR, M. T., KAR, N. & KUMAR, S. 2019. Prevalence of child abuse in Kerala, India: An ICAST-CH based survey. *Child Abuse Negl,* 89**,** 87-98.
62. WELLS, G.A., SHEA, B., O'CONNELL. D. PETERSON, J., WELCH, V., LOSOS, M., P.T. 2014. The Newcastle-Ottawa Scale (NOS) for assessing the quality of nonrandomised studies in meta-analyses. Available from: http://www.ohri.ca/programs/clinical_epidemiology/oxford.asp [Accessed 1st of Feb 2019]
63. HERZOG, R., ALVAREZ-PASQUIN, M. J., DIAZ, C., DEL BARRIO, J. L., ESTRADA, J. M. & GIL, A. 2013. Are healthcare workers' intentions to vaccinate related to their knowledge, beliefs and attitudes? A systematic review. *BMC Public Health,* 13**,** 154
64. HIGGINS, J.P., THOMPSON, S.G., 2002. Quantifying heterogeneity in a meta-analysis. Stats Med; 21; 1539-1558.
65. DEEKS JJ, HIGGINS JPT, ALTMAN DG (editors). Chapter 10: Analysing data and undertaking meta-analyses. In: Higgins JPT, Thomas J, Chandler J, Cumpston M, Li T, Page MJ, Welch VA (editors). *Cochrane Handbook for Systematic Reviews of Interventions* version 6.0 (updated July 2019). Cochrane, 2019. Available from www.training.cochrane.org/handbook [Accessed 1st of January 2020]
66. NYAGA, V. N., ARBYN, M. & AERTS, M. 2014. Metaprop: a Stata command to perform meta-analysis of binomial data. *Arch Public Health,* 72**,** 39.
67. HOYSTED, C., BABL, F. E., KASSAM-ADAMS, N., LANDOLT, M. A., JOBSON, L., VAN DER WESTHUIZEN, C., CURTIS, S., KHARBANDA, A. B., LYTTLE, M. D., PARRI, N., STANLEY, R. & ALISIC, E. 2018. Knowledge and training in paediatric medical traumatic stress and trauma-informed care among emergency medical professionals in low- and middle-income countries. *European journal of psychotraumatology,* 9**,** 1468703-1468703.
68. SETH, R & SRIVASTAVA, RN. 2017. Child Sexual Abuse: Management and Prevention, and protection of children from sexual offences (POCSO) Act. Indian paediatrics. 54.
69. SETH, R. 2015. Child Abuse and Neglect in India. *The Indian Journal of Pediatrics,* 82**,** 707-714.
70. NOBLIT, G.W, HARE RD, 1988. Meta-ethnography: synthesizing qualitative studies, vol. 11. California: Sage Publications.
71. HANNES, K, AND LOCKWOOD, C., 2012. Synthesising qualitative research: choosing the right approach. John Wiley and Sons.
72. SEERS, K. 2015. Qualitative systematic reviews: their importance for our understanding of research relevant to pain. *Br J Pain,* 9**,** 36-40.

For peer review only - http://bmjopen.bmj.com/site/about/guidelines.xhtml

Figures

Figure 1. World map showing the rights of a child as measured by UNICEF and World Bank data (2015).

Figure 2. Adverse Childhood Experiences (ACEs) including traditional direct ACEs (child maltreatment: physical, sexual and emotional abuse and neglect) and indirect ACEs (Felitti et al., 1998) and further extended by researchers since the original study (Houtepen et al., 2018)

Figure 3. Potential pathways explaining child maltreatment using the socioecological model proposed by Sidebotham and Heron (2006)

For peer review only

For peer review only - http://bmjopen.bmj.com/site/about/guidelines.xhtml

Acknowledgements: The authors would like to acknowledge the time and contribution of Sarah Herring (University of Bristol) for her invaluable help in locating peer-reviewed articles.

Author Statement: GF, EP and RG conceptualised and designed the protocol. GF and MF drafted the initial manuscript and reviewed this. VC, VB, ES and BH defined the concepts and search terms, data extracting process and the methodological appraisal of studies. GF and NV planned the data extraction alongside MF and PD. All authors have approved and contributed to the final written manuscript.

Funding: This research received no specific grant from any funding agency in the public, commercial or not-for-profit sectors. One of the lead authors is supported by the Medical Research Council (Grant Refs: MR/L022206/1 and MR/N000390/1) at Population Health Sciences, as part of Bristol Medical School, University of Bristol.

Competing Interests: None of the authors have any competing interests to declare.

Patient and Public Involvement: It was not appropriate or possible to involve patients or the public in the design, conduct, reporting or dissemination plans of our research. The research team and advisory/expert panel did consist of clinicians, policymakers and research academics who work in the area of early childhood adversity and were able to advise on search terms used; databases selected and dissemination plans for the work on completion.

For peer review only

For peer review only - http://bmjopen.bmj.com/site/about/guidelines.xhtml

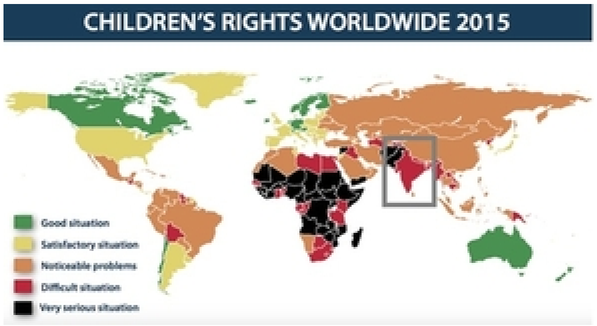

Figure 1. World map showing the rights of a child as measured by UNICEF and World Bank data (2015).

25x13mm (300 x 300 DPI)

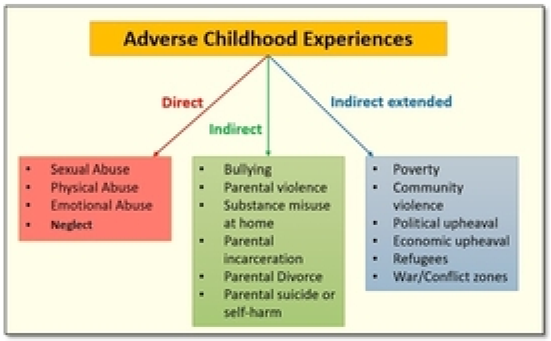

Figure 2. Adverse Childhood Experiences (ACEs) including traditional direct ACEs (child maltreatment: physical, sexual and emotional abuse and neglect) and indirect ACEs (Felitti et al., 1998) and further extended by researchers since the original study (Houtepen et al., 2018)

23x14mm (300 x 300 DPI)

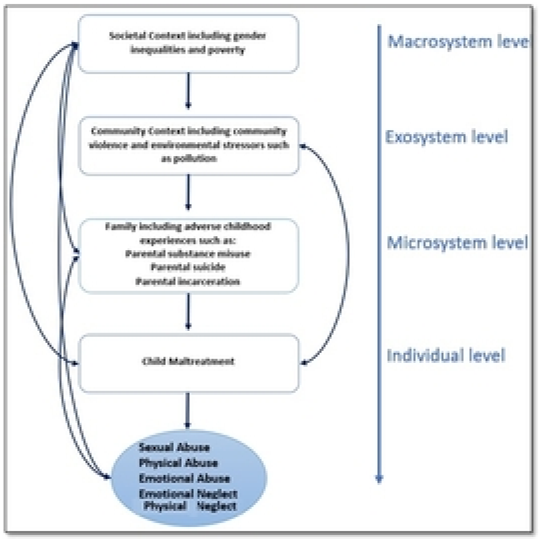

Figure 3. Potential pathways explaining child maltreatment using the socioecological model proposed by Sidebotham and Heron (2006)

22x22mm (300 x 300 DPI)

Appendix 1 – PRISMA Checklist

Section/topic	#	Checklist item	Reported on page #
**TITLE**			
Title	1	Identify the report as a systematic review, meta-analysis, or both.	Cover pg
**ABSTRACT**			
Structured summary	2	Provide a structured summary including, as applicable: background; objectives; data sources; study eligibility criteria, participants, and interventions; study appraisal and synthesis methods; results; limitations; conclusions and implications of key findings; systematic review registration number.	Page 2
**INTRODUCTION**			
Rationale	3	Describe the rationale for the review in the context of what is already known.	Pages 3-6
Objectives	4	Provide an explicit statement of questions being addressed with reference to participants, interventions, comparisons, outcomes, and study design (PICOS).	Pages 9-12
**METHODS**			
Protocol and registration	5	Indicate if a review protocol exists, if and where it can be accessed (e.g., Web address), and, if available, provide registration information including registration number.	Page 2
Eligibility criteria	6	Specify study characteristics (e.g., PICOS, length of follow-up) and report characteristics (e.g., years considered, language, publication status) used as criteria for eligibility, giving rationale.	Pages 8-10
Information sources	7	Describe all information sources (e.g., databases with dates of coverage, contact with study authors to identify additional studies) in the search and date last searched.	Page 10-12
Search	8	Present full electronic search strategy for at least one database, including any limits used, such that it could be repeated.	Page 12; Appendix 2
Study selection	9	State the process for selecting studies (i.e., screening, eligibility, included in systematic review, and, if applicable, included in the meta-analysis).	Page 9-11

For peer review only

Data collection process	10	Describe method of data extraction from reports (e.g., piloted forms, independently, in duplicate) and any processes for obtaining and confirming data from investigators.	Page 12
Data items	11	List and define all variables for which data were sought (e.g., PICOS, funding sources) and any assumptions and simplifications made.	Page 11
Risk of bias in individual studies	12	Describe methods used for assessing risk of bias of individual studies (including specification of whether this was done at the study or outcome level), and how this information is to be used in any data synthesis.	Page 13
Summary measures	13	State the principal summary measures (e.g., risk ratio, difference in means).	Page 9-10
Synthesis of results	14	Describe the methods of handling data and combining results of studies, if done, including measures of consistency (e.g., I^2) for each meta-analysis.	Page 14

Section/topic	#	Checklist item	Reported on page #
Risk of bias across studies	15	Specify any assessment of risk of bias that may affect the cumulative evidence (e.g., publication bias, selective reporting within studies).	Page 14
Additional analyses	16	Describe methods of additional analyses (e.g., sensitivity or subgroup analyses, meta-regression), if done, indicating which were pre-specified.	Page 13-14
**RESULTS**			
Study selection	17	Give numbers of studies screened, assessed for eligibility, and included in the review, with reasons for exclusions at each stage, ideally with a flow diagram.	n/a
Study characteristics	18	For each study, present characteristics for which data were extracted (e.g., study size, PICOS, follow-up period) and provide the citations.	n/a
Risk of bias within studies	19	Present data on risk of bias of each study and, if available, any outcome level assessment (see item 12).	n/a
Results of individual studies	20	For all outcomes considered (benefits or harms), present, for each study: (a) simple summary data for each intervention group (b) effect estimates and confidence intervals, ideally with a forest plot.	n/a
Synthesis of results	21	Present results of each meta-analysis done, including confidence intervals and measures of consistency.	n/a
Risk of bias across studies	22	Present results of any assessment of risk of bias across studies (see Item 15).	n/a
Additional analysis	23	Give results of additional analyses, if done (e.g., sensitivity or subgroup analyses, meta-regression [see Item 16]).	n/a

For peer review only

**DISCUSSION**			
Summary of evidence	24	Summarize the main findings including the strength of evidence for each main outcome; consider their relevance to key groups (e.g., healthcare providers, users, and policy makers).	Pages 15-16
Limitations	25	Discuss limitations at study and outcome level (e.g., risk of bias), and at review-level (e.g., incomplete retrieval of identified research, reporting bias).	Pages 15-16
Conclusions	26	Provide a general interpretation of the results in the context of other evidence, and implications for future research.	Page 16
**FUNDING**			
Funding	27	Describe sources of funding for the systematic review and other support (e.g., supply of data); role of funders for the systematic review.	n/a

From: Moher D, Liberati A, Tetzlaff J, Altman DG, The PRISMA Group (2009). Preferred Reporting Items for Systematic Reviews and Meta-Analyses: The PRISMA Statement. PLoS Med 6(7): e1000097. doi:10.1371/journal.pmed1000097

For more information, visit: **www.prisma-statement.org**.

For peer review only

BMJ Open

The prevalence of child maltreatment in India and its association with gender, urbanisation and policy: a rapid review and meta-analysis protocol

Journal:	*BMJ Open*
Manuscript ID	bmjopen-2020-044983.R1
Article Type:	Protocol
Date Submitted by the Author:	28-Mar-2021
Complete List of Authors:	Fernandes, Gwen; University of Bristol, Population Health Sciences<br>Fernandes, Megan; Norfolk and Norwich University Hospital NHS Trust, Department of Surgery<br>Vaidya , Nilakshi ; National Institute of Mental Health and Neuro Sciences<br>De Souza, Philip; Norfolk and Norwich University Hospital NHS Trust, Department of Medicine<br>Plotnikova, Evgeniya; The University of Edinburgh Usher Institute of Population Health Sciences and Informatics<br>Geddes, Rosemary; The University of Edinburgh Usher Institute of Population Health Sciences and Informatics<br>Holla, Bharath; National Institute of Mental Health and Neurosciences<br>Sharma, Eesha ; National Institute of Mental Health and Neuro Sciences, Child And Adolescent Psychiatry<br>Benegal, Vivek; National Institute of Mental Health and Neurosciences<br>Choudhry , Vikas; Sambodhi Research and Communications Pvt Ltd., Public Health
**Primary Subject Heading**:	Paediatrics
Secondary Subject Heading:	Epidemiology
Keywords:	EPIDEMIOLOGY, Protocols & guidelines < HEALTH SERVICES ADMINISTRATION & MANAGEMENT, MENTAL HEALTH, PUBLIC HEALTH, Child protection < PAEDIATRICS

SCHOLARONE™
Manuscripts

I, the Submitting Author has the right to grant and does grant on behalf of all authors of the Work (as defined in the below author licence), an exclusive licence and/or a non-exclusive licence for contributions from authors who are: i) UK Crown employees; ii) where BMJ has agreed a CC-BY licence shall apply, and/or iii) in accordance with the terms applicable for US Federal Government officers or employees acting as part of their official duties; on a worldwide, perpetual, irrevocable, royalty-free basis to BMJ Publishing Group Ltd ("BMJ") its licensees and where the relevant Journal is co-owned by BMJ to the co-owners of the Journal, to publish the Work in this journal and any other BMJ products and to exploit all rights, as set out in our licence.

The Submitting Author accepts and understands that any supply made under these terms is made by BMJ to the Submitting Author unless you are acting as an employee on behalf of your employer or a postgraduate student of an affiliated institution which is paying any applicable article publishing charge ("APC") for Open Access articles. Where the Submitting Author wishes to make the Work available on an Open Access basis (and intends to pay the relevant APC), the terms of reuse of such Open Access shall be governed by a Creative Commons licence – details of these licences and which Creative Commons licence will apply to this Work are set out in our licence referred to above.

Other than as permitted in any relevant BMJ Author's Self Archiving Policies, I confirm this Work has not been accepted for publication elsewhere, is not being considered for publication elsewhere and does not duplicate material already published. I confirm all authors consent to publication of this Work and authorise the granting of this licence.

For peer review only

Rapid Review Protocol Paper

The prevalence of child maltreatment in India and its association with gender, urbanisation and policy: a rapid review and meta-analysis protocol

Gwen Sascha Fernandes[*1], Megan Fernandes*[*2], Nilakshi Vaidya*[3], Philip De Souza*[4], Evgeniya Plotnikova*[5], Rosemary Geddes*[5], Bharath Holla*[3], Eesha Sharma*[3], Vivek Benegal*[3], Vikas Choudhry*[6]
*denotes joint first authorship

1. Population Health Sciences, Bristol Medical School, University of Bristol, UK
2. Department of General Surgery, Norfolk and Norwich University Hospital, Norwich, UK
3. The National Institute of Mental Health and Neurosciences, Bangalore, India
4. Department of Medicine, Norfolk and Norwich University Hospital, Norwich, UK
5. Usher Institute, School of Medicine, University of Edinburgh, UK
6. Sambodhi Research and Communications Pvt Ltd

Correspondence to:
Dr Gwen Fernandes; gwen.fernandes@bristol.ac.uk
Population Health Sciences
Bristol Medical School,
Barley House
University of Bristol
BS8 2BN

For peer review only

Abstract

Introduction: India is home to 20% of the world's children and yet, little is known on the magnitude and trends of child maltreatment nationwide. The aims of this review are to provide a prevalence of child maltreatment in India with considerations for any effects of gender; urbanisation (e.g. urban versus rural); and, legislation (Protection of Children from Sexual Offences Act 2012).

Methods and Analysis: A rapid review will be undertaken of all quantitative peer-reviewed studies on child maltreatment in India between 2005 and 2020. Four electronic databases will be systematically searched: PubMed, EMBASE, Cochrane and PsychInfo. The primary outcomes will include all aspects of child maltreatment: physical abuse, sexual abuse, emotional abuse, emotional neglect and physical neglect. Study participants will be between 0-18 years and will have reported maltreatment experiences using validated, reliable tools such as the Adverse Childhood Experiences Questionnaire as well as child self-reports and clinician reports. Study selection will follow the Preferred Reporting Items for Systematic Reviews and Meta-Analyses guidelines, and the methodological appraisal of the studies will be assessed by the Newcastle-Ottawa Quality assessment scale. A narrative synthesis will be conducted for all included studies. Also, if sufficient data are available, a meta-analysis will be conducted. Effect sizes will be determined from random-effects models stratified by gender, urbanisation and pre-and post the 2012 POCSO Act cut off. I^2 statistics will be used to assess heterogeneity and identify their potential sources and tau^2 statistics will indicate any between-study variance.

Ethics and Dissemination: As this is a rapid review, minimal ethical risks are expected. The protocol and Level 1 self-audit checklist was submitted and approved by the Usher Research Ethics Group (UREG) panel in the Usher Institute (School of Medicine and Veterinary Sciences) at the University of Edinburgh [Reference B126255]. Findings from this review will be disseminated widely through peer-reviewed publication and in various media, for example, conferences, congresses or symposia.

Registration: A study protocol was developed before starting this review and was registered with the international prospective register of systematic reviews (PROSPERO) (registration number CRD42019150403)

For peer review only

Strengths and Limitations

- This is the first rapid review of the peer-reviewed and published evidence on child maltreatment in India drawing on studies from four electronic databases: PubMed, EMBASE, Cochrane and PsychInfo
- The primary outcome is any type of prospectively recalled child maltreatment including physical abuse and neglect, emotional abuse and neglect and sexual abuse.
- First study to evaluate the effects of the legislative Act pre- and post-2012. (Protection of Children from Sexual Offences Act 2012)
- The review is limited by the exclusion of the grey literature and non-English peer-reviewed publications due to the lack of resources and funding available to conduct the review.
- The review will include a narrative synthesis of non-governmental reports, media articles and other sources of grey literature to inform the findings.

For peer review only - http://bmjopen.bmj.com/site/about/guidelines.xhtml

Introduction

In the past two decades, significant progress has been made in reducing child deaths. UNICEF estimates that, worldwide, there were 12.6 million children who died prematurely before the age of five in 1990 and this has declined to 5.3 million in 2018 [1]. Of the children who do survive, 200 million do not reach their full potential due to a combination of family socioeconomic status, geographic location, ethnicity, disability or religious orientation [1]. It is every child's right to develop and thrive, not just survive. Balanced nutrition, good healthcare and consistent care and encouragement in supportive home and school environments and positive neighbourhood environments enable children to lead healthy lives [2]. The early years of childhood therefore provide the basis of intelligence and learning, personality and self-motivation, social behaviour and collaboration, which extend into adulthood [3].

The World Health Organization (WHO) defines child maltreatment as 'the abuse and neglect that occurs to children under 18 years of age and includes all types of physical and/or emotional ill-treatment, sexual abuse, and neglect which results in harm'. The WHO reports that worldwide, child maltreatment is widespread with 1 in 4 adults reporting being physically abused as children [4,5]. Research suggests that children who have experienced such adversity do not receive the right nutrition, care or opportunities to learn and grow as children in more affluent geographical areas or economies [6,7]. However, these findings are not generalisable to lower-and middle-income countries (LMICs) like India where millions of children may be exposed to other, specific adversities such as conflict, political instability, community violence and other traumatic life experiences [7,8]. Research on early childhood adversity and consequences have been limited in India, including a lack of cross-sectional and longitudinal studies [9]. These adversities such as abuse or neglect, along with associated habits such as underage smoking or drinking, formed in childhood, have been linked to a range of poor physical and mental health outcomes throughout the life course and ultimately greater premature mortality [10]. These adversities and consequent health inequalities can be exacerbated by socioeconomic status and poverty, urbanisation (rural areas or slum areas compared with urban zones) or even caste [11].

In her book, Bitter Chocolate [12], Pinki Virani discusses the context of these adversities in India – a highly patriarchal culture in which reprimanding, punishing or spanking a child (physically abusive behaviour) is a cultural norm [13]. The Indian economy is further characterised by external adversities from a rapidly growing economy, stark urban-rural divides, religious strife and geopolitical upheavals [14]. Data from UNICEF and the World Bank [15] shows a global perspective of child rights and Indian children are marked as experiencing a difficult situation as categorised by their rights, access to education and healthcare and exposure to child labour and child marriages (Figure 1). It is no surprise that physiological and neurobiological studies have found that adversities such as child maltreatment can lead to changes of neural networks, impaired nervous, endocrine and immune systems development, resulting in physical, mental and emotional dysfunction and chronic physiological damage [16-18].

Figure 1. World map showing the rights of a child as measured by UNICEF and World Bank data [15].

For peer review only - http://bmjopen.bmj.com/site/about/guidelines.xhtml

An Indian context

In India, home to 19% of the world's children, it is estimated every second child is exposed to sexual abuse and violence [19]. The Indian National Crimes Records Bureau (NCRB) report a child is sexually abused every 15 minutes and 53% of children report abuse by a parent, relative or schoolteacher [20]. The prevalence of child sexual abuse (CSA) in high-income countries is 20% for women and 8% for men [21] but in India, the estimates vary between 4-66% for women and 4-57% for men [22-24]. These figures are considered underestimates because of limited surveillance of direct childhood adversity such as physical abuse or emotional abuse as well as CSA due to stigma particularly affecting girls; underreporting of cases by healthcare and police authorities; stigma and taboo associated with being a victim or experiencing abuse, and varying prevalence rates by geography (e.g. urban versus rural versus slum settings) as well as varying communities (school-based, hospital based, trafficking victims etc) [24-27].

Poverty, and especially urban poverty in India is prevalent due to high economic migration that has translocated poverty from rural settings to urbanised areas [28]. Whilst the proportion of deprived populations in India dropped from 45% to 22% between 1994 and 2012, one in every six urban Indians live in slums [29]. Health inequalities that exist as a result of poverty and rapid urbanisations need to be reduced so that child maltreatment, violence against girls and women, aims of the United Nations Sustainable Development Goals can be achieved. Bywaters *et al.,* [30] report strong associations between the socioeconomic status of a family and the risk of children within the household experiencing abuse and neglect. The greater economic hardship in areas like urban slums, the greater the likelihood and severity of child maltreatment. By contrast, we see evidence of the reverse in Deb and Modak's [31] study where violence is proportional to economic status as well as cultural beliefs and practices. Their data on socioeconomic status found that children from high income households were almost four times more vulnerable to physical violence compared to children from low-income households. This is attributed to parents within high income households having high expectations of their children especially with regards to academic achievement and a general undermining of a child's status compared to elder perspectives [32]. We see the nuanced effects of economics and urbanisation as children from more deprived backgrounds have parents who remain indifferent to their care and well-being especially for female children [33]. Given these findings, this review will carefully consider socioeconomic status of families, urbanisation metrics and specifically, the types of maltreatment affecting each sex within these settings.

Previous research has demonstrated that experiences of child maltreatment rarely occur in isolation and in fact, have a tendency to cluster [9]. Community-based studies have reported the prevalence and overlap of childhood sexual, physical and emotional abuse, but in India this has been limited. Choudhry's [22] systematic review focussed on one aspect of child maltreatment: CSA, and was seminal work in detailing the high prevalence rates of CSA in Indian children. However, the authors acknowledged that CSA does not occur in silos and often co-occurs with other forms of child maltreatment in the same child [34].

Childhood Adversity in India

For peer review only - http://bmjopen.bmj.com/site/about/guidelines.xhtml

Over the past 20 years, the phrase ‘adverse childhood experiences’ or (ACEs) have been studied as forms of maltreatment and household dysfunction affecting children either directly or indirectly. Feliiti and colleagues [35] found a strong graded relationship between exposure to abuse or dysfunction during childhood and multiple risk factors for mortality in adulthood. ACEs have been associated with non-communicable conditions such as cardiovascular disease, cancer or mental illness [7, 36-38].

Whilst alternative discourse on ACEs exists [39], there are limitations in the application of this ACEs framework including different types of exposures, lack of consideration for frequency or severity of occurrence and the relevance to population level, rather than individual level, policies. Also, the application of this framework within LMICs like India may be limited given the effects other adversities such as poverty, community violence and political strife [40].

Using Felitti’s ACEs framework and extending it with additional adversities [40], Figure 2 depicts different types of adversities each bearing significant ramifications extending from childhood to adulthood and beyond – affecting the family and society as a whole [41]. However, in India, the data on child maltreatment across different different states and settings is available but the data on ACEs is limited. A few recent studies have been able to identify the prevalence of ACEs in specific communities [22]. Damodaran and Paul[9] compiled data on ACEs and specifically, child maltreatment in youth based in Kerala (South India) and reported that in a sample of 600 young people, 91% had reported at least one ACE and over half the population reported three or more ACEs. However, in terms of severity, males reported exposure to more severe child maltreatment than females such as physical and sexual abuse

Figure 2. Adverse Childhood Experiences (ACEs) including traditional direct ACEs (child maltreatment: physical, sexual and emotional abuse and neglect) and indirect ACEs (Felitti et al., 1998) and further extended by researchers since the original study (Houtepen et al., 2018)

In India, lower incomes, poor living conditions and living with domestic violence are adversities that can lead to neglect and abuse and often ‘cluster’ [42]. Figure 3 combines the socioecological model of child maltreatment [43] and suggests potential pathways (e.g. from a macrosystem/societal or exosystem/community level) that may contribute to child maltreatment [[44-45]. Particularly, around the individual level factors – there is a gap in the rates of other types of child maltreatment associated with CSA (e.g. emotional abuse or neglect). Whilst the topic of ACEs in India has received limited attention, child sexual abuse (CSA) has received considerable attention from academics, clinicians, policymakers and legislators in recent years [22,46]. With the high prevalence of all types of child abuse in India, an attempt to amalgamate and compile the findings and present these figures in a systematic, cohesive and coherent manner is pressing [5].

Figure 3. Potential pathways explaining child maltreatment using the socioecological model proposed by Sidebotham and Heron [45]

For peer review only - http://bmjopen.bmj.com/site/about/guidelines.xhtml

Effect of Gender

Previous research has suggested the nuanced effect of gender on the occurrence of child maltreatment in India [22]. In India, gender identification is illegal and yet, almost half of girls 'wish they were born boys' [47]. Although anecdotal evidence and case reports suggest that prevalence of sexual abuse is higher in female children than in male children, based on hospital services or care services utilisation [48], some studies shows equal rates of sexual abuse across gender [47,49]. Deb and colleagues [50] suggest that boys may be experiencing and reporting more sexual abuse whilst girls are less likely to be aware of abuse (knowledge and attitudes) or less likely to report abuse for fear of shame or stigma. Society is more dismissive of male abuse than that of girls as patriarchal tendencies to 'protect' include girls, but do not extend to boys [49]. However, when it comes to neglect with regards to food and education security, girls are known to be disadvantaged [51]. India is the only country where girls have greater rates of under-five mortality compared with boys. Indian girls are breastfed for shorter periods and consume less milk (ibid). The reasons for this discrepancy are varied: there is a preference for sons over daughters in India and as an extension, more opportunities with regards to food, healthcare and education are afforded to male children [52]. However, given the complexities of gender effects in different types of abuse, generalisations cannot be drawn due to the limited number of studies, methodological limitations (e.g. small sample sizes, non-generalisability of findings to the wider Indian diaspora), urbanisation and socioeconomic privilege (e.g. remote tribal areas being worse affected than wealthier urban ones [53], and reporting bias (e.g. particularly if parents reported via food diaries what was consumed).

Effect of Urbanisation

The prevalence of child maltreatment by urbanisation (rural, slum or urban areas) has received limited attention in the evidence base. Only two studies [54,55] included community populations from rural areas as well as urban areas. Whilst other cross-sectional studies have been conducted in urban areas of India [56], the child maltreatment prevalence figures (32% of boys and 42% of girls) are higher but not comparable due to differences in sample size; socioeconomic status; access to healthcare and support systems in urban areas compared to rural areas, as well as differing definitions of child maltreatment.

Banerjee [57] discussed that child maltreatment was more prevalent in metropolitan cities than in rural areas of India and their study found a high frequency of physical abuse among the slum and pavement dwellers although the severity of maltreatment was low. The study highlighted child labour practices (with 78.4% of children between the ages of 4-5 years working part time jobs) as a result of living in a deprived area of the city. This study also highlighted the fact that often child maltreatment is a product of dire economic distress and within this context; small-scale regional or local studies might help understand the prevalence of child maltreatment in specific areas by urbanisation metrics. Over half the children who were classed as homeless (i.e. living in drainage pipes, under tarpaulins, flyovers and those living in the open) reported CSA [58]. While child maltreatment figures such as CSA may seem high in urban or slum areas, the disadvantage with rural areas may be that abuse may go under the radar. They may be under-reported or disguised within local traditions such as a dowry culture or child marriages, all behaviours rooted in poverty, and volatile socioeconomic circumstances [59].

For peer review only - http://bmjopen.bmj.com/site/about/guidelines.xhtml

Effect of POCSO Act

The Protection of Children from Sexual Offences Act (POCSO Act) was established in 2012 by the Indian government to protect children against offences like sexual abuse. The Act makes it mandatory to report acts of sexual offence and document reporting timelines, prosecution timelines and clearly record all evidence (within a 30-day period). Whilst POSCO has been a relatively recent Act, it's effect on numbers of reported offences has been debated [22]. Whilst the gold standard remains as a multi-discipinary, multi-faceted and holistic team providing psychological and legal support to the child and their families, this level of comprehensive care *under one roof* is yet to exist in India [60.61]. The impact of this study on policymaking will come from its inclusion of the only legislative act in India that focuses on child sexual abuse. Given the overlap between different types of abuse and neglect and their tendency to 'cluster', we will determine any effects of POCSO pre- and post-2012 on all forms of child maltreatment. This has not been evaluated by the literature to date but was highlighted for future work by Choudhry and colleagues [22].

Rationale

There are known gaps in the evidence base with regards to the overlap of different forms of child maltreatment and specifically, the gendered effects of child maltreatment. These include the identification and motivation of perpetrators and what happens after maltreatment has happened – a child's awareness of their rights and attitudes towards abuse and neglect and reporting of child maltreatment. The circumstances that encourage reporting and details on protective factors and risk factors of child maltreatment have received limited attention in the literature [22].
This review will build on these thematic and methodological gaps in the literature, synthesise the evidence by gender, urbanisations and POCSO effects and provide practice, policy and clinical solutions that may help reduce the occurrence and impact of child maltreatment in India.

Research Aims and Objectives

The primary research aim is to describe the prevalence of child maltreatment in India. The secondary aim is to determine the effect of the POCSO Act on child sexual abuse and on other forms of child maltreatment .

The specific objectives were: to determine the prevalence of child maltreatment (physical abuse, sexual abuse, emotional abuse and physical and emotional neglect); to present rates by gender, urbanisation (rural, slum or urban) and study sample types (e.g. community, school-going, high-risk such as trafficking victims, etc); to determine any impact of the POCSO Act (by the date of implementation of the Act i.e. 2012) on rates; and, to identify implications for future policy, practice and research amendments.

For peer review only - http://bmjopen.bmj.com/site/about/guidelines.xhtml

Methods and Analysis

Study design

This review and meta-analysis will be carried out in compliance with the Cochrane Handbook for Systematic Reviews of Interventions (version 6.1) and reported in compliance with the Preferred Reporting Items for Systematic Reviews and Meta-Analyses (PRISMA) guidelines [62,63].

Ethics

A Level 1 self-audit checklist was approved by an ethics panels at the Usher Institute at the University of Edinburgh. Minimal ethical risks are expected. However, Vergnes *et al.,* [64] propose that for SRs particularly on sensitive topics authors should consider a minimal ethical assessment review and report these in their research studies. As a result, in this review, ethical approval details from each included study will be collected.

Patient and Public Involvement

It was not appropriate or possible to involve patients or the public in the design, conduct, reporting or dissemination plans of our research. The research team and advisory/expert panel consisted of clinicians, policymakers and research academics who work in the area of early childhood adversity and were able to advise on search terms used; databases selected and dissemination plans for the work on completion.

Participants

Inclusion criteria

Studies between 1st January 2005 to 1st January 2020 will be included to allow a comparison of an 8-year window pre- and post- 2012 POCSO Act. Studies with participants aged between 0-18 years will be included in the review; and will have experienced any form of child maltreatment as determined using ISPCAN Child Abuse Screening Tool (ICSAT)-C, the Childhood Trauma Questionnaire, Adverse Childhood Experiences International Questionnaires (ACEIQ) or self-reports or clinician reports. ACEIQ or WHO definitions will also be used to determine child maltreatment [65] in addition to those by Day *et al.,* [66]. The primary outcome measure is any type of child maltreatment: sexual, physical or emotional abuse; and physical and emotional neglect. In addition to the definition, the time frame of recall, prospective recall in our case, warrants discussion.

Often, prospective and retrospective measures of child maltreatment do not always identify the same individuals [67]. Retrospective studies of child maltreatment are often regarded as a shortcut as significant time; finance and resources are not required to undertake prospective studies [68]. There is low agreement between prospective and retrospective assessments of child maltreatment that can be explained by factors such as motivation of reporting, measurement features and memory bias. Firstly, motivation can reduce agreement if individuals may gain something by intentionally withholding data about their maltreatment for embarrassment or fear or feeling uncomfortable or upset. Secondly, child maltreatment assessments are known to have imperfect test-retest reliability [69] and prospective measures might only account for the more severe of cases whilst retrospective 'true positive' case [67]. Lastly, memory bias can reduce agreement as a result of impaired brain development [70], deficit in memory consolidation of traumatic events or disrupted sleep patterns [71]. Therefore, given the variation, the focus on prospective recall (rather than retrospective) of child

For peer review only - http://bmjopen.bmj.com/site/about/guidelines.xhtml

maltreatment for systematically reviewing the literature on abuse and neglect in children would reduce variation errors, measurement bias and memory bias [67].

Exclusion criteria
Studies that are qualitative in nature or mixed-methods (due to the time factor of this review), non-Indian study contexts, non-English studies, retrospective studies of adult CSA survivors, studies with insufficient peer-reviewed published data, media reports, newspaper articles and non-peer reviewed articles will be excluded from this review. Only quantitative studies published in the English Language will be included.

Outcomes

Defining Childhood Maltreatment
The WHO defines child maltreatment as *'the abuse and neglect that occurs to children under 18 years of age and includes all types of physical and/or emotional ill-treatment, sexual abuse, neglect which results in harm'.* The National Society for the Prevention of Cruelty to Children (NSPCC) defines child maltreatment as harm experienced by a child by an adult or child *that is physical, sexual or emotional but can also involve a lack of love, care and attention* [72]. The NSPCC also set out clear descriptions of what constitutes child maltreatment and specifically each form of abuse and neglect that are summarised here:

Child Physical Abuse (CPA)
Physical abuse happens when a child is hurt or harmed deliberately causing injuries such as bruises, burns, cuts and broken bones. It may also include children being administered medication they do not need or not being given essential medicines for illness. The terms used specifically are kicking, shaking, slapping, throwing, poisoning or drowning. In India, these terms and additional ones such as 'spanking', 'rap' or 'pasting' also have equivalent meanings.

Child Sexual Abuse (CSA)
Sexual abuse happens when a child is forced or tricked into sexual activities. It happens when an abused makes physical contact with a child and includes touching, kissing and oral sex or non-contact abuse which includes exposing or flashing, exposing a child to sex acts or making, viewing or distributing child abuse images or videos.

Child Emotional Abuse (CEA)
Emotional abuse includes acts that humiliate a child by constantly criticising them, insulting or shouting at them, blaming and making them a scapegoat or manipulating a child. These behaviours also include not allowing them to have friends or never saying anything kind or positive or congratulatory to a child. It perpetuates the feelings that a child is not loved, is worthless or inadequate.

Child Physical and Emotional Neglect (CPEN)
Neglect is the on-going failure to meet a child's basic requirements and is often also classed as a form of child abuse. Physical neglect is when a child's basic needs such as food, clothing or shelter are not met or they are not properly supervised or kept safe. Emotional neglect is when a child is not provided with the nurture and stimulation they need to grow and thrive.

For peer review only - http://bmjopen.bmj.com/site/about/guidelines.xhtml

Whilst child maltreatment is a global problem, it is certainly difficult to assess and manage in LMICs like India. Specific types of maltreatment such as abandonment, child labour, street-begging and corporal punishment are highly prevalent [73]. Therefore, while generic methods may exist to assess child maltreatment, the value of self-reports from children or from paediatricians, child psychologists or social workers and the police should not be dismissed.

Search Strategy

The relevant search terms to be included in the search strategy will be identified during a preliminary search reported in the study protocol using PubMed's medical subject heading's (MeSH) browser. The search contained both free text terms and controlled vocabulary like MeSH terms as well as truncation and Boolean operators. In order to improve the quality of the search strategy and avoid bias or errors, the strategy includes choosing all appropriate terms, both descriptors and synonyms including those that may be specific to the Indian diaspora . Firstly, the initial search terms were developed with reference to seminal works or major publications of child maltreatment and early childhood adversity in India [9, 26, 47,74]. The review then used the MeSH terms and keywords documented (Table 1) and were further enhanced by the search strategy from a systematic review conducted on child sexual abuse in Indian children [22]. These were further validated with input from an expert advisory panel in the field of systematic reviews and ACEs in India (Appendix 1). Five electronic databases will be systematically searched: PubMed, EMBASE, Cochrane and PsychInfo and Campbell Library. PubMed and EMBASE are the two largest healthcare bibliographic databases containing the vast majority of published health-related literature [75,76]. Searching Cochrane and PsychInfo will provide nuanced searches of other systematic reviews of child maltreatment or ACEs from the Asian continent and specifically from India. Systematic reviews will be included in the search to identify potentially relevant studies that may have been excluded by the electronic search strategy. Appendix 2a-2d contains the search strategies used in the four databases respectively. These search strategies and advice on specific electronic databases will be cross-checked and verified by an information specialist. The final search strategy used will be published in detail in the future published manuscript of results.

**Sexual Abuse**	**Sexual Abuse (cont)**	**Physical Abuse**	**Emotional Abuse**	**Emotional Neglect**	**Additional Child Maltreatment definitions**
Child* sexual abuse	Child abuse survivor	Child Hitting	Child worthless	Denial clothing	Child neglect
Child sexual assault	Child sexual offender	Child Shaking	Child unloved	Denial shelter	Child psychological assault
Child sexual coercion	Child pornography	Child Throwing	Child inadequate	Denial food	Child denial
Child rape	Child molestation	Child Poisoning	Child failure	Carer failure	Child pasting
Child Prostitution	Child sexual crime	Child Burning	Child unloved	Parental failure	Child emotional assault
Child Crime victims	Child physical	Child Scalding	Child corruption	Emotional harm	Non-accidental injury

For peer review only - http://bmjopen.bmj.com/site/about/guidelines.xhtml

Child Incest	Child assault	Child Drowning	Child unheard	Emotional danger	
Child Perpetrator / Child victim 3	Child beating	Child Suffocating	Cruelty	Child* neglect	
Child Physical violence	Child emo* abuse			Abandonment	
Child Mental Violence	Child maltreat*			Bereavement	
Child Sexual Violence	Child sexual coercion			Grief	
Child sexual offence	Child sexual aggression				

Table 1. Search term strategies using keywords and MeSH terms ("child abuse, sexual"[MeSH Terms] OR ("child"[All Fields] AND "abuse"[All Fields] AND "sexual"[All Fields]) OR "sexual child abuse"[All Fields] OR "physical child abuse" [All Fields] OR "emotional child abuse"[All Fields] OR ("child"[All Fields] AND "sexual"[All Fields] AND "abuse"[All Fields]) OR "child sexual abuse"[All Fields]) AND ("india"[MeSH Terms] OR "india"[All Fields]) - expanded from Choudhry *et al.,* (2018) and Day *et al.,* 2008 and updated to include child maltreatment definitions and **alternative search terms to include adolescent sexual abuse, adolescent physical assault, etc.*

For peer review only

Data Management

The selection of studies will be carried out systematically by two people independently, and in accordance with PRISMA guidelines, to improve accuracy and to minimise bias [63]. The first reviewer (first author) is an epidemiologist with expertise in early childhood adversities and the impact on health outcomes; the secondary reviewer is a clinical psychologist with expertise on ACEs in India whilst the third and fourth reviewers, are clinical doctors with expertise in general medicine and paediatric care, validated the selection of studies pre-and post-2012 threshold.

All studies identified from the individual search engines will be imported into a reference manager, EndNote, which will be used for de-duplication. The references will then be extracted into Rayyan for management between the first author and secondary reviewers [77]. All abstracts and full text articles will be reviewed independently by the first author and secondary reviewers as a fixed percentage of the work (40%). The details of all articles processed (including duplicates and details of excluded texts at each screening stage) as well as reasons for inclusion and exclusion from the study will be recorded and logged for the first author and secondary reviewers and presented in a PRISMA flowchart. At the title/abstract screening stage, the authors will use Rayyan's coding system (traffic light system) with appropriate tags such as 'wrong outcome of interest' in order to code each study (done independently by the authors on Rayyan's blinded review setting). Once all titles/abstracts are screened, we will unblind all authors and discuss those titles/abstract to be included, or excluded or further discussed with the expert panel as a result of discrepancies [77].

All selected full-text articles will be screened by the primary author and secondary reviewers and the reasons for exclusion of these articles will be recorded and presented in the final manuscript.

Data Extraction

The following data will be extracted from the included studies: title; author information; year of study (not publication date); study design; recruitment strategy and sample characteristics (school-based, community, hospital based, etc); sample size; urbanisation metrics (urban or rural or slum); response rate; definition of child maltreatment used; timeframe of child maltreatment (in years e.g. past one year or

For peer review only - http://bmjopen.bmj.com/site/about/guidelines.xhtml

past 6 months or half a year); mean age of participants; gender distribution; statistical methodology; confounder data including parental relationships, home ownership, family settings (e.g. nuclear, joint, mixed), religion, educational status and literacy rates; prevalence rate of each type of child maltreatment; author's key message or conclusions; ethical approval and any conflicts of interest. An example of a completed data extraction form is presented in the following table (Table 2).

Title of Study<br><br>Authors<br><br>Year of Study	Prevalence of child abuse in Kerala, India: An ICAST-CH based survey<br><br>Manoj Therayil Kumar, Nilamadhab Kar, Sebind Kumar<br><br>2019s
Study Design	Cross-sectional survey in Thrissur, Kerala
Recruitment Strategy	There are 39 schools in Thrissur with 15,150 students in attendance. Random number generation in excel was used to select half the schools on the list and all students in grades 8-10 who attended school on that day were eligible to take part.
Sample setting (school/college, clinical, community), Sample size and response rate	School setting.<br>6957 participants took part in the survey. No details of response rate were provided however the authors state that no questionnaire was returned completely blank.
Child maltreatment definition used (timeframe defined)	All abuse-related questions in the ICAST-CH were scored on a severity scale and categorised into both one year prevalence and lifetime prevalence.
Classification tool<br><br>Validity/Reliability of tool	The International Society for the Prevention of Chlld Abuse and Neglect (IPSCAN) Child Abuse Screening Tool – Child Home Version (ICAST-CH) was used.<br><br>Developed in unison with the WHO and UNICEF and has good criterion validity and was translated into Malayalam and was piloted for use before being administered to the students. Internal consistency measures using Cronbach's alpha for subscales – exposure to violence, physical abuse, emotional abuse, neglect and sexual abuse.
Urban/Rural/Slum-dwelling participants breakdown	Not mentioned
Mean Age (SD)	13.9 years (1.12) for males and 13.7 years (0.99) for females
Gender distribution	2071 males (30.1%)<br>4810 females (69.9%)
Example: CSA prevalence by gender	30.9% in males for one year prevalence<br>10.8% in females for one year prevalence

For peer review only - http://bmjopen.bmj.com/site/about/guidelines.xhtml

	34.2% in males for lifetime prevalence<br>14% in females for lifetime prevalence
Statistical Methods used	Frequency and counts used to determine prevalence and presented with 95% confidence intervals<br>Cronbach alpha used to determine internal consistency of the Malayalam version of the scale<br>Significance was set at the 0.05 level as a maximum but 0.01 and <0.001 was reported.
Confounders measured and/or adjusted for	Measured confounders – gender, age, religion, family type, accommodation, parental accommodation and socioeconomic status.
Other confounders discussed but not measured	Literacy rates, cultural strata of participants
Participants experience of:<br><br>Emotional abuse (rate, %)<br>Physical Abuse (rate, %)<br>Sexual Abuse (rate, %)<br>Emotional Neglect (rate, %)<br>Physical Neglect (rate, %)	
Conclusion/Comments	The abuse reported were more occasional experiences rather than frequent reporting nonetheless as 89.9% of any abuse in the past one year and 91% in the lifetime. First study in Indian diaspora to use the ICAST-CH tool to estimate household abuse. Did not include external environments such as schools.
Conflicts of Interest<br><br>Ethical Approval	None declared<br><br>Ethical approval sought from local authority and educational ministry to conduct the research.
Funders	Municipal Corporation of Thrissur city

Table 2. Example of a data extraction form [26]

Quality Assessment

The final selected studies will be appraised for quality by the first author and, to ensure robustness, they will also be quality appraised by a second reviewer. A third reviewer will resolve any conflicts, with support from the expert advisory panel if required.
Study quality (largely cross-sectional and case-control) will be assessed using the Newcastle-Ottawa Quality Assessment Scale (NOS) adapted for use in cross-sectional study designs as it considers key areas: selection (representativeness of the sample, sample size, non-responders and exposure details); comparability (is confounding considered) and, outcome (blinding, recording and statistical test used) [78,79]. Whilst there are a myriad range of assessment tools available, the NOS has been endorsed by the Cochrane Collaboration to assess quality of research studies [76]. An example of a NOS assessment of a sample study is presented in Appendix 3.

Data synthesis

The study will include a narrative synthesis of the findings and attempt a meta-analysis dependant on heterogeneity. A meta-analytic approach will be used including any

For peer review only - http://bmjopen.bmj.com/site/about/guidelines.xhtml

random-effect pooled prevalence of child maltreatment stratified by gender, urbanisation and pre-and post-POCSO Act. Heterogeneity will be evaluated using forest plots and measuring the impact of the I^2 statistic (I^2 >50% indicates significant heterogeneity) and tested with the χ^2 statistic ($p<0.05$) [62]. We will also report tau^2 values to indicate any between-study variance. We will use a fixed effects model for meta-analysis, except where we identify statistical heterogeneity in which case, we would use a random effects Poisson model [80] to determine an 'average prevalence' across the population of all included studies. We will use the 'metaprop' command to perform meta-analyses of proportions of child maltreatment in STATA, which builds on the 'metan' command [81] but is typically used to pool effects such as proportions from cross-sectional studies instead of odds ratios from case-control studies.

In the case of significant heterogeneity derived from our analysis, we will attempt to create subgroups of the paediatric population to understand any extracted data. This could include different age-bands of participants (e,g, child (0-12 years), adolescent (13-18 years) using WHO cut-offs for age bands in paediatric populations. We may also wish to compare prevalence rates of child maltreatment based on self-report measures versus objectively assessed measures as well as compare rates by time (one year compared with lifetime occurrence or frequency data).

Meta-bia(s)

Research papers written originally in Hindi or any other Indian dialect, that would otherwise be included in the data capture, will not be used for the purpose of this study.

For peer review only

Discussion

The findings of this study will aim to add to the limited knowledge of child maltreatment in India and specifically, the impact of gender, urbanisation and the POCSO Act on the prevalence and determinants of child maltreatment. There are known gaps in the evidence base with regards to the overlap of different forms of child maltreatment and specifically, the gendered effects of child maltreatment. These include the identification and motivation of perpetrators and what happens after maltreatment has happened – a child's awareness of their rights and attitudes towards abuse, neglect and reporting of child maltreatment. The circumstances that encourage reporting, details on protective factors and risk factors of child maltreatment have received limited attention in the literature [22].

Furthermore, the impact of child maltreatment and where they occur (e.g. urban areas versus slum areas) is important as these may offer an insight into potential interventional pathways that differ by geography e.g. enforcing child welfare policies in rural areas [22]. The impacts of urban and rural poverty, socioeconomic status; family income; parental occupation; family structure (nuclear, joint, extended, etc) and other confounder data will be collated during this review to aid in the interpretation of results including subgroup analysis and meta-analysis. Furthermore, the impact of this study on policymaking will come from its inclusion of the only legislative act in India that focusses on child maltreatment. The Protection of Children from Sexual Offences Act (POCSO Act) was established in 2012 by the Indian government to protect children against offences like sexual abuse. The Act makes it mandatory to report acts of sexual offence and document reporting timelines, prosecution timelines and clearly record all evidence (within a 30-day period). Whilst POSCO has been a relatively recent Act, its effect on numbers of reported offences in research studies has yet to be determined.

Implications on Policy and Research

When the evidence on child maltreatment in India is collated and presented, any policy or practice impacts can be tailor-made based on the country-specific context and findings. In India, the challenge will come from addressing cultural norms and traditional values (e.g. gender inequity and reducing child marriages), creating safer environments for children within homes and schools (e.g. authorities penalising assault) and scaling up response and support services for children in different settings such as tribal regions, slum areas, rural communities (e.g. trauma informed health and social care). This approach has worked in higher-income countries but in LMIC's like India, there are challenges. These include low levels of trauma-informed care for injured children in hospital settings with 91% of healthcare workers not receiving any trauma-informed training [82]. Other challenges included overburdened judicial and criminal systems; poor child welfare services; gender inequality and illiteracy [22]. This study will capture data on health inequities and social determinants that predispose to maltreatment in different geographical areas of India including confounders such as poverty and neglect.

Strengths and Limitations

There are some strengths to our approaches. This is the first review undertaken focussing on all aspects of child maltreatment in India where previous review articles

For peer review only

and studies have focussed on a specific facet of maltreatment, e.g. child sexual abuse. Secondly, the inclusion of the POCSO Act makes this review nuanced to comment on any effect of legislation where it has never been formally evaluated [83]. This study will include an analysis of child maltreatment studies and corresponding prevalence rates, both pre-and post-2012. With the inclusion of the POCSO 2012 threshold, it is expected that there will be an increased number of cases reporting all forms of child maltreatment. Thirdly, the authors will attempt to meta-analyse the data depending on heterogeneity scores generated. Choudhry's work [22] highlighted the significant heterogeneity of studies conducted to date and this might limit the quality of meta-analytics possible given the discrepancies in samples size. Possible reasons for heterogeneity include varying age ranges of participants, selection bias with sample provenance (e.g recruitment from observation homes vs. school children), varying urbanisation metrics (e.g. samples including children from all types of socioeconomic backgrounds) and variation in outcome assessment definition (e.g. self-report vs. validated questionnaire).

There are limitations to this review. Firstly, only papers in English were included due to limited access to funding and resources such as a professional translator. We acknowledge the importance of Anglophone bias in the context of this Indian based study therefore endeavour to ensure future extensions of this research does include non-English papers Secondly, excluding grey literature from OPEN Grey, narrative reports from non-governmental organisations or World Bank reports would limit some of the 'unmeasured' exposures and confounders that may increase child maltreatment [25, 49]. However, the authors will make attempts to collate this additional information so that they may be presented as part of a narrative synthesis on the topic of child maltreatment in India, thus improving the quality, generalisability and relevance of our work [84]. The exclusion of national statistics (e.g. NCRB records) might limit interpretation of any effect of the POCSO Act on conviction rates for child maltreatment. Qualitative studies were also excluded from this review which may not effect prevalence rates of child maltreatment but would allow exploration of non-responses from children (interpretative) or indeed, highlight determinants that have not been identified in quantitative or aggregated reviews [85-87].

Conclusion

This review will build on these thematic and methodological gaps in the literature, synthesise the evidence by gender, urbanisations and POCSO effects and provide practice, policy and clinical solutions that may help reduce the occurrence and impact of child maltreatment in India.

For peer review only - http://bmjopen.bmj.com/site/about/guidelines.xhtml

Acknowledgements: The authors would like to extend their sincere gratitude to Mrs Sarah Herring, Subject Librarian for Population Health Sciences, University of Bristol for her advice and guidance during the protocol development stage.

Contributorship statement: GF, RG and EP were responsible for the initial protocol drafting and design. GF, MF, NV, PD then contributed to preliminary searches used to develop the rationale and background of the study. ES, BH, VC, VB, PD & MF were on the expert advisory panel for the study to advise on methods design and statistical analysis techniques that could be used. All authors contributed and approved this final manuscript.

Competing Interests: None to declare.

Funding: Dr Gwen Fernandes is funded by the UK Medical Research Council and Alcohol Research UK Grant (MR/L022206/1).

Ethics Statements: The protocol and Level 1 self-audit checklist was submitted and approved by the Usher Research Ethics Group (UREG) panel in the Usher Institute (School of Medicine and Veterinary Sciences) at the University of Edinburgh [Reference B126255]

Data Sharing Statement: Data sharing is not applicable to this article as no new data were created or analysed in this study.

For peer review only

References

1. WORLD HEALTH ORGANISATION, 2019. Children: reducing mortality. Available from: https://www.who.int/news-room/fact-sheets/detail/children-reducing-mortality [Accessed 2nd February 2020]

2. GRIFFITHS, S., HUNTER, DJ, 2007. New Perspective in Public Health. London: Radcliffe Publishing.

3. REYNOLDS, A.J., ROLNICK, A.J., TEMPLE, J.A., 2015. Health and Education in Early Childhood: Predictors, Interventions and Policies. Cambridge: Cambridge University Press.

4. WORLD HEALTH ORGANISATION 2017. Country Cooperation Strategy at a glance: India. Available from: https://apps.who.int/iris/bitstream/handle/10665/136895/ccsbrief_ind_en.pdf;jsessionid=90BA4E2E08E57D23C4363B8FBDFC85AC?sequence=1 [Accessed 5th March 2020]

5. WORLD HEALTH ORGANISATION, 2006. Preventing Child Maltreatment: a guide to taking action and generating evidence. Available from: https://apps.who.int/iris/bitstream/handle/10665/43499/9241594365_eng.pdf;jsessionid=555709D037DBAD5DE822D439283A5F1B?sequence=1 [Accessed 1st of February 2020]

6. BLUM, R. W., LI, M. & NARANJO-RIVERA, G. 2019. Measuring Adverse Child Experiences Among Young Adolescents Globally: Relationships With Depressive Symptoms and Violence Perpetration. *Journal of Adolescent Health,* 65**,** 86-93.

7. BELLIS, M. A., HUGHES, K., FORD, K., RAMOS RODRIGUEZ, G., SETHI, D. & PASSMORE, J. 2019. Life course health consequences and associated annual costs of adverse childhood experiences across Europe and North America: a systematic review and meta-analysis. *The Lancet Public Health,* 4**,** e517-e528.

8. SHARMA, E., VAIDYA, N., IYENGAR, U., ZHANG, Y., HOLLA, B., PURUSHOTTAM, M., CHAKRABARTI, A., FERNANDES, G. S., HERON, J., HICKMAN, M., DESRIVIERES, S., KARTIK, K., JACOB, P., RANGASWAMY, M., BHARATH, R. D., BARKER, G., ORFANOS, D. P., AHUJA, C., MURTHY, P., JAIN, S., VARGHESE, M., JAYARAJAN, D., KUMAR, K., THENNARASU, K., BASU, D., SUBODH, B. N., KURIYAN, R., KURPAD, S. S., KALYANRAM, K., KRISHNAVENI, G., KRISHNA, M., SINGH, R. L., SINGH, L. R., KALYANRAM, K., TOLEDANO, M., SCHUMANN, G., BENEGAL, V. & THE C, V. C. 2020. Consortium on Vulnerability to Externalizing Disorders and Addictions (cVEDA): A developmental cohort study protocol. *BMC Psychiatry,* 20**,** 2.

9. DAMODARAN, D. K. & PAUL, V. K. 2017. Patterning/Clustering of Adverse Childhood Experiences (ACEs): The Indian Scenario. *Psychological Studies,* 62**,** 75-84.

10. HUGHES, K., BELLIS, M. A., HARDCASTLE, K. A., SETHI, D., BUTCHART, A., MIKTON, C., JONES, L. & DUNNE, M. P. 2017. The effect of multiple adverse childhood experiences on health: a systematic review and meta-analysis. *The Lancet Public Health,* 2**,** e356-e366.

For peer review only - http://bmjopen.bmj.com/site/about/guidelines.xhtml

11. KOWAL, P. & AFSHAR, S. 2015. Health and the Indian caste system. *The Lancet,* 385**,** 415-416.

12. VIRANI, P., 2000. Bitter Chocolate. New Delhi: Penguin Books.

13. NIJHARA, K., BHATIA, S. & UNNIKRISHNAN, B. 2018. Corporal Punishment in Children and its Implications on Mental Health. *The Indian Journal of Pediatrics,* 85**,** 405-405.

14. KOCHANEK, S.A., and HARDGRAVE, R.L., 2008. India: Government and Politics in a Developing Nation. Boston: Thomson Higher Education.

15. UNICEF 2014. Ending violence against children in Asia. Available from: https://www.unicef.org/publications/files/Ending_Violence_Against_Children_Six_strategies_for_action_EN_9_Oct_2014.pdf [Accessed 24th Jan 2019]

16. ANDA, R. F., FELITTI, V. J., BREMNER, J. D., WALKER, J. D., WHITFIELD, C., PERRY, B. D., DUBE, S. R. & GILES, W. H. 2006. The enduring effects of abuse and related adverse experiences in childhood. *European Archives of Psychiatry and Clinical Neuroscience,* 256**,** 174-186.

17. PECHTEL, P. & PIZZAGALLI, D. A. 2011. Effects of early life stress on cognitive and affective function: an integrated review of human literature. *Psychopharmacology (Berl),* 214**,** 55-70.

18. DANESE, A. & MCEWEN, B. S. 2012. Adverse childhood experiences, allostasis, allostatic load, and age-related disease. *Physiol Behav,* 106**,** 29-39.

19. BEHERE, P. B., SATHYANARAYANA RAO, T. S. & MULMULE, A. N. 2013. Sexual abuse in women with special reference to children: Barriers, boundaries and beyond. *Indian journal of psychiatry,* 55**,** 316-319.

20. NATIONAL CRIME RECORDS BUREAU. 2016. Crime in India – Statistics. Available from: http://ncrb.gov.in/StatPublications/CII/CII2016/Press%20release-Crime%20in%20India%202016.pdf [Accessed 14th Feb 2019]

21. STOLTENBORGH, M., VAN IJZENDOORN, M. H., EUSER, E. M. & BAKERMANS-KRANENBURG, M. J. 2011. A global perspective on child sexual abuse: meta-analysis of prevalence around the world. *Child Maltreat,* 16**,** 79-101.

22. CHOUDHRY, V., DAYAL, R., PILLAI, D., KALOKHE, A. S., BEIER, K. & PATEL, V. 2018. Child sexual abuse in India: A systematic review. *PLoS One,* 13**,** e0205086.

23. WORLD HEALTH ORGANISATION, 2016. CHILD MALTREATMENT. Available from: https://www.who.int/news-room/fact-sheets/detail/child-maltreatment [Accessed 16th October 2019]

24. RADHIKA, K., MANJULA, M. & JAISOORYA, T. S. 2018. Ethical gaps in conducting research among adult survivors of child sexual abuse: a review. *Indian J Med Ethics,* 3**,** 186-192.

25. SETH, R., BOSE, V., QAIYUM, Y., CHANDRASHEKHAR, R., KANSAL, S., TANEJA, I. & SETH, T. 2018. Social Determinants of Child Marriage in Rural India. *Ochsner J,* 18**,** 390-394.

For peer review only - http://bmjopen.bmj.com/site/about/guidelines.xhtml

26. KUMAR, M. T., KAR, N. & KUMAR, S. 2019. Prevalence of child abuse in Kerala, India: An ICAST-CH based survey. *Child Abuse Negl,* 89**,** 87-98.

27. HAILES, H. P., YU, R., DANESE, A. & FAZEL, S. 2019. Long-term outcomes of childhood sexual abuse: an umbrella review. *The Lancet Psychiatry,* 6**,** 830-839.

28. DATT, G., RAVALLION, M. & MURGAI, R. 2020. Poverty and Growth in India over Six Decades. *American Journal of Agricultural Economics,* 102**,** 4-27.

29. World Bank Data. Population Living in Slums. Available from: https://data.worldbank.org/indicator/EN.POP.SLUM.UR.ZS?locations=IN [Accessed 5th February 2021]

30. BYWATERS, P, BUNTING, L, DAVIDSON G, HANRATTY J, MASON W, MCCARTAN C, AND STEILS, N, 2016. The relationship between poverty, child abuse and neglect: an evidence review. Available from: file:///C:/Users/gf18522/Downloads/bywaters_can_final_report.pdf [Accessed 6th June 2019]

31. DEB, S. & MODAK, S. 2010. Prevalence of violence against children in families in Tripura and its relationship with socio-economic factors. *J Inj Violence Res,* 2**,** 5-18.

32. BANDEWAR, S. V., PITRE, A. & LINGAM, L. 2018. Five years post Nirbhaya: Critical insights into the status of response to sexual assault. *Indian J Med Ethics,* 3**,** 215-221.

33. NATIONAL FAMILY HEALTH SURVEY. 2016. Available from: http://rchiips.org/nfhs/factsheet_NFHS-4.shtml [Accessed 16th Jan 2019]

34. CARSON, D.K., FOSTER, J.M., CHOWDHURY, A., 2014. A. Sexual Abuse of Children and Youth in India: An Anthropological Perspective. The Oriental Anthropologist, 14(2):343.

35. FELITTI, V. J., ANDA, R. F., NORDENBERG, D., WILLIAMSON, D. F., SPITZ, A. M., EDWARDS, V., KOSS, M. P. & MARKS, J. S. 1998. Relationship of childhood abuse and household dysfunction to many of the leading causes of death in adults. The Adverse Childhood Experiences (ACE) Study. *Am J Prev Med,* 14**,** 245-58.

36. SPRINGER, K. W., SHERIDAN, J., KUO, D. & CARNES, M. 2007. Long-term physical and mental health consequences of childhood physical abuse: results from a large population-based sample of men and women. *Child Abuse Negl,* 31**,** 517-30.

37. KESSLER, R. C., MCLAUGHLIN, K. A., GREEN, J. G., GRUBER, M. J., SAMPSON, N. A., ZASLAVSKY, A. M., AGUILAR-GAXIOLA, S., ALHAMZAWI, A. O., ALONSO, J., ANGERMEYER, M., BENJET, C., BROMET, E., CHATTERJI, S., DE GIROLAMO, G., DEMYTTENAERE, K., FAYYAD, J., FLORESCU, S., GAL, G., GUREJE, O., HARO, J. M., HU, C. Y., KARAM, E. G., KAWAKAMI, N., LEE, S., LEPINE, J. P., ORMEL, J., POSADA-VILLA, J., SAGAR, R., TSANG, A., USTUN, T. B., VASSILEV, S., VIANA, M. C. & WILLIAMS, D. R. 2010. Childhood adversities and adult psychopathology in the WHO World Mental Health Surveys. *Br J Psychiatry,* 197**,** 378-85.

For peer review only

38. IP, P., WONG, R. S., LI, S. L., CHAN, K. L., HO, F. K. & CHOW, C. B. 2016. Mental Health Consequences of Childhood Physical Abuse in Chinese Populations: A Meta-Analysis. *Trauma Violence Abuse,* 17**,** 571-584.

39. KELLY-IRVING, M. & DELPIERRE, C. 2019. A Critique of the Adverse Childhood Experiences Framework in Epidemiology and Public Health: Uses and Misuses. *Social Policy and Society,* 18**,** 445-456.

40. HOUTEPEN, L.C., HERON, J., SUDERMAN, M.J, TILLING, K., HOWE, L.D., 2018. Adverse childhood experiences in the children of the Avon Longitudinal Study of Parents and Children (ALSPAC). *Wellcome Open Res.*;3:106.

41. GILBERT, R., WIDOM, C. S., BROWNE, K., FERGUSSON, D., WEBB, E. & JANSON, S. 2009. Burden and consequences of child maltreatment in high-income countries. *Lancet,* 373**,** 68-81.

42. KIBURI, S. K., MOLEBATSI, K., OBONDO, A. & KURIA, M. W. 2018. Adverse childhood experiences among patients with substance use disorders at a referral psychiatric hospital in Kenya. *BMC Psychiatry,* 18**,** 197.

43. SIDEBOTHAM, P.D., 2001. An ecological approach to child abuse: a creative use of scientific models in research and practice. *Child Abuse Review*, 10: 97-112.

44. DAHLBERG, L.L., KRUG, E.G., Violence-a global public health problem. In: Krug E, Dahlberg LL, Mercy JA, Zwi AB, Lozano R, eds. World Report on Violence and Health. Geneva, Switzerland: World Health Organization; 2002:1–56.

45. SIDEBOTHAM P, HERON, J and ALSPAC Study Team., 2006. Child maltreatment in the "children of the nineties": a cohort study of risk factors. Child Abuse and Neglect; 30 (5): 497-522.

46. THOMAS REUTERS, 2018. India: the worlds most dangerous countries for women in 2018. Available from: http://poll2018.trust.org/country/?id=india [Accessed 2nd December 2019]

47. KACKER L, VARDAN S, KUMAR P, 2007. Study on Child Abuse: Ministry of Women and Child Development, Government of India.

48. MACIONNIS, J, PLUMMER, K, 2012. Sociology: a global introduction. United Kingdom: Harlow.

49. SUBRAMANIYAN, V. K. S., REDDY, P., CHANDRA, G., RAO, C. & RAO, T. S. S. 2017. Silence of male child sexual abuse in India: Qualitative analysis of barriers for seeking psychiatric help in a multidisciplinary unit in a general hospital. *Indian journal of psychiatry,* 59**,** 202-207.

50. DEB, S, 2018. An empirical investigation into child abuse and neglect in India: burden, impact and protective measures. Pondicherry: Springer.

51. FLEDDERJOHANN, J., AGRAWAL, S., VELLAKKAL, S., BASU, S., CAMPBELL, O., DOYLE, P., EBRAHIM, S. & STUCKLER, D, 2014. Do girls have a nutritional disadvantage compared with boys? Statistical models of breastfeeding and food consumption inequalities among Indian siblings. *PLoS One,* 9**,** e107172.

For peer review only - http://bmjopen.bmj.com/site/about/guidelines.xhtml

52. AURINO, E. 2017. Do boys eat better than girls in India? Longitudinal evidence on dietary diversity and food consumption disparities among children and adolescents. *Economics & Human Biology,* 25**,** 99-111.

53. KSHATRIYA, G. K. & ACHARYA, S. K. 2016. Gender Disparities in the Prevalence of Undernutrition and the Higher Risk among the Young Women of Indian Tribes. *PloS one,* 11**,** e0158308-e0158308.

54. PILLAI, A., ANDREWS, T. & PATEL, V. 2008. Violence, psychological distress and the risk of suicidal behaviour in young people in India. *International Journal of Epidemiology,* 38**,** 459-469.

55. BALAJI, M., ANDREWS, T., ANDREW, G. & PATEL, V. 2011. The acceptability, feasibility, and effectiveness of a population-based intervention to promote youth health: an exploratory study in Goa, India. *J Adolesc Health,* 48**,** 453-60.

56. JAYA, J. & HINDIN, M. J. 2007. Nonconsensual sexual experiences of adolescents in urban India. *J Adolesc Health,* 40**,** 573.e7-14.

57. BANERJEE, S.R., 2001. Physical abuse of street and slum children of Kolkata. Indian Paediatrics, 38, 1163-1170.

58. SAVE THE CHILDREN, 2015. Forgotten voices; The world of urban children in India. Available from: https://resourcecentre.savethechildren.net/library/forgotten-voices-world-urban-children-india [Accessed 1st March 2020]

59. STROCHLIC, N. 2018. India's forgotten child brides. Available from: https://www.nationalgeographic.com/photography/proof/2018/04/child-brides-marriage-shravasti-india-culture/ [Accessed 5th March 2020]

60. SATHYANARAYANA RAO, T. S., NAGPAL, M. & ANDRADE, C. 2013. Sexual coercion: Time to rise to the challenge. *Indian journal of psychiatry,* 55**,** 211-213.

61. HARBISHETTAR, V. & MATH, S. B. 2014. Violence against women in India: comprehensive care for survivors. *Indian J Med Res,* 140**,** 157-9.

62. HIGGINS, J.P., THOMPSON, S.G., 2002. Quantifying heterogeneity in a meta-analysis. Stats Med; 21; 1539-1558.

63. MOHER, D., LIBERATI, A., TETZLAFF, J. & ALTMAN, D. G. 2009. Preferred reporting items for systematic reviews and meta-analyses: the PRISMA statement. *PLoS Med,* 6**,** e1000097.

64. VERGNES, J. N., MARCHAL-SIXOU, C., NABET, C., MARET, D. & HAMEL, O. 2010.Ethics in systematic reviews. *J Med Ethics,* 36**,** 771-4.

65. WORLD HEALTH ORGANISATION 2018. Adverse Childhood Experiences International Questionnaire. In Adverse Childhood Experiences International Questionnaire (ACE-IQ). Available from: http://www.who.int/violence_injury_prevention/violence/activities/adverse_childhood_experiences [Accessed 1st of February 2019]

66. DAY, C, HIBBERT, P, CADMAN, S, 2008. A literature review into children abused and/or neglected prior to custody. Available from:

For peer review only - http://bmjopen.bmj.com/site/about/guidelines.xhtml

https://dera.ioe.ac.uk/7997/1/Abused%20prior%20to%20custody.pdf [Accessed 5th March 2020]

67. BALDWIN, J. R., REUBEN, A., NEWBURY, J. B. & DANESE, A. 2019. Agreement Between Prospective and Retrospective Measures of Childhood Maltreatment: A Systematic Review and Meta-analysis. *JAMA Psychiatry,* 76**,** 584-593.

68. TEICHER, M. H., SAMSON, J. A., ANDERSON, C. M. & OHASHI, K. 2016. The effects of childhood maltreatment on brain structure, function and connectivity. *Nat Rev Neurosci,* 17**,** 652-66.

69. COLMAN I, KINGSBURY M, GARAD Y, et al. 2016 Consistency in adult reporting of adverse childhood experiences. *Psychol Med*. ;46(3):543-549.

70. EICHENBAUM, H. 2017. Memory: Organization and Control. *Annu Rev Psychol,* 68**,** 19-45.

71. RASCH, B. & BORN, J. 2013. About sleep's role in memory. *Physiol Rev,* 93**,** 681-766.

72. NSPCC 2017. Definitions and signs of child abuse. Available from: - https://learning.nspcc.org.uk/media/1188/definitions-signs-child-abuse.pdf [Accessed 20th December]

73. SINGHI, P., SAINI, A. G. & MALHI, P. 2013. Child maltreatment in India. *Paediatrics and International Child Health,* 33**,** 292-300.

74. BEATTIE, T. S., PRAKASH, R., MAZZUCA, A., KELLY, L., JAVALKAR, P., RAGHAVENDRA, T., RAMANAIK, S., COLLUMBIEN, M., MOSES, S., HEISE, L., ISAC, S. & WATTS, C. 2019. Prevalence and correlates of psychological distress among 13-14 year old adolescent girls in North Karnataka, South India: a cross-sectional study. *BMC Public Health,* 19**,** 48.

75. SALVADOR-OLIVÁN, J. A., MARCO-CUENCA, G. & ARQUERO-AVILÉS, R. 2019. Errors in search strategies used in systematic reviews and their effects on information retrieval. *Journal of the Medical Library Association : JMLA,* 107**,** 210-221.

76. HIGGINS JPT, GREEN S, editors. Cochrane Handbook for Systematic Reviews of Interventions, Version 5.1.0. London: The Cochrane Collaboration; 2011. Section 13.5.2.3. Tools for assessing methodological quality or risk of bias in non-randomized studies.

77. OUZZANI, M., HAMMADY, H., FEDOROWICZ, Z, ELMAGARMID, A., 2016. Rayyan-a web and mobile app for systematic reviews. Systematic Reviews: 5:210.

78. WELLS, G.A., SHEA, B., O'CONNELL. D. PETERSON, J., WELCH, V., LOSOS, M., P.T. 2014. The Newcastle-Ottawa Scale (NOS) for assessing the quality of nonrandomised studies in meta-analyses. Available from: http://www.ohri.ca/programs/clinical_epidemiology/oxford.asp [Accessed 1st of Feb 2019]

79. HERZOG, R., ALVAREZ-PASQUIN, M. J., DIAZ, C., DEL BARRIO, J. L., ESTRADA, J. M. & GIL, A. 2013. Are healthcare workers' intentions to vaccinate related to their knowledge, beliefs and attitudes? A systematic review. *BMC Public Health,* 13**,** 154

For peer review only - http://bmjopen.bmj.com/site/about/guidelines.xhtml

80. DEEKS JJ, HIGGINS JPT, ALTMAN DG (editors). Chapter 10: Analysing data and undertaking meta-analyses. In: Higgins JPT, Thomas J, Chandler J, Cumpston M, Li T, Page MJ, Welch VA (editors). *Cochrane Handbook for Systematic Reviews of Interventions* version 6.0 (updated July 2019). Cochrane, 2019. Available from www.training.cochrane.org/handbook [Accessed 1st of January 2020]

81. NYAGA, V. N., ARBYN, M. & AERTS, M. 2014. Metaprop: a Stata command to perform meta-analysis of binomial data. *Archives of Public Health,* 72**,** 39.

82. HOYSTED, C., BABL, F. E., KASSAM-ADAMS, N., LANDOLT, M. A., JOBSON, L., VAN DER WESTHUIZEN, C., CURTIS, S., KHARBANDA, A. B., LYTTLE, M. D., PARRI, N., STANLEY, R. & ALISIC, E. 2018. Knowledge and training in paediatric medical traumatic stress and trauma-informed care among emergency medical professionals in low- and middle-income countries. *European journal of psychotraumatology,* 9**,** 1468703-1468703.

83. SETH, R & SRIVASTAVA, RN. 2017. Child Sexual Abuse: Management and Prevention, and protection of children from sexual offences (POCSO) Act. Indian paediatrics. 54.

84. PAEZ, A. 2017. Gray literature: An important resource in systematic reviews. *J Evid Based Med*. 10: 233– 240.

85. NOBLIT, G.W, HARE RD, 1988. Meta-ethnography: synthesizing qualitative studies, vol. 11. California: Sage Publications.

86. HANNES, K, AND LOCKWOOD, C., 2012. Synthesising qualitative research: choosing the right approach. John Wiley and Sons.

87. SEERS, K. 2015. Qualitative systematic reviews: their importance for our understanding of research relevant to pain. *Br J Pain,* 9**,** 36-40.

For peer review only

Figure 1. World map showing the rights of a child as measured by UNICEF and World Bank data (2015).

Figure 2. Adverse Childhood Experiences (ACEs) including traditional direct ACEs (child maltreatment: physical, sexual and emotional abuse and neglect) and indirect ACEs (Felitti et al., 1998) and further extended by researchers since the original study (Houtepen et al., 2018)

Figure 3. Potential pathways explaining child maltreatment using the socioecological model proposed by Sidebotham and Heron (2006)

For peer review only

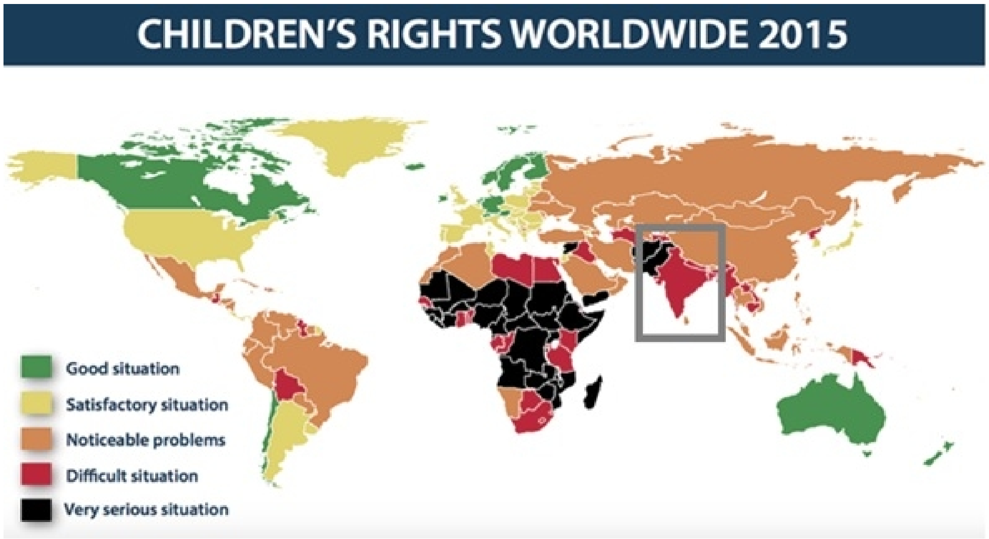

Figure 1. World map showing the rights of a child as measured by UNICEF and World Bank data (2015).

25x13mm (600 x 600 DPI)

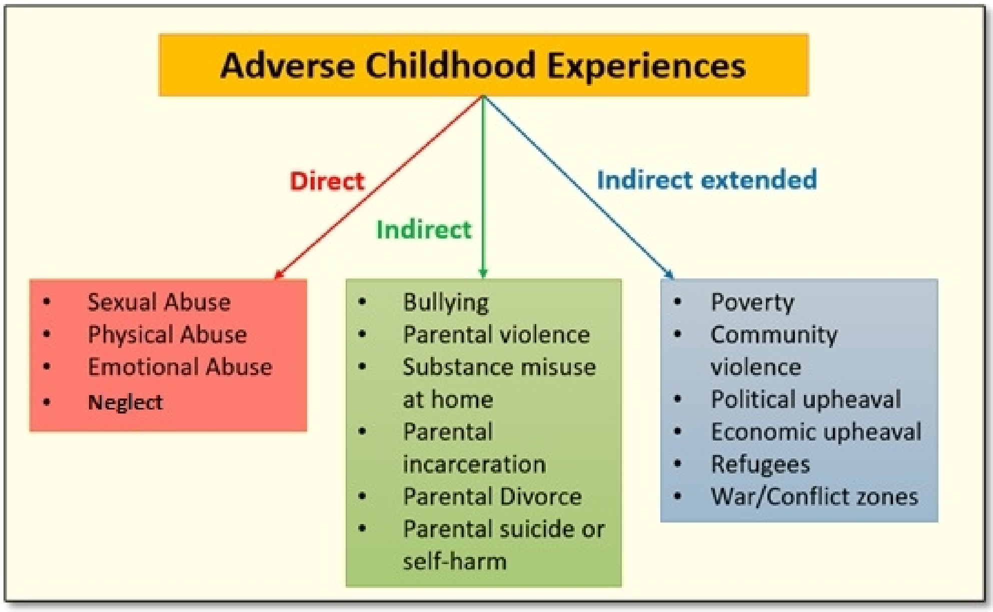

Figure 2. Adverse Childhood Experiences (ACEs) including traditional direct ACEs (child maltreatment: physical, sexual and emotional abuse and neglect) and indirect ACEs (Felitti et al., 1998) and further extended by researchers since the original study (Houtepen et al., 2018)

23x14mm (600 x 600 DPI)

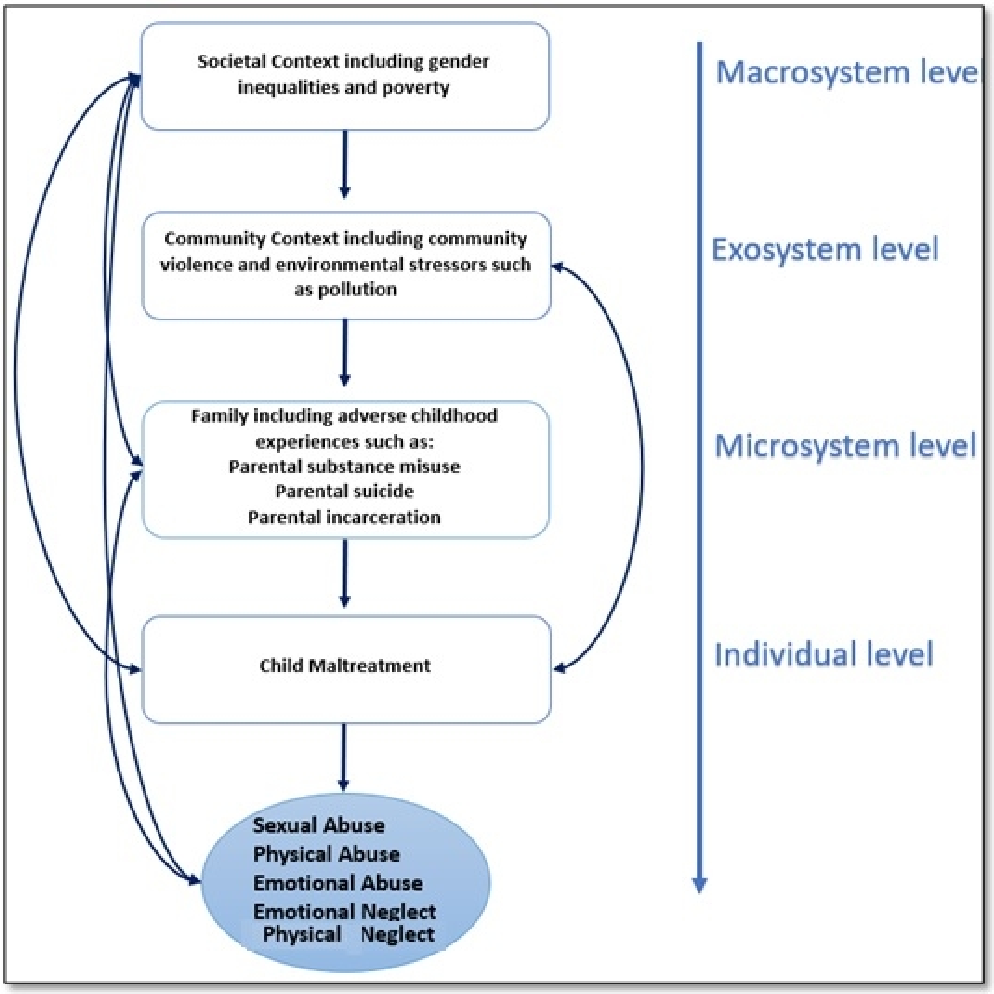

Figure 3. Potential pathways explaining child maltreatment using the socioecological model proposed by Sidebotham and Heron (2006)

22x22mm (600 x 600 DPI)

Appendix 1 - Experts in the field who contributed to the research study design

**Member, Designation & Location**	**Responsibilities**
**Nilakshi Vaidya, Psychology Counsellor, National Institute of Mental Health and Neurosciences (NIMHANS) (India) –** 2nd reviewer	Will be the second reviewer for the systematic review and will review 20% of the article titles, abstract and full texts for inclusion criteria. Will be the second author on any journal paper published.
**Megan Fernandes, Core Surgical Trainee with experience working in pediatric medicine and surgery (UK)-**3rd reviewer	Will be the third reviewer for the systematic review and will review 20% of the article titles, abstract and full texts for inclusion criteria (published pre-2012). Will be the third author on any journal paper published.
**Phillip De Souza, Core Internal Medicine Trainee, experience working in internal medicine (UK) -**4th reviewer	Will be the fourth reviewer for the systematic review and will review 20% of the article titles, abstract and full texts for inclusion criteria (published post-2012). Will be the fourth author on any journal paper published.
**Vikas Choudhry, Director, Vice President of Public Health, SRC (Uttar Pradesh)**	Regular contact through the systematic review process. Will input on search strategies and will specifically resolve any conflicts between myself and the second reviewer.<br>Will be the final author (senior author) on any journal publication.
**Bharath Holla, Clinical Psychiatrist and Associate Professor, NIMHANS (India)**	PPI group for input on search words and search strategies
**Vivek Benegal, Clinical Psychiatrist and cVEDA Principal Investigator, NIMHANS (India)**	Clinical psychiatrist and expert in child trauma and informing trauma-based policies in India. Regular contact through the review process and specific input on the search strategies and key words and MESH terms used. Penultimate senior author on any publication.

For peer review only - http://bmjopen.bmj.com/site/about/guidelines.xhtml

Appendix 2a. PubMed search strategy

a) Child sexual abuse:

(((((((child sexual abuse) OR child sexual coercion) OR child sexual offender)) OR (((child sexual assault) OR child sexual porn*) OR child sexual molest*)) OR (((((child sexual crime) OR child sexual aggression) OR child sexual offence) OR child sexual violence) OR child rape)) OR ((child rape) OR child incest)) AND India

b) Child emotional abuse:

((((((((((child worthless) OR child unloved) OR child inadequate) OR child failure) OR child corrupt*) OR child unheard) OR child cruelty) OR child emo* AND abuse) OR child maltreat*) AND India)) AND (((((((((((child denial*) OR child denial*) OR child denial shelter*) OR child carer failure) OR child parental failure) OR child emo*) OR child emo* AND danger) OR child neglect) OR child abandonment*) OR child bereavment*) OR child grief) AND India[all])

c) Child physical abuse:

(((((((((((child hitting) OR child shaking) OR child throwing) OR child poisoning) OR child burning) OR child scalding) OR child drowning) OR child suffoc*) OR child beating) OR child physical violence) OR child past*) OR child rap) AND India

Child neglect:

d) (((((child neglect) OR child psycho* AND assault) OR child denial) OR child emo* AND assualt) OR child malnu*) AND India

For peer review only

Appendix 2b EMBASE search strategy

a) Child sexual abuse
1. Prevalence/
2. Limit 1 to human
3. ("India" or "india").mp.[mp=title, abstract, heading word, drug trade name, original title, device manufacturer, drug manufacturer, device trade name, keyword, floating subheading word]
4. (child sexual abuse or "child sexual coercion" or "child sexual offender" or "child sexual assault" or "child sexual porn*" or "child sexual molest*" or "child sexual crime" or "child sexual aggression" or "child sexual offence") or "child sexual violence" or "child rape" or "child rape" or "child incest").mp. [mp=title, abstract, heading word, drug trade name, original title, device manufacturer, drug manufacturer, device trade name, keyword, floating subheading word]
5. 1 and 2 and 3 and 4
6. Limit 5 to yr= "2005 to 2020"

b) child emotional abuse
1. Prevalence/
2. Limit 1 to human
3. ("India" or "india").mp.[mp=title, abstract, heading word, drug trade name, original title, device manufacturer, drug manufacturer, device trade name, keyword, floating subheading word]
4. (child worthless or "child unloved" or "child inadequate" or "child failure" or "child corrupt" or "child unheard" or "child cruelty" or "child emo*" or "child emo abuse" or "child maltreat" or "child denial" or "child denial shelter" or "child carer failure" or "child parental failure" or "child emo*" or "child emo* and danger" or "child neglect" or "child abandonment*" or "child bereavment*" or "child grief")mp.[mp=title, abstract, heading word, drug trade name, original title, device manufacturer, drug manufacturer, device trade name, keyword, floating subheading word]
5. 1 and 2 and 3 and 4
6. Limit 5 to yr= "2005 to 2020"

c) Child physical abuse
1. Prevalence/
2. Limit 1 to human
3. ("India" or "india").mp.[mp=title, abstract, heading word, drug trade name, original title, device manufacturer, drug manufacturer, device trade name, keyword, floating subheading word]
4. (child hitting or "child shaking" or "child throwing" or "child poisoning" or "child burning" or "child scalding" or "child drowning" or "child suffoc*" or "child beating" or "child physical violence" or "child past*" or "child rap")mp.[mp=title, abstract, heading word, drug trade name, original title, device manufacturer, drug manufacturer, device trade name, keyword, floating subheading word]
5. 1 and 2 and 3 and 4
6. Limit 5 to yr= "2005 to 2020"

For peer review only

d) Child neglect

1. Prevalence/
2. Limit 1 to human
3. (“India” or “india”).mp.[mp=title, abstract, heading word, drug trade name, original title, device manufacturer, drug manufacturer, device trade name, keyword, floating subheading word]
4. (child neglect or ”child psycho*” or “assault” or “child denial” or “child emo*” or “assault*” or “child malnu”)mp.[mp=title, abstract, heading word, drug trade name, original title, device manufacturer, drug manufacturer, device trade name, keyword, floating subheading word]
5. 1 and 2 and 3 and 4
6. Limit 5 to yr= “2005 to 2020”

For peer review only

Appendix 2c COCHRANE search strategy

1. India or india
2. (child sexual abuse or child sexual coercion or child sexual offender or child sexual assault or child sexual porn or child sexual molest* or child sexual crime or child sexual aggression or child sexual offence or child sexual violence or child rape or child rape or child incest): ti,ab,kw
3. (child worthless or child unloved or child inadequate or child failure or child corrupt or child unheard or child cruelty or child emo* or child emo abuse or child maltreat or child denial or child denial shelter or child carer failure or child parental failure or child emo* or child emo* and danger or child neglect or child abandonment* or child bereavment* or child grief): ti,ab,kw
4. (child hitting or child shaking or child throwing or child poisoning or child burning or child scalding or child drowning or child suffoc* or child beating or child physical violence or child past* or child rap): ti,ab,kw
5. (child neglect or child psycho* or assault or child denial or child emo* or assault* or child malnu*): ti,ab,kw
6. {#2 or #3 or #4 or #5}
7. #1 and #6

For peer review only

Appendix 2d PsychInfo search strategy

1. Prevalence or epidemiology or frequency.tw.
2. child sexual abuse or child sexual coercion or child sexual offender or child sexual assault or child sexual porn or child sexual molest* or child sexual crime or child sexual aggression or child sexual offence or child sexual violence or child rape or child rape or child incest.tw,sh.
3. child worthless or child unloved or child inadequate or child failure or child corrupt or child unheard or child cruelty or child emo* or child emo abuse or child maltreat or child denial or child denial shelter or child carer failure or child parental failure or child emo* or child emo* and danger or child neglect or child abandonment* or child bereavment* or child grief.tw,sh.
4. child hitting or child shaking or child throwing or child poisoning or child burning or child scalding or child drowning or child suffoc* or child beating or child physical violence or child past* or child rap.tw,sh.
5. child neglect or child psycho* or assault or child denial or child emo* or assault* or child malnu*.tw,sh.
6. India
7. 1 or 2 or 3 or 4 or 5
8. 7 and 6
9. Limit 8 to (English language and yr=”2005-2020”)

For peer review only

Appendix 3 - **Newcastle-Ottawa Scale Quality Assessment**
Prevalence of child abuse in Kerala, India: An ICAST-CH based survey
Manoj Therayil Kumar, Nilamadhab Kar, Sebind Kumar

Selection:

1. Representativeness of the sample:
   a. Truly representative of the average in the target population. * (all subjects or random sampling)
   b. Somewhat representative of the average in the target group. * (non-random sampling)
   c. Selected group of users/convenience sample.
   d. No description of the derivation of the included subjects.

Points: *

2. Sample size:
   a. Justified and satisfactory (including sample size calculation). *
   b. Not justified.
   c. No information provided

Points: -

3. Non-respondents:
   a. Proportion of target sample recruited attains pre-specified target or basic summary of non-respondent characteristics in sampling frame recorded. *
   b. Unsatisfactory recruitment rate, no summary data on non-respondents.
   c. No information provided

Points: *

4. Ascertainment of the exposure (risk factor):
   a. Vaccine records/vaccine registry/clinic registers/hospital records only. **
   b. Parental or personal recall and vaccine/hospital records. *
   c. Parental/personal recall only.

Points: *

Comparability: (Maximum 2 stars)

1. Comparability of subjects in different outcome groups on the basis of design or analysis. Confounding factors controlled.
   a. Data/ results adjusted for relevant predictors/risk factors/confounders e.g. age, sex, time since vaccination, etc. **
   b. Data/results not adjusted for all relevant confounders/risk factors/information not provided.

Points: **

Outcome:

1. Assessment of outcome:
   a. Independent blind assessment using objective validated laboratory methods. **
   b. Unblinded assessment using objective validated laboratory methods. **
   c. Used non-standard or non-validated laboratory methods with gold standard. *
   d. No description/non-standard laboratory methods used.

Points: **

2. Statistical test:
   a. Statistical test used to analyse the data clearly described, appropriate and measures of association presented including confidence intervals and probability level (p value). *
   b. Statistical test not appropriate, not described or incomplete.

Points: *

Total Points scores: 8-star indicating a good study

Cross-sectional Studies:
Very Good Studies: 9-10 points
Good Studies: 7-8 points
Satisfactory Studies: 5-6 points
Unsatisfactory Studies: 0 to 4 points

For peer review only

Appendix 1 – PRISMA Checklist

Section/topic	#	Checklist item	Reported on page #
**TITLE**			
Title	1	Identify the report as a systematic review, meta-analysis, or both.	Page 1
**ABSTRACT**			
Structured summary	2	Provide a structured summary including, as applicable: background; objectives; data sources; study eligibility criteria, participants, and interventions; study appraisal and synthesis methods; results; limitations; conclusions and implications of key findings; systematic review registration number.	Page 2
**INTRODUCTION**			
Rationale	3	Describe the rationale for the review in the context of what is already known.	Pages 3-6
Objectives	4	Provide an explicit statement of questions being addressed with reference to participants, interventions, comparisons, outcomes, and study design (PICOS).	Pages 9
**METHODS**			
Protocol and registration	5	Indicate if a review protocol exists, if and where it can be accessed (e.g., Web address), and, if available, provide registration information including registration number.	Page 2
Eligibility criteria	6	Specify study characteristics (e.g., PICOS, length of follow-up) and report characteristics (e.g., years considered, language, publication status) used as criteria for eligibility, giving rationale.	Pages 10
Information sources	7	Describe all information sources (e.g., databases with dates of coverage, contact with study authors to identify additional studies) in the search and date last searched.	Page 10-12
Search	8	Present full electronic search strategy for at least one database, including any limits used, such that it could be repeated.	Page 12; Appendix 2
Study selection	9	State the process for selecting studies (i.e., screening, eligibility, included in systematic review, and, if applicable, included in the meta-analysis).	Page 13-16

For peer review only

Data collection process	10	Describe method of data extraction from reports (e.g., piloted forms, independently, in duplicate) and any processes for obtaining and confirming data from investigators.	Page 13
Data items	11	List and define all variables for which data were sought (e.g., PICOS, funding sources) and any assumptions and simplifications made.	Page 11
Risk of bias in individual studies	12	Describe methods used for assessing risk of bias of individual studies (including specification of whether this was done at the study or outcome level), and how this information is to be used in any data synthesis.	Page 15
Summary measures	13	State the principal summary measures (e.g., risk ratio, difference in means).	Page 16
Synthesis of results	14	Describe the methods of handling data and combining results of studies, if done, including measures of consistency (e.g., I^2) for each meta-analysis.	Page 16

Section/topic	#	Checklist item	Reported on page #
Risk of bias across studies	15	Specify any assessment of risk of bias that may affect the cumulative evidence (e.g., publication bias, selective reporting within studies).	Page 15
Additional analyses	16	Describe methods of additional analyses (e.g., sensitivity or subgroup analyses, meta-regression), if done, indicating which were pre-specified.	Page 16
**RESULTS**			
Study selection	17	Give numbers of studies screened, assessed for eligibility, and included in the review, with reasons for exclusions at each stage, ideally with a flow diagram.	n/a
Study characteristics	18	For each study, present characteristics for which data were extracted (e.g., study size, PICOS, follow-up period) and provide the citations.	n/a
Risk of bias within studies	19	Present data on risk of bias of each study and, if available, any outcome level assessment (see item 12).	n/a
Results of individual studies	20	For all outcomes considered (benefits or harms), present, for each study: (a) simple summary data for each intervention group (b) effect estimates and confidence intervals, ideally with a forest plot.	n/a
Synthesis of results	21	Present results of each meta-analysis done, including confidence intervals and measures of consistency.	n/a
Risk of bias across studies	22	Present results of any assessment of risk of bias across studies (see Item 15).	n/a
Additional analysis	23	Give results of additional analyses, if done (e.g., sensitivity or subgroup analyses, meta-regression [see Item 16]).	n/a
**DISCUSSION**			

For peer review only

Summary of evidence	24	Summarize the main findings including the strength of evidence for each main outcome; consider their relevance to key groups (e.g., healthcare providers, users, and policy makers).	Pages 17-18
Limitations	25	Discuss limitations at study and outcome level (e.g., risk of bias), and at review-level (e.g., incomplete retrieval of identified research, reporting bias).	Pages 18
Conclusions	26	Provide a general interpretation of the results in the context of other evidence, and implications for future research.	Page 18
**FUNDING**			
Funding	27	Describe sources of funding for the systematic review and other support (e.g., supply of data); role of funders for the systematic review.	n/a

From: Moher D, Liberati A, Tetzlaff J, Altman DG, The PRISMA Group (2009). Preferred Reporting Items for Systematic Reviews and Meta-Analyses: The PRISMA Statement. PLoS Med 6(7): e1000097. doi:10.1371/journal.pmed1000097

For more information, visit: **www.prisma-statement.org**.

Page 2 of 2

For peer review only

BMJ Open

The prevalence of child maltreatment in India and its association with gender, urbanisation and policy: a rapid review and meta-analysis protocol

Journal:	*BMJ Open*
Manuscript ID	bmjopen-2020-044983.R2
Article Type:	Protocol
Date Submitted by the Author:	23-Jul-2021
Complete List of Authors:	Fernandes, Gwen; University of Bristol, Population Health Sciences<br>Fernandes, Megan; Norfolk and Norwich University Hospital NHS Trust, Department of Surgery<br>Vaidya , Nilakshi ; National Institute of Mental Health and Neuro Sciences<br>De Souza, Philip; Norfolk and Norwich University Hospital NHS Trust, Department of Medicine<br>Plotnikova, Evgeniya; The University of Edinburgh Usher Institute of Population Health Sciences and Informatics<br>Geddes, Rosemary; The University of Edinburgh Usher Institute of Population Health Sciences and Informatics<br>Holla, Bharath; National Institute of Mental Health and Neurosciences<br>Sharma, Eesha ; National Institute of Mental Health and Neuro Sciences, Child And Adolescent Psychiatry<br>Benegal, Vivek; National Institute of Mental Health and Neurosciences<br>Choudhry , Vikas; Sambodhi Research and Communications Pvt Ltd., Public Health
**Primary Subject Heading**:	Paediatrics
Secondary Subject Heading:	Epidemiology
Keywords:	EPIDEMIOLOGY, Protocols & guidelines < HEALTH SERVICES ADMINISTRATION & MANAGEMENT, MENTAL HEALTH, PUBLIC HEALTH, Child protection < PAEDIATRICS

SCHOLARONE™
Manuscripts

I, the Submitting Author has the right to grant and does grant on behalf of all authors of the Work (as defined in the below author licence), an exclusive licence and/or a non-exclusive licence for contributions from authors who are: i) UK Crown employees; ii) where BMJ has agreed a CC-BY licence shall apply, and/or iii) in accordance with the terms applicable for US Federal Government officers or employees acting as part of their official duties; on a worldwide, perpetual, irrevocable, royalty-free basis to BMJ Publishing Group Ltd ("BMJ") its licensees and where the relevant Journal is co-owned by BMJ to the co-owners of the Journal, to publish the Work in this journal and any other BMJ products and to exploit all rights, as set out in our licence.

The Submitting Author accepts and understands that any supply made under these terms is made by BMJ to the Submitting Author unless you are acting as an employee on behalf of your employer or a postgraduate student of an affiliated institution which is paying any applicable article publishing charge ("APC") for Open Access articles. Where the Submitting Author wishes to make the Work available on an Open Access basis (and intends to pay the relevant APC), the terms of reuse of such Open Access shall be governed by a Creative Commons licence – details of these licences and which Creative Commons licence will apply to this Work are set out in our licence referred to above.

Other than as permitted in any relevant BMJ Author's Self Archiving Policies, I confirm this Work has not been accepted for publication elsewhere, is not being considered for publication elsewhere and does not duplicate material already published. I confirm all authors consent to publication of this Work and authorise the granting of this licence.

For peer review only

Rapid Review Protocol Paper

The prevalence of child maltreatment in India and its association with gender, urbanisation and policy: a rapid review and meta-analysis protocol

Gwen Sascha Fernandes[*1], Megan Fernandes*[*2], Nilakshi Vaidya[3], Philip De Souza[4], Evgeniya Plotnikova[5], Rosemary Geddes[5], Bharath Holla[3], Eesha Sharma[3], Vivek Benegal[3], Vikas Choudhry[6]*
*denotes joint first authorship

1. Population Health Sciences, Bristol Medical School, University of Bristol, UK
2. Department of General Surgery, Norfolk and Norwich University Hospital, Norwich, UK
3. The National Institute of Mental Health and Neurosciences, Bangalore, India
4. Department of Medicine, Norfolk and Norwich University Hospital, Norwich, UK
5. Usher Institute, School of Medicine, University of Edinburgh, UK
6. Sambodhi Research and Communications Pvt Ltd

Correspondence to:
Dr Gwen Fernandes; gwen.fernandes@bristol.ac.uk
Population Health Sciences
Bristol Medical School,
Barley House
University of Bristol
BS8 2BN

For peer review only

Abstract

Introduction: India is home to 20% of the world's children and yet, little is known on the magnitude and trends of child maltreatment nationwide. The aims of this review are to provide a prevalence of child maltreatment in India with considerations for any effects of gender; urbanisation (e.g. urban versus rural); and, legislation (Protection of Children from Sexual Offences Act 2012).

Methods and Analysis: A rapid review will be undertaken of all quantitative peer-reviewed studies on child maltreatment in India between 2005 and 2020. Four electronic databases will be systematically searched: PubMed, EMBASE, Cochrane and PsychInfo. The primary outcomes will include all aspects of child maltreatment: physical abuse, sexual abuse, emotional abuse, emotional neglect and physical neglect. Study participants will be between 0-18 years and will have reported maltreatment experiences using validated, reliable tools such as the Adverse Childhood Experiences Questionnaire as well as child self-reports and clinician reports. Study selection will follow the Preferred Reporting Items for Systematic Reviews and Meta-Analyses guidelines, and the methodological appraisal of the studies will be assessed by the Newcastle-Ottawa Quality assessment scale. A narrative synthesis will be conducted for all included studies. Also, if sufficient data are available, a meta-analysis will be conducted. Effect sizes will be determined from random-effects models stratified by gender, urbanisation and pre-and post the 2012 POCSO Act cut off. I^2 statistics will be used to assess heterogeneity and identify their potential sources and tau^2 statistics will indicate any between-study variance.

Ethics and Dissemination: As this is a rapid review, minimal ethical risks are expected. The protocol and Level 1 self-audit checklist was submitted and approved by the Usher Research Ethics Group (UREG) panel in the Usher Institute (School of Medicine and Veterinary Sciences) at the University of Edinburgh [Reference B126255]. Findings from this review will be disseminated widely through peer-reviewed publication and in various media, for example, conferences, congresses or symposia.

Registration: A study protocol was developed before starting this review and was registered with the international prospective register of systematic reviews (PROSPERO) (registration number CRD42019150403)

Strengths and Limitations

- This is the first rapid review of the peer-reviewed and published evidence on child maltreatment in India drawing on studies from four electronic databases: PubMed, EMBASE, Cochrane and PsychInfo
- The primary outcome is any type of prospectively recalled child maltreatment including physical abuse and neglect, emotional abuse and neglect and sexual abuse.
- First study to evaluate the effects of the legislative Act pre- and post-2012. (Protection of Children from Sexual Offences Act 2012)
- The review is limited by the exclusion of the grey literature and non-English peer-reviewed publications due to the lack of resources and funding available to conduct the review.
- The review will include a narrative synthesis of non-governmental reports, media articles and other sources of grey literature to inform the findings.

For peer review only - http://bmjopen.bmj.com/site/about/guidelines.xhtml

Introduction

In the past two decades, significant progress has been made in reducing child deaths. UNICEF estimates that, worldwide, there were 12.6 million children who died prematurely before the age of five in 1990 and this has declined to 5.3 million in 2018 [1]. Of the children who do survive, 200 million do not reach their full potential due to a combination of family socioeconomic status, geographic location, ethnicity, disability or religious orientation [1]. It is every child's right to develop and thrive, not just survive. Balanced nutrition, good healthcare and consistent care and encouragement in supportive home and school environments and positive neighbourhood environments enable children to lead healthy lives [2]. The early years of childhood therefore provide the basis of intelligence and learning, personality and self-motivation, social behaviour and collaboration, which extend into adulthood [3].

The World Health Organization (WHO) defines child maltreatment as 'the abuse and neglect that occurs to children under 18 years of age and includes all types of physical and/or emotional ill-treatment, sexual abuse, and neglect which results in harm'. The WHO reports that worldwide, child maltreatment is widespread with 1 in 4 adults reporting being physically abused as children [4,5]. Research suggests that children who have experienced such adversity do not receive the right nutrition, care or opportunities to learn and grow as children in more affluent geographical areas or economies [6,7]. However, these findings are not generalisable to lower-and middle-income countries (LMICs) like India where millions of children may be exposed to other, specific adversities such as conflict, political instability, community violence and other traumatic life experiences [7,8]. Research on early childhood adversity and consequences have been limited in India, including a lack of cross-sectional and longitudinal studies [9]. These adversities such as abuse or neglect, along with associated habits such as underage smoking or drinking, formed in childhood, have been linked to a range of poor physical and mental health outcomes throughout the life course and ultimately greater premature mortality [10]. These adversities and consequent health inequalities can be exacerbated by socioeconomic status and poverty, urbanisation (rural areas or slum areas compared with urban zones) or even caste [11].

In her book, Bitter Chocolate [12], Pinki Virani discusses the context of these adversities in India – a highly patriarchal culture in which reprimanding, punishing or spanking a child (physically abusive behaviour) is a cultural norm [13]. The Indian economy is further characterised by external adversities from a rapidly growing economy, stark urban-rural divides, religious strife and geopolitical upheavals [14]. Data from UNICEF and the World Bank [15] shows a global perspective of child rights and Indian children are marked as experiencing a difficult situation as categorised by their rights, access to education and healthcare and exposure to child labour and child marriages (Figure 1). It is no surprise that physiological and neurobiological studies have found that adversities such as child maltreatment can lead to changes of neural networks, impaired nervous, endocrine and immune systems development, resulting in physical, mental and emotional dysfunction and chronic physiological damage [16-18].

Figure 1. World map showing the rights of a child as measured by UNICEF and World Bank data [15].

For peer review only

An Indian context

In India, home to 19% of the world's children, it is estimated every second child is exposed to sexual abuse and violence [19]. The Indian National Crimes Records Bureau (NCRB) report a child is sexually abused every 15 minutes and 53% of children report abuse by a parent, relative or schoolteacher [20]. The prevalence of child sexual abuse (CSA) in high-income countries is 20% for women and 8% for men [21] but in India, the estimates vary between 4-66% for women and 4-57% for men [22,23,24]. These figures are considered underestimates because of limited surveillance of direct childhood adversity such as physical abuse or emotional abuse as well as CSA due to stigma particularly affecting girls; underreporting of cases by healthcare and police authorities; stigma and taboo associated with being a victim or experiencing abuse, and varying prevalence rates by geography (e.g. urban versus rural versus slum settings) as well as varying communities (school-based, hospital based, trafficking victims etc) [24-27].

Poverty, and especially urban poverty in India is prevalent due to high economic migration that has translocated poverty from rural settings to urbanised areas [28]. Whilst the proportion of deprived populations in India dropped from 45% to 22% between 1994 and 2012, one in every six urban Indians live in slums [29]. Health inequalities that exist as a result of poverty and rapid urbanisations need to be reduced so that child maltreatment, violence against girls and women, aims of the United Nations Sustainable Development Goals can be achieved. Bywaters *et al.,* [30] report strong associations between the socioeconomic status of a family and the risk of children within the household experiencing abuse and neglect. The greater economic hardship in areas like urban slums, the greater the likelihood and severity of child maltreatment. By contrast, we see evidence of the reverse in Deb and Modak's [31] study where violence is proportional to economic status as well as cultural beliefs and practices. Their data on socioeconomic status found that children from high income households were almost four times more vulnerable to physical violence compared to children from low-income households. This is attributed to parents within high income households having high expectations of their children especially with regards to academic achievement and a general undermining of a child's status compared to elder perspectives [32]. We see the nuanced effects of economics and urbanisation as children from more deprived backgrounds have parents who remain indifferent to their care and well-being especially for female children [33]. Given these findings, this review will carefully consider socioeconomic status of families, urbanisation metrics and specifically, the types of maltreatment affecting each sex within these settings.

Previous research has demonstrated that experiences of child maltreatment rarely occur in isolation and in fact, have a tendency to cluster [9]. Community-based studies have reported the prevalence and overlap of childhood sexual, physical and emotional abuse, but in India this has been limited. Choudhry's [22] systematic review focussed on one aspect of child maltreatment: CSA, and was seminal work in detailing the high prevalence rates of CSA in Indian children. However, the authors acknowledged that CSA does not occur in silos and often co-occurs with other forms of child maltreatment in the same child [34].

Childhood Adversity in India

For peer review only - http://bmjopen.bmj.com/site/about/guidelines.xhtml

Over the past 20 years, the phrase ‘adverse childhood experiences’ or (ACEs) have been studied as forms of maltreatment and household dysfunction affecting children either directly or indirectly. Feliiti and colleagues [35] found a strong graded relationship between exposure to abuse or dysfunction during childhood and multiple risk factors for mortality in adulthood. ACEs have been associated with non-communicable conditions such as cardiovascular disease, cancer or mental illness [7, 36-38].

Whilst alternative discourse on ACEs exists [39], there are limitations in the application of this ACEs framework including different types of exposures, lack of consideration for frequency or severity of occurrence and the relevance to population level, rather than individual level, policies. Also, the application of this framework within LMICs like India may be limited given the effects other adversities such as poverty, community violence and political strife [40].

Using Felitti’s ACEs framework and extending it with additional adversities [40], Figure 2 depicts different types of adversities each bearing significant ramifications extending from childhood to adulthood and beyond – affecting the family and society as a whole [41]. However, in India, the data on child maltreatment across different different states and settings is available but the data on ACEs is limited. A few recent studies have been able to identify the prevalence of ACEs in specific communities [22]. Damodaran and Paul[9] compiled data on ACEs and specifically, child maltreatment in youth based in Kerala (South India) and reported that in a sample of 600 young people, 91% had reported at least one ACE and over half the population reported three or more ACEs. However, in terms of severity, males reported exposure to more severe child maltreatment than females such as physical and sexual abuse

Figure 2. Adverse Childhood Experiences (ACEs) including traditional direct ACEs (child maltreatment: physical, sexual and emotional abuse and neglect) and indirect ACEs (Felitti et al., 1998) and further extended by researchers since the original study (Houtepen et al., 2018)

In India, lower incomes, poor living conditions and living with domestic violence are adversities that can lead to neglect and abuse and often ‘cluster’ [42]. Figure 3 combines the socioecological model of child maltreatment [43] and suggests potential pathways (e.g. from a macrosystem/societal or exosystem/community level) that may contribute to child maltreatment [[44-45]. Particularly, around the individual level factors – there is a gap in the rates of other types of child maltreatment associated with CSA (e.g. emotional abuse or neglect). Whilst the topic of ACEs in India has received limited attention, child sexual abuse (CSA) has received considerable attention from academics, clinicians, policymakers and legislators in recent years [22,46]. With the high prevalence of all types of child abuse in India, an attempt to amalgamate and compile the findings and present these figures in a systematic, cohesive and coherent manner is pressing [5].

Figure 3. Potential pathways explaining child maltreatment using the socioecological model proposed by Sidebotham and Heron [45]

For peer review only - http://bmjopen.bmj.com/site/about/guidelines.xhtml

Effect of Gender

Previous research has suggested the nuanced effect of gender on the occurrence of child maltreatment in India [22]. In India, gender identification is illegal and yet, almost half of girls 'wish they were born boys' [47]. Although anecdotal evidence and case reports suggest that prevalence of sexual abuse is higher in female children than in male children, based on hospital services or care services utilisation [48], some studies shows equal rates of sexual abuse across gender [47,49]. Deb and colleagues [50] suggest that boys may be experiencing and reporting more sexual abuse whilst girls are less likely to be aware of abuse (knowledge and attitudes) or less likely to report abuse for fear of shame or stigma. Society is more dismissive of male abuse than that of girls as patriarchal tendencies to 'protect' include girls, but do not extend to boys [49]. However, when it comes to neglect with regards to food and education security, girls are known to be disadvantaged [51]. India is the only country where girls have greater rates of under-five mortality compared with boys. Indian girls are breastfed for shorter periods and consume less milk (ibid). The reasons for this discrepancy are varied: there is a preference for sons over daughters in India and as an extension, more opportunities with regards to food, healthcare and education are afforded to male children [52]. However, given the complexities of gender effects in different types of abuse, generalisations cannot be drawn due to the limited number of studies, methodological limitations (e.g. small sample sizes, non-generalisability of findings to the wider Indian diaspora), urbanisation and socioeconomic privilege (e.g. remote tribal areas being worse affected than wealthier urban ones [53], and reporting bias (e.g. particularly if parents reported via food diaries what was consumed).

Effect of Urbanisation

The prevalence of child maltreatment by urbanisation (rural, slum or urban areas) has received limited attention in the evidence base. Only two studies [54,55] included community populations from rural areas as well as urban areas. Whilst other cross-sectional studies have been conducted in urban areas of India [56], the child maltreatment prevalence figures (32% of boys and 42% of girls) are higher but not comparable due to differences in sample size; socioeconomic status; access to healthcare and support systems in urban areas compared to rural areas, as well as differing definitions of child maltreatment.

Banerjee [57] discussed that child maltreatment was more prevalent in metropolitan cities than in rural areas of India and their study found a high frequency of physical abuse among the slum and pavement dwellers although the severity of maltreatment was low. The study highlighted child labour practices (with 78.4% of children between the ages of 4-5 years working part time jobs) as a result of living in a deprived area of the city. This study also highlighted the fact that often child maltreatment is a product of dire economic distress and within this context; small-scale regional or local studies might help understand the prevalence of child maltreatment in specific areas by urbanisation metrics. Over half the children who were classed as homeless (i.e. living in drainage pipes, under tarpaulins, flyovers and those living in the open) reported CSA [58]. While child maltreatment figures such as CSA may seem high in urban or slum areas, the disadvantage with rural areas may be that abuse may go under the radar. They may be under-reported or disguised within local traditions such as a dowry culture or child marriages, all behaviours rooted in poverty, and volatile socioeconomic circumstances [59].

For peer review only - http://bmjopen.bmj.com/site/about/guidelines.xhtml

Effect of POCSO Act

The Protection of Children from Sexual Offences Act (POCSO Act) was established in 2012 by the Indian government to protect children against offences like sexual abuse. The Act makes it mandatory to report acts of sexual offence and document reporting timelines, prosecution timelines and clearly record all evidence (within a 30-day period). Whilst POSCO has been a relatively recent Act, it's effect on numbers of reported offences has been debated [22]. Whilst the gold standard remains as a multi-discipinary, multi-faceted and holistic team providing psychological and legal support to the child and their families, this level of comprehensive care *under one roof* is yet to exist in India [60.61]. The impact of this study on policymaking will come from its inclusion of the only legislative act in India that focuses on child sexual abuse. Given the overlap between different types of abuse and neglect and their tendency to 'cluster', we will determine any effects of POCSO pre- and post-2012 on all forms of child maltreatment. This has not been evaluated by the literature to date but was highlighted for future work by Choudhry and colleagues [22].

Rationale

There are known gaps in the evidence base with regards to the overlap of different forms of child maltreatment and specifically, the gendered effects of child maltreatment. These include the identification and motivation of perpetrators and what happens after maltreatment has happened – a child's awareness of their rights and attitudes towards abuse and neglect and reporting of child maltreatment. The circumstances that encourage reporting and details on protective factors and risk factors of child maltreatment have received limited attention in the literature [22].

This review will build on these thematic and methodological gaps in the literature, synthesise the evidence by gender, urbanisations and POCSO effects and provide practice, policy and clinical solutions that may help reduce the occurrence and impact of child maltreatment in India.

Research Aims and Objectives

The primary research aim is to describe the prevalence of child maltreatment in India. The secondary aim is to determine the effect of the POCSO Act on child sexual abuse and on other forms of child maltreatment .

The specific objectives were: to determine the prevalence of child maltreatment (physical abuse, sexual abuse, emotional abuse and physical and emotional neglect); to present rates by gender, urbanisation (rural, slum or urban) and study sample types (e.g. community, school-going, high-risk such as trafficking victims, etc); to determine any impact of the POCSO Act (by the date of implementation of the Act i.e. 2012) on rates; and, to identify implications for future policy, practice and research amendments.

For peer review only - http://bmjopen.bmj.com/site/about/guidelines.xhtml

Methods and Analysis

Study design

This review and meta-analysis will be carried out in compliance with the Cochrane Handbook for Systematic Reviews of Interventions (version 6.1) and reported in compliance with the Preferred Reporting Items for Systematic Reviews and Meta-Analyses (PRISMA) guidelines [62,63,64].

Patient and Public Involvement

It was not appropriate or possible to involve patients or the public in the design, conduct, reporting or dissemination plans of our research. The research team and advisory/expert panel consisted of clinicians, policymakers and research academics who work in the area of early childhood adversity and were able to advise on search terms used; databases selected and dissemination plans for the work on completion.

Participants

Inclusion criteria

Studies between 1st January 2005 to 1st January 2020 will be included to allow a comparison of an 8-year window pre- and post- 2012 POCSO Act. Studies with participants aged between 0-18 years will be included in the review; and will have experienced any form of child maltreatment as determined using ISPCAN Child Abuse Screening Tool (ICSAT)-C, the Childhood Trauma Questionnaire, Adverse Childhood Experiences International Questionnaires (ACEIQ) or self-reports or clinician reports. ACEIQ or WHO definitions will also be used to determine child maltreatment [65] in addition to those by Day *et al.,* [66]. The primary outcome measure is any type of child maltreatment: sexual, physical or emotional abuse; and physical and emotional neglect. In addition to the definition, the time frame of recall, prospective recall in our case, warrants discussion.

Often, prospective and retrospective measures of child maltreatment do not always identify the same individuals [67]. Retrospective studies of child maltreatment are often regarded as a shortcut as significant time; finance and resources are not required to undertake prospective studies [68]. There is low agreement between prospective and retrospective assessments of child maltreatment that can be explained by factors such as motivation of reporting, measurement features and memory bias. Firstly, motivation can reduce agreement if individuals may gain something by intentionally withholding data about their maltreatment for embarrassment or fear or feeling uncomfortable or upset. Secondly, child maltreatment assessments are known to have imperfect test-retest reliability [69] and prospective measures might only account for the more severe of cases whilst retrospective 'true positive' case [67]. Lastly, memory bias can reduce agreement as a result of impaired brain development [70], deficit in memory consolidation of traumatic events or disrupted sleep patterns [71]. Therefore, given the variation, the focus on prospective recall (rather than retrospective) of child maltreatment for systematically reviewing the literature on abuse and neglect in children would reduce variation errors, measurement bias and memory bias [67].

Exclusion criteria

Studies that are qualitative in nature or mixed-methods (due to the time factor of this review), non-Indian study contexts, non-English studies, retrospective studies of adult CSA survivors, studies with insufficient peer-reviewed published data, media

For peer review only

reports, newspaper articles and non-peer reviewed articles will be excluded from this review. Only quantitative studies published in the English Language will be included.

Outcomes

Defining Childhood Maltreatment

The WHO defines child maltreatment as *'the abuse and neglect that occurs to children under 18 years of age and includes all types of physical and/or emotional ill-treatment, sexual abuse, neglect which results in harm'.* The National Society for the Prevention of Cruelty to Children (NSPCC) defines child maltreatment as harm experienced by a child by an adult or child *that is physical, sexual or emotional but can also involve a lack of love, care and attention* [72]. The NSPCC also set out clear descriptions of what constitutes child maltreatment and specifically each form of abuse and neglect that are summarised here:

Child Physical Abuse (CPA)

Physical abuse happens when a child is hurt or harmed deliberately causing injuries such as bruises, burns, cuts and broken bones. It may also include children being administered medication they do not need or not being given essential medicines for illness. The terms used specifically are kicking, shaking, slapping, throwing, poisoning or drowning. In India, these terms and additional ones such as 'spanking', 'rap' or 'pasting' also have equivalent meanings.

Child Sexual Abuse (CSA)

Sexual abuse happens when a child is forced or tricked into sexual activities. It happens when an abused makes physical contact with a child and includes touching, kissing and oral sex or non-contact abuse which includes exposing or flashing, exposing a child to sex acts or making, viewing or distributing child abuse images or videos.

Child Emotional Abuse (CEA)

Emotional abuse includes acts that humiliate a child by constantly criticising them, insulting or shouting at them, blaming and making them a scapegoat or manipulating a child. These behaviours also include not allowing them to have friends or never saying anything kind or positive or congratulatory to a child. It perpetuates the feelings that a child is not loved, is worthless or inadequate.

Child Physical and Emotional Neglect (CPEN)

Neglect is the on-going failure to meet a child's basic requirements and is often also classed as a form of child abuse. Physical neglect is when a child's basic needs such as food, clothing or shelter are not met or they are not properly supervised or kept safe. Emotional neglect is when a child is not provided with the nurture and stimulation they need to grow and thrive.

Whilst child maltreatment is a global problem, it is certainly difficult to assess and manage in LMICs like India. Specific types of maltreatment such as abandonment, child labour, street-begging and corporal punishment are highly prevalent [73]. Therefore, while generic methods may exist to assess child maltreatment, the value of self-reports from children or from paediatricians, child psychologists or social workers and the police should not be dismissed.

For peer review only - http://bmjopen.bmj.com/site/about/guidelines.xhtml

Search Strategy

The relevant search terms to be included in the search strategy will be identified during a preliminary search reported in the study protocol using PubMed's medical subject heading's (MeSH) browser. The search contained both free text terms and controlled vocabulary like MeSH terms as well as truncation and Boolean operators. In order to improve the quality of the search strategy and avoid bias or errors, the strategy includes choosing all appropriate terms, both descriptors and synonyms including those that may be specific to the Indian diaspora [74]. Firstly, the initial search terms were developed with reference to seminal works or major publications of child maltreatment and early childhood adversity in India [9, 26, 47,75]. The review then used the MeSH terms and keywords documented (Table 1) and were further enhanced by the search strategy from a systematic review conducted on child sexual abuse in Indian children [22]. These were further validated with input from an expert advisory panel in the field of systematic reviews and ACEs in India (Appendix 1). Five electronic databases will be systematically searched: PubMed, EMBASE, Cochrane and PsychInfo and Campbell Library. PubMed and EMBASE are the two largest healthcare bibliographic databases containing the vast majority of published health-related literature [74,76]. Searching Cochrane and PsychInfo will provide nuanced searches of other systematic reviews of child maltreatment or ACEs from the Asian continent and specifically from India. Systematic reviews will be included in the search to identify potentially relevant studies that may have been excluded by the electronic search strategy. Appendix 2a-2d contains the search strategies used in the four databases respectively. These search strategies and advice on specific electronic databases will be cross-checked and verified by an information specialist. The final search strategy used will be published in detail in the future published manuscript of results.

**Sexual Abuse**	**Sexual Abuse (cont)**	**Physical Abuse**	**Emotional Abuse**	**Emotional Neglect**	**Additional Child Maltreatment definitions**
Child* sexual abuse	Child abuse survivor	Child Hitting	Child worthless	Denial clothing	Child neglect
Child sexual assault	Child sexual offender	Child Shaking	Child unloved	Denial shelter	Child psychological assault
Child sexual coercion	Child pornography	Child Throwing	Child inadequate	Denial food	Child denial
Child rape	Child molestation	Child Poisoning	Child failure	Carer failure	Child pasting
Child Prostitution	Child sexual crime	Child Burning	Child unloved	Parental failure	Child emotional assault
Child Crime victims	Child physical	Child Scalding	Child corruption	Emotional harm	Non-accidental injury
Child Incest	Child assault	Child Drowning	Child unheard	Emotional danger	
Child Perpetrator / Child victim 3	Child beating	Child Suffocating	Cruelty	Child* neglect	
Child Physical violence	Child emo* abuse			Abandonment	
Child Mental Violence	Child maltreat*			Bereavement	

For peer review only - http://bmjopen.bmj.com/site/about/guidelines.xhtml

Child Sexual Violence	Child sexual coercion			Grief	
Child sexual offence	Child sexual aggression				

Table 1. Search term strategies using keywords and MeSH terms ("child abuse, sexual"[MeSH Terms] OR ("child"[All Fields] AND "abuse"[All Fields] AND "sexual"[All Fields]) OR "sexual child abuse"[All Fields] OR "physical child abuse" [All Fields] OR "emotional child abuse"[All Fields] OR ("child"[All Fields] AND "sexual"[All Fields] AND "abuse"[All Fields]) OR "child sexual abuse"[All Fields]) AND ("india"[MeSH Terms] OR "india"[All Fields]) - expanded from Choudhry *et al.,* (2018) and Day *et al.,* 2008 and updated to include child maltreatment definitions and **alternative search terms to include adolescent sexual abuse, adolescent physical assault, etc.*

Data Management

The selection of studies will be carried out systematically by two people independently, and in accordance with PRISMA guidelines, to improve accuracy and to minimise bias [63]. The first reviewer (first author) is an epidemiologist with expertise in early childhood adversities and the impact on health outcomes; the secondary reviewer is a clinical psychologist with expertise on ACEs in India whilst the third and fourth reviewers, are clinical doctors with expertise in general medicine and paediatric care, validated the selection of studies pre-and post-2012 threshold.

All studies identified from the individual search engines will be imported into a reference manager, EndNote, which will be used for de-duplication. The references will then be extracted into Rayyan for management between the first author and secondary reviewers [77]. All abstracts and full text articles will be reviewed independently by the first author and secondary reviewers as a fixed percentage of the work (40%). The details of all articles processed (including duplicates and details of excluded texts at each screening stage) as well as reasons for inclusion and exclusion from the study will be recorded and logged for the first author and secondary reviewers and presented in a PRISMA flowchart. At the title/abstract screening stage, the authors will use Rayyan's coding system (traffic light system) with appropriate tags such as 'wrong outcome of interest' in order to code each study (done independently by the authors on Rayyan's blinded review setting). Once all titles/abstracts are screened, we will unblind all authors and discuss those titles/abstract to be included, or excluded or further discussed with the expert panel as a result of discrepancies [77].

All selected full-text articles will be screened by the primary author and secondary reviewers and the reasons for exclusion of these articles will be recorded and presented in the final manuscript.

Data Extraction

The following data will be extracted from the included studies: title; author information; year of study (not publication date); study design; recruitment strategy and sample characteristics (school-based, community, hospital based, etc); sample size; urbanisation metrics (urban or rural or slum); response rate; definition of child maltreatment used; timeframe of child maltreatment (in years e.g. past one year or past 6 months or half a year); mean age of participants; gender distribution; statistical methodology; confounder data including parental relationships, home ownership, family settings (e.g. nuclear, joint, mixed), religion, educational status and literacy rates; prevalence rate of each type of child maltreatment; author's key message or conclusions; ethical approval and any conflicts of interest. An example of a completed data extraction form is presented in the following Table 2 [26].

For peer review only - http://bmjopen.bmj.com/site/about/guidelines.xhtml

Title of Study<br><br>Authors<br><br>Year of Study	Prevalence of child abuse in Kerala, India: An ICAST-CH based survey<br><br>Manoj Therayil Kumar, Nilamadhab Kar, Sebind Kumar<br><br>2019s
Study Design	Cross-sectional survey in Thrissur, Kerala
Recruitment Strategy	There are 39 schools in Thrissur with 15,150 students in attendance. Random number generation in excel was used to select half the schools on the list and all students in grades 8-10 who attended school on that day were eligible to take part.
Sample setting (school/college, clinical, community), Sample size and response rate	School setting.<br>6957 participants took part in the survey. No details of response rate were provided however the authors state that no questionnaire was returned completely blank.
Child maltreatment definition used (timeframe defined)	All abuse-related questions in the ICAST-CH were scored on a severity scale and categorised into both one year prevalence and lifetime prevalence.
Classification tool<br><br>Validity/Reliability of tool	The International Society for the Prevention of Chlld Abuse and Neglect (IPSCAN) Child Abuse Screening Tool – Child Home Version (ICAST-CH) was used.<br><br>Developed in unison with the WHO and UNICEF and has good criterion validity and was translated into Malayalam and was piloted for use before being administered to the students. Internal consistency measures using Cronbach's alpha for subscales – exposure to violence, physical abuse, emotional abuse, neglect and sexual abuse.
Urban/Rural/Slum-dwelling participants breakdown	Not mentioned
Mean Age (SD)	13.9 years (1.12) for males and 13.7 years (0.99) for females
Gender distribution	2071 males (30.1%)<br>4810 females (69.9%)
Example: CSA prevalence by gender	30.9% in males for one year prevalence<br>10.8% in females for one year prevalence<br><br>34.2% in males for lifetime prevalence<br>14% in females for lifetime prevalence
Statistical Methods used	Frequency and counts used to determine prevalence and presented with 95% confidence intervals<br>Cronbach alpha used to determine internal consistency of the Malayalam version of the scale

For peer review only - http://bmjopen.bmj.com/site/about/guidelines.xhtml

	Significance was set at the 0.05 level as a maximum but 0.01 and <0.001 was reported.
Confounders measured and/or adjusted for	Measured confounders – gender, age, religion, family type, accommodation, parental accommodation and socioeconomic status.
Other confounders discussed but not measured	Literacy rates, cultural strata of participants
Participants experience of:<br><br>Emotional abuse (rate, %)<br>Physical Abuse (rate, %)<br>Sexual Abuse (rate, %)<br>Emotional Neglect (rate, %)<br>Physical Neglect (rate, %)	
Conclusion/Comments	The abuse reported were more occasional experiences rather than frequent reporting nonetheless as 89.9% of any abuse in the past one year and 91% in the lifetime. First study in Indian diaspora to use the ICAST-CH tool to estimate household abuse. Did not include external environments such as schools.
Conflicts of Interest<br><br>Ethical Approval	None declared<br><br>Ethical approval sought from local authority and educational ministry to conduct the research.
Funders	Municipal Corporation of Thrissur city

Table 2. Example of a data extraction form

Quality Assessment

The final selected studies will be appraised for quality by the first author and, to ensure robustness, they will also be quality appraised by a second reviewer. A third reviewer will resolve any conflicts, with support from the expert advisory panel if required.
Study quality (largely cross-sectional and case-control) will be assessed using the Newcastle-Ottawa Quality Assessment Scale (NOS) adapted for use in cross-sectional study designs as it considers key areas: selection (representativeness of the sample, sample size, non-responders and exposure details); comparability (is confounding considered) and, outcome (blinding, recording and statistical test used) [78,79]. Whilst there are a myriad range of assessment tools available, the NOS has been endorsed by the Cochrane Collaboration to assess quality of research studies [76]. An example of a NOS assessment of a sample study is presented in Appendix 3.

Data synthesis

The study will include a narrative synthesis of the findings and attempt a meta-analysis dependant on heterogeneity. A meta-analytic approach will be used including any random-effect pooled prevalence of child maltreatment stratified by gender, urbanisation and pre-and post-POCSO Act. Heterogeneity will be evaluated using forest plots and measuring the impact of the I^2 statistic (I^2 >50% indicates significant heterogeneity) and tested with the χ^2 statistic ($p<0.05$) [62]. We will also report tau^2 values to indicate any between-study variance. We will use a fixed effects model for meta-analysis, except where we identify statistical heterogeneity in which case, we

For peer review only - http://bmjopen.bmj.com/site/about/guidelines.xhtml

would use a random effects Poisson model [80] to determine an ‘average prevalence’ across the population of all included studies. We will use the ‘metaprop’ command to perform meta-analyses of proportions of child maltreatment in STATA, which builds on the ‘metan’ command [81] but is typically used to pool effects such as proportions from cross-sectional studies instead of odds ratios from case-control studies.

In the case of significant heterogeneity derived from our analysis, we will attempt to create subgroups of the paediatric population to understand any extracted data. This could include different age-bands of participants (e,g, child (0-12 years), adolescent (13-18 years) using WHO cut-offs for age bands in paediatric populations. We may also wish to compare prevalence rates of child maltreatment based on self-report measures versus objectively assessed measures as well as compare rates by time (one year compared with lifetime occurrence or frequency data).

Meta-bia(s)
Research papers written originally in Hindi or any other Indian dialect, that would otherwise be included in the data capture, will not be used for the purpose of this study.

For peer review only

Discussion

The findings of this study will aim to add to the limited knowledge of child maltreatment in India and specifically, the impact of gender, urbanisation and the POCSO Act on the prevalence and determinants of child maltreatment. There are known gaps in the evidence base with regards to the overlap of different forms of child maltreatment and specifically, the gendered effects of child maltreatment. These include the identification and motivation of perpetrators and what happens after maltreatment has happened – a child's awareness of their rights and attitudes towards abuse, neglect and reporting of child maltreatment. The circumstances that encourage reporting, details on protective factors and risk factors of child maltreatment have received limited attention in the literature [22].

Furthermore, the impact of child maltreatment and where they occur (e.g. urban areas versus slum areas) is important as these may offer an insight into potential interventional pathways that differ by geography e.g. enforcing child welfare policies in rural areas [22]. The impacts of urban and rural poverty, socioeconomic status; family income; parental occupation; family structure (nuclear, joint, extended, etc) and other confounder data will be collated during this review to aid in the interpretation of results including subgroup analysis and meta-analysis. Furthermore, the impact of this study on policymaking will come from its inclusion of the only legislative act in India that focusses on child maltreatment. The Protection of Children from Sexual Offences Act (POCSO Act) was established in 2012 by the Indian government to protect children against offences like sexual abuse. The Act makes it mandatory to report acts of sexual offence and document reporting timelines, prosecution timelines and clearly record all evidence (within a 30-day period). Whilst POSCO has been a relatively recent Act, its effect on numbers of reported offences in research studies has yet to be determined.

Implications on Policy and Research

When the evidence on child maltreatment in India is collated and presented, any policy or practice impacts can be tailor-made based on the country-specific context and findings. In India, the challenge will come from addressing cultural norms and traditional values (e.g. gender inequity and reducing child marriages), creating safer environments for children within homes and schools (e.g. authorities penalising assault) and scaling up response and support services for children in different settings such as tribal regions, slum areas, rural communities (e.g. trauma informed health and social care). This approach has worked in higher-income countries but in LMIC's like India, there are challenges. These include low levels of trauma-informed care for injured children in hospital settings with 91% of healthcare workers not receiving any trauma-informed training [82]. Other challenges included overburdened judicial and criminal systems; poor child welfare services; gender inequality and illiteracy [22]. This study will capture data on health inequities and social determinants that predispose to maltreatment in different geographical areas of India including confounders such as poverty and neglect.

Strengths and Limitations

There are some strengths to our approaches. This is the first review undertaken focussing on all aspects of child maltreatment in India where previous review articles

For peer review only - http://bmjopen.bmj.com/site/about/guidelines.xhtml

and studies have focussed on a specific facet of maltreatment, e.g. child sexual abuse. Secondly, the inclusion of the POCSO Act makes this review nuanced to comment on any effect of legislation where it has never been formally evaluated [83]. This study will include an analysis of child maltreatment studies and corresponding prevalence rates, both pre-and post-2012. With the inclusion of the POCSO 2012 threshold, it is expected that there will be an increased number of cases reporting all forms of child maltreatment. Thirdly, the authors will attempt to meta-analyse the data depending on heterogeneity scores generated. Choudhry's work [22] highlighted the significant heterogeneity of studies conducted to date and this might limit the quality of meta-analytics possible given the discrepancies in samples size. Possible reasons for heterogeneity include varying age ranges of participants, selection bias with sample provenance (e.g recruitment from observation homes vs. school children), varying urbanisation metrics (e.g. samples including children from all types of socioeconomic backgrounds) and variation in outcome assessment definition (e.g. self-report vs. validated questionnaire).

There are limitations to this review. Firstly, only papers in English were included due to limited access to funding and resources such as a professional translator. We acknowledge the importance of Anglophone bias in the context of this Indian based study therefore endeavour to ensure future extensions of this research does include non-English papers Secondly, excluding grey literature from OPEN Grey, narrative reports from non-governmental organisations or World Bank reports would limit some of the 'unmeasured' exposures and confounders that may increase child maltreatment [25, 49]. However, the authors will make attempts to collate this additional information so that they may be presented as part of a narrative synthesis on the topic of child maltreatment in India, thus improving the quality, generalisability and relevance of our work [84]. The exclusion of national statistics (e.g. NCRB records) might limit interpretation of any effect of the POCSO Act on conviction rates for child maltreatment. Qualitative studies were also excluded from this review which may not effect prevalence rates of child maltreatment but would allow exploration of non-responses from children (interpretative) or indeed, highlight determinants that have not been identified in quantitative or aggregated reviews [85-87].

This review will build on these thematic and methodological gaps in the literature, synthesise the evidence by gender, urbanisations and POCSO effects and provide practice, policy and clinical solutions that may help reduce the occurrence and impact of child maltreatment in India.

Ethics

A Level 1 self-audit checklist was approved by an ethics panels at the Usher Institute at the University of Edinburgh. Minimal ethical risks are expected. However, Vergnes *et al.,* [64] propose that for SRs particularly on sensitive topics authors should consider a minimal ethical assessment review and report these in their research studies. As a result, in this review, ethical approval details from each included study will be collected.

Dissemination

The findings of this review will be published in a peer-reviewed journal, and disseminated at national and international conferences and through social media.

For peer review only - http://bmjopen.bmj.com/site/about/guidelines.xhtml

Acknowledgements: The authors would like to extend their sincere gratitude to Mrs Sarah Herring, Subject Librarian for Population Health Sciences, University of Bristol for her advice and guidance during the protocol development stage.

Contributorship statement: GF, RG and EP were responsible for the initial protocol drafting and design. GF, MF, NV, PD then contributed to preliminary searches used to develop the rationale and background of the study. ES, BH, VC, VB, PD & MF were on the expert advisory panel for the study to advise on methods design and statistical analysis techniques that could be used. All authors contributed and approved this final manuscript.

Competing Interests: None to declare.

Funding: Dr Gwen Fernandes is funded by the UK Medical Research Council and Alcohol Research UK Grant (MR/L022206/1).

Ethics Statements: The protocol and Level 1 self-audit checklist was submitted and approved by the Usher Research Ethics Group (UREG) panel in the Usher Institute (School of Medicine and Veterinary Sciences) at the University of Edinburgh [Reference B126255]

Data Sharing Statement: Data sharing is not applicable to this article as no new data were created or analysed in this study.

For peer review only

References

1. WORLD HEALTH ORGANISATION, 2019. Children: reducing mortality. Available from: https://www.who.int/news-room/fact-sheets/detail/children-reducing-mortality [Accessed 2nd February 2020]

2. GRIFFITHS, S., HUNTER, DJ, 2007. New Perspective in Public Health. London: Radcliffe Publishing.

3. REYNOLDS, A.J., ROLNICK, A.J., TEMPLE, J.A., 2015. Health and Education in Early Childhood: Predictors, Interventions and Policies. Cambridge: Cambridge University Press.

4. WORLD HEALTH ORGANISATION 2017. Country Cooperation Strategy at a glance: India. Available from: https://apps.who.int/iris/bitstream/handle/10665/136895/ccsbrief_ind_en.pdf;jsessionid=90BA4E2E08E57D23C4363B8FBDFC85AC?sequence=1 [Accessed 5th March 2020]

5. WORLD HEALTH ORGANISATION, 2006. Preventing Child Maltreatment: a guide to taking action and generating evidence. Available from: https://apps.who.int/iris/bitstream/handle/10665/43499/9241594365_eng.pdf;jsessionid=555709D037DBAD5DE822D439283A5F1B?sequence=1 [Accessed 1st of February 2020]

6. BLUM, R. W., LI, M. & NARANJO-RIVERA, G. 2019. Measuring Adverse Child Experiences Among Young Adolescents Globally: Relationships With Depressive Symptoms and Violence Perpetration. *Journal of Adolescent Health,* 65**,** 86-93.

7. BELLIS, M. A., HUGHES, K., FORD, K., RAMOS RODRIGUEZ, G., SETHI, D. & PASSMORE, J. 2019. Life course health consequences and associated annual costs of adverse childhood experiences across Europe and North America: a systematic review and meta-analysis. *The Lancet Public Health,* 4**,** e517-e528.

8. SHARMA, E., VAIDYA, N., IYENGAR, U., ZHANG, Y., HOLLA, B., PURUSHOTTAM, M., CHAKRABARTI, A., FERNANDES, G. S., HERON, J., HICKMAN, M., DESRIVIERES, S., KARTIK, K., JACOB, P., RANGASWAMY, M., BHARATH, R. D., BARKER, G., ORFANOS, D. P., AHUJA, C., MURTHY, P., JAIN, S., VARGHESE, M., JAYARAJAN, D., KUMAR, K., THENNARASU, K., BASU, D., SUBODH, B. N., KURIYAN, R., KURPAD, S. S., KALYANRAM, K., KRISHNAVENI, G., KRISHNA, M., SINGH, R. L., SINGH, L. R., KALYANRAM, K., TOLEDANO, M., SCHUMANN, G., BENEGAL, V. & THE C, V. C. 2020. Consortium on Vulnerability to Externalizing Disorders and Addictions (cVEDA): A developmental cohort study protocol. *BMC Psychiatry,* 20**,** 2.

9. DAMODARAN, D. K. & PAUL, V. K. 2017. Patterning/Clustering of Adverse Childhood Experiences (ACEs): The Indian Scenario. *Psychological Studies,* 62**,** 75-84.

10. HUGHES, K., BELLIS, M. A., HARDCASTLE, K. A., SETHI, D., BUTCHART, A., MIKTON, C., JONES, L. & DUNNE, M. P. 2017. The effect of multiple adverse childhood experiences on health: a systematic review and meta-analysis. *The Lancet Public Health,* 2**,** e356-e366.

For peer review only - http://bmjopen.bmj.com/site/about/guidelines.xhtml

11. KOWAL, P. & AFSHAR, S. 2015. Health and the Indian caste system. *The Lancet,* 385**,** 415-416.

12. VIRANI, P., 2000. Bitter Chocolate. New Delhi: Penguin Books.

13. NIJHARA, K., BHATIA, S. & UNNIKRISHNAN, B. 2018. Corporal Punishment in Children and its Implications on Mental Health. *The Indian Journal of Pediatrics,* 85**,** 405-405.

14. KOCHANEK, S.A., and HARDGRAVE, R.L., 2008. India: Government and Politics in a Developing Nation. Boston: Thomson Higher Education.

15. UNICEF 2014. Ending violence against children in Asia. Available from: https://www.unicef.org/publications/files/Ending_Violence_Against_Children_Six_strategies_for_action_EN_9_Oct_2014.pdf [Accessed 24th Jan 2019]

16. ANDA, R. F., FELITTI, V. J., BREMNER, J. D., WALKER, J. D., WHITFIELD, C., PERRY, B. D., DUBE, S. R. & GILES, W. H. 2006. The enduring effects of abuse and related adverse experiences in childhood. *European Archives of Psychiatry and Clinical Neuroscience,* 256**,** 174-186.

17. PECHTEL, P. & PIZZAGALLI, D. A. 2011. Effects of early life stress on cognitive and affective function: an integrated review of human literature. *Psychopharmacology (Berl),* 214**,** 55-70.

18. DANESE, A. & MCEWEN, B. S. 2012. Adverse childhood experiences, allostasis, allostatic load, and age-related disease. *Physiol Behav,* 106**,** 29-39.

19. BEHERE, P. B., SATHYANARAYANA RAO, T. S. & MULMULE, A. N. 2013. Sexual abuse in women with special reference to children: Barriers, boundaries and beyond. *Indian journal of psychiatry,* 55**,** 316-319.

20. NATIONAL CRIME RECORDS BUREAU. 2016. Crime in India – Statistics. Available from: http://ncrb.gov.in/StatPublications/CII/CII2016/Press%20release-Crime%20in%20India%202016.pdf [Accessed 14th Feb 2019]

21. STOLTENBORGH, M., VAN IJZENDOORN, M. H., EUSER, E. M. & BAKERMANS-KRANENBURG, M. J. 2011. A global perspective on child sexual abuse: meta-analysis of prevalence around the world. *Child Maltreat,* 16**,** 79-101.

22. CHOUDHRY, V., DAYAL, R., PILLAI, D., KALOKHE, A. S., BEIER, K. & PATEL, V. 2018. Child sexual abuse in India: A systematic review. *PLoS One,* 13**,** e0205086.

23. WORLD HEALTH ORGANISATION, 2016. CHILD MALTREATMENT. Available from: https://www.who.int/news-room/fact-sheets/detail/child-maltreatment [Accessed 16th October 2019]

24. RADHIKA, K., MANJULA, M. & JAISOORYA, T. S. 2018. Ethical gaps in conducting research among adult survivors of child sexual abuse: a review. *Indian J Med Ethics,* 3**,** 186-192.

25. SETH, R., BOSE, V., QAIYUM, Y., CHANDRASHEKHAR, R., KANSAL, S., TANEJA, I. & SETH, T. 2018. Social Determinants of Child Marriage in Rural India. *Ochsner J,* 18**,** 390-394.

For peer review only - http://bmjopen.bmj.com/site/about/guidelines.xhtml

26. KUMAR, M. T., KAR, N. & KUMAR, S. 2019. Prevalence of child abuse in Kerala, India: An ICAST-CH based survey. *Child Abuse Negl,* 89**,** 87-98.

27. HAILES, H. P., YU, R., DANESE, A. & FAZEL, S. 2019. Long-term outcomes of childhood sexual abuse: an umbrella review. *The Lancet Psychiatry,* 6**,** 830-839.

28. DATT, G., RAVALLION, M. & MURGAI, R. 2020. Poverty and Growth in India over Six Decades. *American Journal of Agricultural Economics,* 102**,** 4-27.

29. World Bank Data. Population Living in Slums. Available from: https://data.worldbank.org/indicator/EN.POP.SLUM.UR.ZS?locations=IN [Accessed 5th February 2021]

30. BYWATERS, P, BUNTING, L, DAVIDSON G, HANRATTY J, MASON W, MCCARTAN C, AND STEILS, N, 2016. The relationship between poverty, child abuse and neglect: an evidence review. Available from: file:///C:/Users/gf18522/Downloads/bywaters_can_final_report.pdf [Accessed 6th June 2019]

31. DEB, S. & MODAK, S. 2010. Prevalence of violence against children in families in Tripura and its relationship with socio-economic factors. *J Inj Violence Res,* 2**,** 5-18.

32. BANDEWAR, S. V., PITRE, A. & LINGAM, L. 2018. Five years post Nirbhaya: Critical insights into the status of response to sexual assault. *Indian J Med Ethics,* 3**,** 215-221.

33. NATIONAL FAMILY HEALTH SURVEY. 2016. Available from: http://rchiips.org/nfhs/factsheet_NFHS-4.shtml [Accessed 16th Jan 2019]

34. CARSON, D.K., FOSTER, J.M., CHOWDHURY, A., 2014. A. Sexual Abuse of Children and Youth in India: An Anthropological Perspective. The Oriental Anthropologist, 14(2):343.

35. FELITTI, V. J., ANDA, R. F., NORDENBERG, D., WILLIAMSON, D. F., SPITZ, A. M., EDWARDS, V., KOSS, M. P. & MARKS, J. S. 1998. Relationship of childhood abuse and household dysfunction to many of the leading causes of death in adults. The Adverse Childhood Experiences (ACE) Study. *Am J Prev Med,* 14**,** 245-58.

36. SPRINGER, K. W., SHERIDAN, J., KUO, D. & CARNES, M. 2007. Long-term physical and mental health consequences of childhood physical abuse: results from a large population-based sample of men and women. *Child Abuse Negl,* 31**,** 517-30.

37. KESSLER, R. C., MCLAUGHLIN, K. A., GREEN, J. G., GRUBER, M. J., SAMPSON, N. A., ZASLAVSKY, A. M., AGUILAR-GAXIOLA, S., ALHAMZAWI, A. O., ALONSO, J., ANGERMEYER, M., BENJET, C., BROMET, E., CHATTERJI, S., DE GIROLAMO, G., DEMYTTENAERE, K., FAYYAD, J., FLORESCU, S., GAL, G., GUREJE, O., HARO, J. M., HU, C. Y., KARAM, E. G., KAWAKAMI, N., LEE, S., LEPINE, J. P., ORMEL, J., POSADA-VILLA, J., SAGAR, R., TSANG, A., USTUN, T. B., VASSILEV, S., VIANA, M. C. & WILLIAMS, D. R. 2010. Childhood adversities and adult psychopathology in the WHO World Mental Health Surveys. *Br J Psychiatry,* 197**,** 378-85.

For peer review only

38. IP, P., WONG, R. S., LI, S. L., CHAN, K. L., HO, F. K. & CHOW, C. B. 2016. Mental Health Consequences of Childhood Physical Abuse in Chinese Populations: A Meta-Analysis. *Trauma Violence Abuse,* 17**,** 571-584.

39. KELLY-IRVING, M. & DELPIERRE, C. 2019. A Critique of the Adverse Childhood Experiences Framework in Epidemiology and Public Health: Uses and Misuses. *Social Policy and Society,* 18**,** 445-456.

40. HOUTEPEN, L.C., HERON, J., SUDERMAN, M.J, TILLING, K., HOWE, L.D., 2018. Adverse childhood experiences in the children of the Avon Longitudinal Study of Parents and Children (ALSPAC). *Wellcome Open Res*.;3:106.

41. GILBERT, R., WIDOM, C. S., BROWNE, K., FERGUSSON, D., WEBB, E. & JANSON, S. 2009. Burden and consequences of child maltreatment in high-income countries. *Lancet,* 373**,** 68-81.

42. KIBURI, S. K., MOLEBATSI, K., OBONDO, A. & KURIA, M. W. 2018. Adverse childhood experiences among patients with substance use disorders at a referral psychiatric hospital in Kenya. *BMC Psychiatry,* 18**,** 197.

43. SIDEBOTHAM, P.D., 2001. An ecological approach to child abuse: a creative use of scientific models in research and practice. *Child Abuse Review*, 10: 97-112.

44. DAHLBERG, L.L., KRUG, E.G., Violence-a global public health problem. In: Krug E, Dahlberg LL, Mercy JA, Zwi AB, Lozano R, eds. World Report on Violence and Health. Geneva, Switzerland: World Health Organization; 2002:1–56.

45. SIDEBOTHAM P, HERON, J and ALSPAC Study Team., 2006. Child maltreatment in the “children of the nineties”: a cohort study of risk factors. Child Abuse and Neglect; 30 (5): 497-522.

46. THOMAS REUTERS, 2018. India: the worlds most dangerous countries for women in 2018. Available from: http://poll2018.trust.org/country/?id=india [Accessed 2nd December 2019]

47. KACKER L, VARDAN S, KUMAR P, 2007. Study on Child Abuse: Ministry of Women and Child Development, Government of India.

48. MACIONNIS, J, PLUMMER, K, 2012. Sociology: a global introduction. United Kingdom: Harlow.

49. SUBRAMANIYAN, V. K. S., REDDY, P., CHANDRA, G., RAO, C. & RAO, T. S. S. 2017. Silence of male child sexual abuse in India: Qualitative analysis of barriers for seeking psychiatric help in a multidisciplinary unit in a general hospital. *Indian journal of psychiatry,* 59**,** 202-207.

50. DEB, S, 2018. An empirical investigation into child abuse and neglect in India: burden, impact and protective measures. Pondicherry: Springer.

51. FLEDDERJOHANN, J., AGRAWAL, S., VELLAKKAL, S., BASU, S., CAMPBELL, O., DOYLE, P., EBRAHIM, S. & STUCKLER, D, 2014. Do girls have a nutritional disadvantage compared with boys? Statistical models of breastfeeding and food consumption inequalities among Indian siblings. *PLoS One,* 9**,** e107172.

For peer review only - http://bmjopen.bmj.com/site/about/guidelines.xhtml

52. AURINO, E. 2017. Do boys eat better than girls in India? Longitudinal evidence on dietary diversity and food consumption disparities among children and adolescents. *Economics & Human Biology,* 25**,** 99-111.

53. KSHATRIYA, G. K. & ACHARYA, S. K. 2016. Gender Disparities in the Prevalence of Undernutrition and the Higher Risk among the Young Women of Indian Tribes. *PloS one,* 11**,** e0158308-e0158308.

54. PILLAI, A., ANDREWS, T. & PATEL, V. 2008. Violence, psychological distress and the risk of suicidal behaviour in young people in India. *International Journal of Epidemiology,* 38**,** 459-469.

55. BALAJI, M., ANDREWS, T., ANDREW, G. & PATEL, V. 2011. The acceptability, feasibility, and effectiveness of a population-based intervention to promote youth health: an exploratory study in Goa, India. *J Adolesc Health,* 48**,** 453-60.

56. JAYA, J. & HINDIN, M. J. 2007. Nonconsensual sexual experiences of adolescents in urban India. *J Adolesc Health,* 40**,** 573.e7-14.

57. BANERJEE, S.R., 2001. Physical abuse of street and slum children of Kolkata. Indian Paediatrics, 38, 1163-1170.

58. SAVE THE CHILDREN, 2015. Forgotten voices; The world of urban children in India. Available from: https://resourcecentre.savethechildren.net/library/forgotten-voices-world-urban-children-india [Accessed 1st March 2020]

59. STROCHLIC, N. 2018. India's forgotten child brides. Available from: https://www.nationalgeographic.com/photography/proof/2018/04/child-brides-marriage-shravasti-india-culture/ [Accessed 5th March 2020]

60. SATHYANARAYANA RAO, T. S., NAGPAL, M. & ANDRADE, C. 2013. Sexual coercion: Time to rise to the challenge. *Indian journal of psychiatry,* 55**,** 211-213.

61. HARBISHETTAR, V. & MATH, S. B. 2014. Violence against women in India: comprehensive care for survivors. *Indian J Med Res,* 140**,** 157-9.

62. HIGGINS, J.P., THOMPSON, S.G., 2002. Quantifying heterogeneity in a meta-analysis. Stats Med; 21; 1539-1558.

63. MOHER, D., LIBERATI, A., TETZLAFF, J. & ALTMAN, D. G. 2009. Preferred reporting items for systematic reviews and meta-analyses: the PRISMA statement. *PLoS Med,* 6**,** e1000097.

64. VERGNES, J. N., MARCHAL-SIXOU, C., NABET, C., MARET, D. & HAMEL, O. 2010.Ethics in systematic reviews. *J Med Ethics,* 36**,** 771-4.

65. WORLD HEALTH ORGANISATION 2018. Adverse Childhood Experiences International Questionnaire. In Adverse Childhood Experiences International Questionnaire (ACE-IQ). Available from: http://www.who.int/violence_injury_prevention/violence/activities/adverse_childhood_experiences [Accessed 1st of February 2019]

66. DAY, C, HIBBERT, P, CADMAN, S, 2008. A literature review into children abused and/or neglected prior to custody. Available from:

For peer review only - http://bmjopen.bmj.com/site/about/guidelines.xhtml

https://dera.ioe.ac.uk/7997/1/Abused%20prior%20to%20custody.pdf [Accessed 5th March 2020]

67. BALDWIN, J. R., REUBEN, A., NEWBURY, J. B. & DANESE, A. 2019. Agreement Between Prospective and Retrospective Measures of Childhood Maltreatment: A Systematic Review and Meta-analysis. *JAMA Psychiatry,* 76**,** 584-593.

68. TEICHER, M. H., SAMSON, J. A., ANDERSON, C. M. & OHASHI, K. 2016. The effects of childhood maltreatment on brain structure, function and connectivity. *Nat Rev Neurosci,* 17**,** 652-66.

69. COLMAN I, KINGSBURY M, GARAD Y, et al. 2016 Consistency in adult reporting of adverse childhood experiences. *Psychol Med*. ;46(3):543-549.

70. EICHENBAUM, H. 2017. Memory: Organization and Control. *Annu Rev Psychol,* 68**,** 19-45.

71. RASCH, B. & BORN, J. 2013. About sleep's role in memory. *Physiol Rev,* 93**,** 681-766.

72. NSPCC 2017. Definitions and signs of child abuse. Available from: - https://learning.nspcc.org.uk/media/1188/definitions-signs-child-abuse.pdf [Accessed 20th December]

73. SINGHI, P., SAINI, A. G. & MALHI, P. 2013. Child maltreatment in India. *Paediatrics and International Child Health,* 33**,** 292-300.

74. SALVADOR-OLIVÁN, J. A., MARCO-CUENCA, G. & ARQUERO-AVILÉS, R. 2019. Errors in search strategies used in systematic reviews and their effects on information retrieval. *Journal of the Medical Library Association : JMLA,* 107**,** 210-221.

75. BEATTIE, T. S., PRAKASH, R., MAZZUCA, A., KELLY, L., JAVALKAR, P., RAGHAVENDRA, T., RAMANAIK, S., COLLUMBIEN, M., MOSES, S., HEISE, L., ISAC, S. & WATTS, C. 2019. Prevalence and correlates of psychological distress among 13-14 year old adolescent girls in North Karnataka, South India: a cross-sectional study. *BMC Public Health,* 19**,** 48.

76. HIGGINS JPT, GREEN S, editors. Cochrane Handbook for Systematic Reviews of Interventions, Version 5.1.0. London: The Cochrane Collaboration; 2011. Section 13.5.2.3. Tools for assessing methodological quality or risk of bias in non-randomized studies.

77. OUZZANI, M., HAMMADY, H., FEDOROWICZ, Z, ELMAGARMID, A., 2016. Rayyan-a web and mobile app for systematic reviews. Systematic Reviews: 5:210.

78. WELLS, G.A., SHEA, B., O'CONNELL. D. PETERSON, J., WELCH, V., LOSOS, M., P.T. 2014. The Newcastle-Ottawa Scale (NOS) for assessing the quality of nonrandomised studies in meta-analyses. Available from: http://www.ohri.ca/programs/clinical_epidemiology/oxford.asp [Accessed 1st of Feb 2019]

79. HERZOG, R., ALVAREZ-PASQUIN, M. J., DIAZ, C., DEL BARRIO, J. L., ESTRADA, J. M. & GIL, A. 2013. Are healthcare workers' intentions to vaccinate related to their knowledge, beliefs and attitudes? A systematic review. *BMC Public Health,* 13**,** 154

For peer review only - http://bmjopen.bmj.com/site/about/guidelines.xhtml

80. DEEKS JJ, HIGGINS JPT, ALTMAN DG (editors). Chapter 10: Analysing data and undertaking meta-analyses. In: Higgins JPT, Thomas J, Chandler J, Cumpston M, Li T, Page MJ, Welch VA (editors). *Cochrane Handbook for Systematic Reviews of Interventions* version 6.0 (updated July 2019). Cochrane, 2019. Available from www.training.cochrane.org/handbook [Accessed 1st of January 2020]

81. NYAGA, V. N., ARBYN, M. & AERTS, M. 2014. Metaprop: a Stata command to perform meta-analysis of binomial data. *Archives of Public Health,* 72**,** 39.

82. HOYSTED, C., BABL, F. E., KASSAM-ADAMS, N., LANDOLT, M. A., JOBSON, L., VAN DER WESTHUIZEN, C., CURTIS, S., KHARBANDA, A. B., LYTTLE, M. D., PARRI, N., STANLEY, R. & ALISIC, E. 2018. Knowledge and training in paediatric medical traumatic stress and trauma-informed care among emergency medical professionals in low- and middle-income countries. *European journal of psychotraumatology,* 9**,** 1468703-1468703.

83. SETH, R & SRIVASTAVA, RN. 2017. Child Sexual Abuse: Management and Prevention, and protection of children from sexual offences (POCSO) Act. Indian paediatrics. 54.

84. PAEZ, A. 2017. Gray literature: An important resource in systematic reviews. *J Evid Based Med*. 10: 233– 240.

85. NOBLIT, G.W, HARE RD, 1988. Meta-ethnography: synthesizing qualitative studies, vol. 11. California: Sage Publications.

86. HANNES, K, AND LOCKWOOD, C., 2012. Synthesising qualitative research: choosing the right approach. John Wiley and Sons.

87. SEERS, K. 2015. Qualitative systematic reviews: their importance for our understanding of research relevant to pain. *Br J Pain,* 9**,** 36-40.

For peer review only

Figure 1. World map showing the rights of a child as measured by UNICEF and World Bank data (2015).

Figure 2. Adverse Childhood Experiences (ACEs) including traditional direct ACEs (child maltreatment: physical, sexual and emotional abuse and neglect) and indirect ACEs (Felitti et al., 1998) and further extended by researchers since the original study (Houtepen et al., 2018)

Figure 3. Potential pathways explaining child maltreatment using the socioecological model proposed by Sidebotham and Heron (2006)

For peer review only

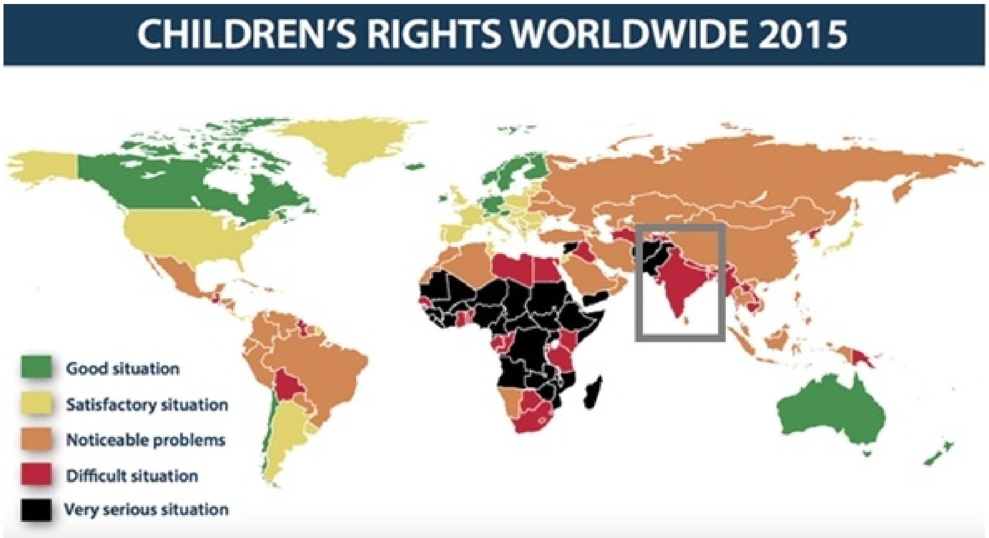

Figure 1. World map showing the rights of a child as measured by UNICEF and World Bank data (2015).

25x13mm (600 x 600 DPI)

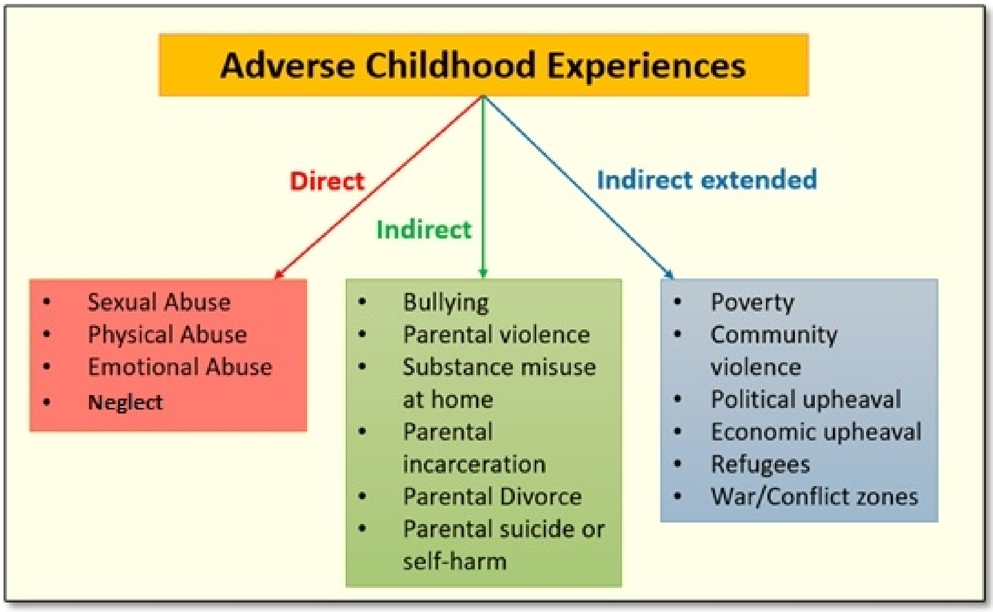

Figure 2. Adverse Childhood Experiences (ACEs) including traditional direct ACEs (child maltreatment: physical, sexual and emotional abuse and neglect) and indirect ACEs (Felitti et al., 1998) and further extended by researchers since the original study (Houtepen et al., 2018)

23x14mm (600 x 600 DPI)

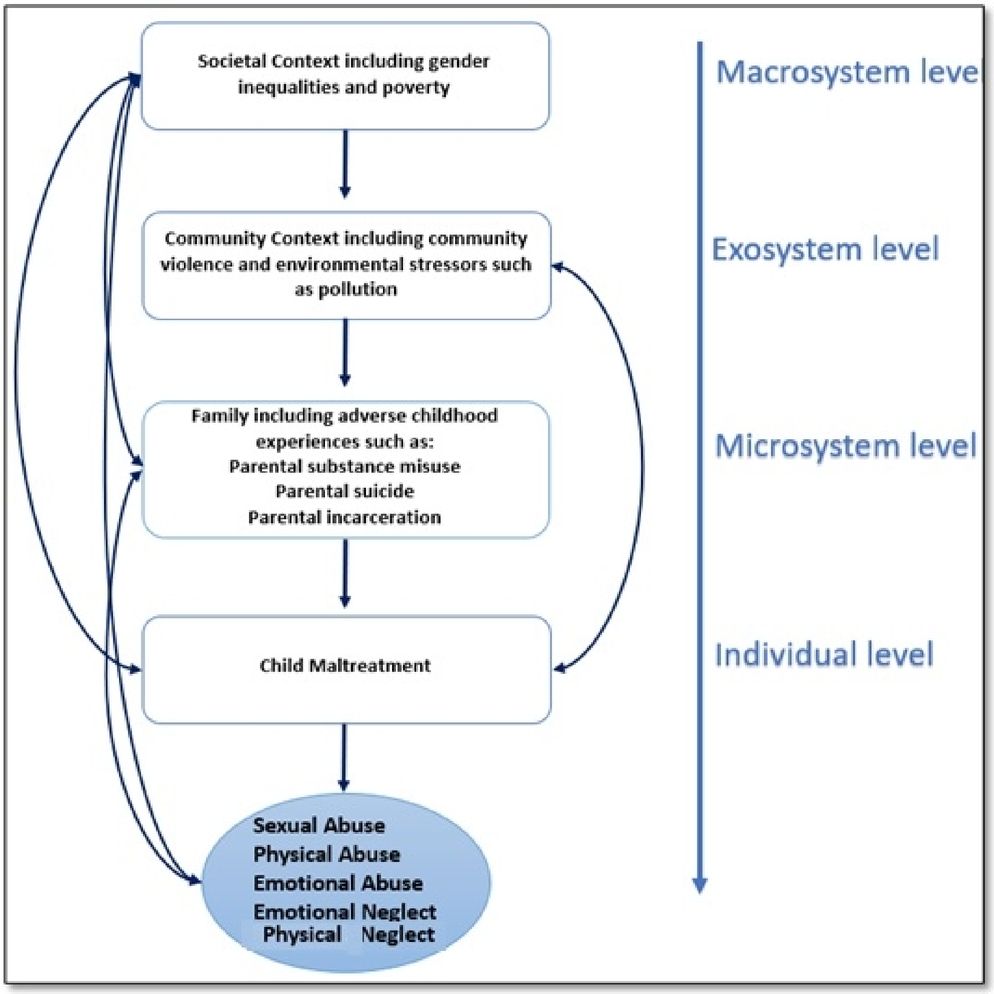

Figure 3. Potential pathways explaining child maltreatment using the socioecological model proposed by Sidebotham and Heron (2006)

22x22mm (600 x 600 DPI)

Appendix 1 - Experts in the field who contributed to the research study design

Member, Designation & Location	Responsibilities
**Nilakshi Vaidya, Psychology Counsellor, National Institute of Mental Health and Neurosciences (NIMHANS) (India) – 2nd** reviewer	Will be the second reviewer for the systematic review and will review 20% of the article titles, abstract and full texts for inclusion criteria. Will be the second author on any journal paper published.
**Megan Fernandes, Core Surgical Trainee with experience working in pediatric medicine and surgery (UK)-3rd** reviewer	Will be the third reviewer for the systematic review and will review 20% of the article titles, abstract and full texts for inclusion criteria (published pre-2012). Will be the third author on any journal paper published.
**Phillip De Souza, Core Internal Medicine Trainee, experience working in internal medicine (UK) -4th** reviewer	Will be the fourth reviewer for the systematic review and will review 20% of the article titles, abstract and full texts for inclusion criteria (published post-2012). Will be the fourth author on any journal paper published.
**Vikas Choudhry, Director, Vice President of Public Health, SRC (Uttar Pradesh)**	Regular contact through the systematic review process. Will input on search strategies and will specifically resolve any conflicts between myself and the second reviewer. Will be the final author (senior author) on any journal publication.
**Bharath Holla, Clinical Psychiatrist and Associate Professor, NIMHANS (India)**	PPI group for input on search words and search strategies
**Vivek Benegal, Clinical Psychiatrist and cVEDA Principal Investigator, NIMHANS (India)**	Clinical psychiatrist and expert in child trauma and informing trauma-based policies in India. Regular contact through the review process and specific input on the search strategies and key words and MESH terms used. Penultimate senior author on any publication.

For peer review only

Appendix 2a. PubMed search strategy

a) Child sexual abuse:

(((((((child sexual abuse) OR child sexual coercion) OR child sexual offender)) OR (((child sexual assault) OR child sexual porn*) OR child sexual molest*)) OR (((((child sexual crime) OR child sexual aggression) OR child sexual offence) OR child sexual violence) OR child rape)) OR ((child rape) OR child incest)) AND India

b) Child emotional abuse:

((((((((((child worthless) OR child unloved) OR child inadequate) OR child failure) OR child corrupt*) OR child unheard) OR child cruelty) OR child emo* AND abuse) OR child maltreat*) AND India)) AND (((((((((((child denial*) OR child denial*) OR child denial shelter*) OR child carer failure) OR child parental failure) OR child emo*) OR child emo* AND danger) OR child neglect) OR child abandonment*) OR child bereavment*) OR child grief) AND India[all])

c) Child physical abuse:

(((((((((((child hitting) OR child shaking) OR child throwing) OR child poisoning) OR child burning) OR child scalding) OR child drowning) OR child suffoc*) OR child beating) OR child physical violence) OR child past*) OR child rap) AND India

Child neglect:

d) (((((child neglect) OR child psycho* AND assault) OR child denial) OR child emo* AND assualt) OR child malnu*) AND India

For peer review only

Appendix 2b EMBASE search strategy

a) Child sexual abuse

1. Prevalence/
2. Limit 1 to human
3. ("India" or "india").mp.[mp=title, abstract, heading word, drug trade name, original title, device manufacturer, drug manufacturer, device trade name, keyword, floating subheading word]
4. (child sexual abuse or "child sexual coercion" or "child sexual offender" or "child sexual assault" or "child sexual porn*" or "child sexual molest*" or "child sexual crime" or "child sexual aggression" or "child sexual offence") or "child sexual violence" or "child rape" or "child rape" or "child incest").mp. [mp=title, abstract, heading word, drug trade name, original title, device manufacturer, drug manufacturer, device trade name, keyword, floating subheading word]
5. 1 and 2 and 3 and 4
6. Limit 5 to yr= "2005 to 2020"

b) child emotional abuse

1. Prevalence/
2. Limit 1 to human
3. ("India" or "india").mp.[mp=title, abstract, heading word, drug trade name, original title, device manufacturer, drug manufacturer, device trade name, keyword, floating subheading word]
4. (child worthless or "child unloved" or "child inadequate" or "child failure" or "child corrupt" or "child unheard" or "child cruelty" or "child emo*" or "child emo abuse" or "child maltreat" or "child denial" or "child denial shelter" or "child carer failure" or "child parental failure" or "child emo*" or "child emo* and danger" or "child neglect" or "child abandonment*" or "child bereavment*" or "child grief")mp.[mp=title, abstract, heading word, drug trade name, original title, device manufacturer, drug manufacturer, device trade name, keyword, floating subheading word]
5. 1 and 2 and 3 and 4
6. Limit 5 to yr= "2005 to 2020"

c) Child physical abuse

1. Prevalence/
2. Limit 1 to human
3. ("India" or "india").mp.[mp=title, abstract, heading word, drug trade name, original title, device manufacturer, drug manufacturer, device trade name, keyword, floating subheading word]
4. (child hitting or "child shaking" or "child throwing" or "child poisoning" or "child burning" or "child scalding" or "child drowning" or "child suffoc*" or "child beating" or "child physical violence" or "child past*" or "child rap")mp.[mp=title, abstract, heading word, drug trade name, original title, device manufacturer, drug manufacturer, device trade name, keyword, floating subheading word]
5. 1 and 2 and 3 and 4
6. Limit 5 to yr= "2005 to 2020"

d) Child neglect
1. Prevalence/
2. Limit 1 to human
3. (“India” or “india”).mp.[mp=title, abstract, heading word, drug trade name, original title, device manufacturer, drug manufacturer, device trade name, keyword, floating subheading word]
4. (child neglect or ”child psycho*” or “assault” or “child denial” or “child emo*” or “assault*” or “child malnu”)mp.[mp=title, abstract, heading word, drug trade name, original title, device manufacturer, drug manufacturer, device trade name, keyword, floating subheading word]
5. 1 and 2 and 3 and 4
6. Limit 5 to yr= “2005 to 2020”

For peer review only

Appendix 2c COCHRANE search strategy

1. India or india
2. (child sexual abuse or child sexual coercion or child sexual offender or child sexual assault or child sexual porn or child sexual molest* or child sexual crime or child sexual aggression or child sexual offence or child sexual violence or child rape or child rape or child incest): ti,ab,kw
3. (child worthless or child unloved or child inadequate or child failure or child corrupt or child unheard or child cruelty or child emo* or child emo abuse or child maltreat or child denial or child denial shelter or child carer failure or child parental failure or child emo* or child emo* and danger or child neglect or child abandonment* or child bereavment* or child grief): ti,ab,kw
4. (child hitting or child shaking or child throwing or child poisoning or child burning or child scalding or child drowning or child suffoc* or child beating or child physical violence or child past* or child rap): ti,ab,kw
5. (child neglect or child psycho* or assault or child denial or child emo* or assault* or child malnu*): ti,ab,kw
6. {#2 or #3 or #4 or #5}
7. #1 and #6

For peer review only

Appendix 2d PsychInfo search strategy

1. Prevalence or epidemiology or frequency.tw.
2. child sexual abuse or child sexual coercion or child sexual offender or child sexual assault or child sexual porn or child sexual molest* or child sexual crime or child sexual aggression or child sexual offence or child sexual violence or child rape or child rape or child incest.tw,sh.
3. child worthless or child unloved or child inadequate or child failure or child corrupt or child unheard or child cruelty or child emo* or child emo abuse or child maltreat or child denial or child denial shelter or child carer failure or child parental failure or child emo* or child emo* and danger or child neglect or child abandonment* or child bereavment* or child grief.tw,sh.
4. child hitting or child shaking or child throwing or child poisoning or child burning or child scalding or child drowning or child suffoc* or child beating or child physical violence or child past* or child rap.tw,sh.
5. child neglect or child psycho* or assault or child denial or child emo* or assault* or child malnu*.tw,sh.
6. India
7. 1 or 2 or 3 or 4 or 5
8. 7 and 6
9. Limit 8 to (English language and yr=”2005-2020”)

For peer review only

Appendix 3 - **Newcastle-Ottawa Scale Quality Assessment**
Prevalence of child abuse in Kerala, India: An ICAST-CH based survey
Manoj Therayil Kumar, Nilamadhab Kar, Sebind Kumar

Selection:

1. Representativeness of the sample:
   a. Truly representative of the average in the target population. * (all subjects or random sampling)
   b. Somewhat representative of the average in the target group. * (non-random sampling)
   c. Selected group of users/convenience sample.
   d. No description of the derivation of the included subjects.

Points: *

2. Sample size:
   a. Justified and satisfactory (including sample size calculation). *
   b. Not justified.
   c. No information provided

Points: -

3. Non-respondents:
   a. Proportion of target sample recruited attains pre-specified target or basic summary of non-respondent characteristics in sampling frame recorded. *
   b. Unsatisfactory recruitment rate, no summary data on non-respondents.
   c. No information provided

Points: *

4. Ascertainment of the exposure (risk factor):
   a. Vaccine records/vaccine registry/clinic registers/hospital records only. **
   b. Parental or personal recall and vaccine/hospital records. *
   c. Parental/personal recall only.

Points: *

Comparability: (Maximum 2 stars)

1. Comparability of subjects in different outcome groups on the basis of design or analysis. Confounding factors controlled.
   a. Data/ results adjusted for relevant predictors/risk factors/confounders e.g. age, sex, time since vaccination, etc. **
   b. Data/results not adjusted for all relevant confounders/risk factors/information not provided.

Points: **

Outcome:

1. Assessment of outcome:
   a. Independent blind assessment using objective validated laboratory methods. **
   b. Unblinded assessment using objective validated laboratory methods. **
   c. Used non-standard or non-validated laboratory methods with gold standard. *
   d. No description/non-standard laboratory methods used.

Points: **

2. Statistical test:
   a. Statistical test used to analyse the data clearly described, appropriate and measures of association presented including confidence intervals and probability level (p value). *
   b. Statistical test not appropriate, not described or incomplete.

Points: *

Total Points scores: 8-star indicating a good study

Cross-sectional Studies:
Very Good Studies: 9-10 points
Good Studies: 7-8 points
Satisfactory Studies: 5-6 points
Unsatisfactory Studies: 0 to 4 points

For peer review only

Appendix 1 – PRISMA Checklist

Section/topic	#	Checklist item	Reported on page #
**TITLE**			
Title	1	Identify the report as a systematic review, meta-analysis, or both.	Page 1
**ABSTRACT**			
Structured summary	2	Provide a structured summary including, as applicable: background; objectives; data sources; study eligibility criteria, participants, and interventions; study appraisal and synthesis methods; results; limitations; conclusions and implications of key findings; systematic review registration number.	Page 2
**INTRODUCTION**			
Rationale	3	Describe the rationale for the review in the context of what is already known.	Pages 3-6
Objectives	4	Provide an explicit statement of questions being addressed with reference to participants, interventions, comparisons, outcomes, and study design (PICOS).	Pages 9
**METHODS**			
Protocol and registration	5	Indicate if a review protocol exists, if and where it can be accessed (e.g., Web address), and, if available, provide registration information including registration number.	Page 2
Eligibility criteria	6	Specify study characteristics (e.g., PICOS, length of follow-up) and report characteristics (e.g., years considered, language, publication status) used as criteria for eligibility, giving rationale.	Pages 10
Information sources	7	Describe all information sources (e.g., databases with dates of coverage, contact with study authors to identify additional studies) in the search and date last searched.	Page 10-12
Search	8	Present full electronic search strategy for at least one database, including any limits used, such that it could be repeated.	Page 12; Appendix 2
Study selection	9	State the process for selecting studies (i.e., screening, eligibility, included in systematic review, and, if applicable, included in the meta-analysis).	Page 13-16

For peer review only

Data collection process	10	Describe method of data extraction from reports (e.g., piloted forms, independently, in duplicate) and any processes for obtaining and confirming data from investigators.	Page 13
Data items	11	List and define all variables for which data were sought (e.g., PICOS, funding sources) and any assumptions and simplifications made.	Page 11
Risk of bias in individual studies	12	Describe methods used for assessing risk of bias of individual studies (including specification of whether this was done at the study or outcome level), and how this information is to be used in any data synthesis.	Page 15
Summary measures	13	State the principal summary measures (e.g., risk ratio, difference in means).	Page 16
Synthesis of results	14	Describe the methods of handling data and combining results of studies, if done, including measures of consistency (e.g., I^2) for each meta-analysis.	Page 16

Section/topic	#	Checklist item	Reported on page #
Risk of bias across studies	15	Specify any assessment of risk of bias that may affect the cumulative evidence (e.g., publication bias, selective reporting within studies).	Page 15
Additional analyses	16	Describe methods of additional analyses (e.g., sensitivity or subgroup analyses, meta-regression), if done, indicating which were pre-specified.	Page 16
**RESULTS**			
Study selection	17	Give numbers of studies screened, assessed for eligibility, and included in the review, with reasons for exclusions at each stage, ideally with a flow diagram.	n/a
Study characteristics	18	For each study, present characteristics for which data were extracted (e.g., study size, PICOS, follow-up period) and provide the citations.	n/a
Risk of bias within studies	19	Present data on risk of bias of each study and, if available, any outcome level assessment (see item 12).	n/a
Results of individual studies	20	For all outcomes considered (benefits or harms), present, for each study: (a) simple summary data for each intervention group (b) effect estimates and confidence intervals, ideally with a forest plot.	n/a
Synthesis of results	21	Present results of each meta-analysis done, including confidence intervals and measures of consistency.	n/a
Risk of bias across studies	22	Present results of any assessment of risk of bias across studies (see Item 15).	n/a
Additional analysis	23	Give results of additional analyses, if done (e.g., sensitivity or subgroup analyses, meta-regression [see Item 16]).	n/a
**DISCUSSION**			

For peer review only

For peer review only - http://bmjopen.bmj.com/site/about/guidelines.xhtml

Summary of evidence	24	Summarize the main findings including the strength of evidence for each main outcome; consider their relevance to key groups (e.g., healthcare providers, users, and policy makers).	Pages 17-18
Limitations	25	Discuss limitations at study and outcome level (e.g., risk of bias), and at review-level (e.g., incomplete retrieval of identified research, reporting bias).	Pages 18
Conclusions	26	Provide a general interpretation of the results in the context of other evidence, and implications for future research.	Page 18
**FUNDING**			
Funding	27	Describe sources of funding for the systematic review and other support (e.g., supply of data); role of funders for the systematic review.	n/a

From: Moher D, Liberati A, Tetzlaff J, Altman DG, The PRISMA Group (2009). Preferred Reporting Items for Systematic Reviews and Meta-Analyses: The PRISMA Statement. PLoS Med 6(7): e1000097. doi:10.1371/journal.pmed1000097

For more information, visit: **www.prisma-statement.org**.

Page 2 of 2

For peer review only
